# Biochemical Characterization of 13-Lipoxygenases of *Arabidopsis thaliana*

**DOI:** 10.3390/ijms221910237

**Published:** 2021-09-23

**Authors:** Daniel Maynard, Kamel Chibani, Sonja Schmidtpott, Thorsten Seidel, Jens Spross, Andrea Viehhauser, Karl-Josef Dietz

**Affiliations:** 1 Department of Biochemistry and Physiology of Plants, Faculty of Biology, University of Bielefeld, 33615 Bielefeld, Germany; daniel.maynard@uni-bielefeld.de (D.M.); kamel.chibani@uni-bielefeld.de (K.C.); sonjaschmidtpott10@gmail.com (S.S.); thorsten.seidel@uni-bielefeld.de (T.S.); andrea.viehhauser@uni-bielefeld.de (A.V.); 2 Faculty of Chemistry/Industrial Organic Chemistry and Biotechnology, University of Bielefeld, 33615 Bielefeld, Germany; j.spross@uni-bielefeld.de

**Keywords:** *Arabidopsis thaliana*, lipoxygenases, polyunsaturated fatty acids, lipoxygenase inhibitor, oxylipin

## Abstract

13-lipoxygenases (13-LOX) catalyze the dioxygenation of various polyunsaturated fatty acids (PUFAs), of which α-linolenic acid (LeA) is converted to 13-S-hydroperoxyoctadeca-9, 11, 15-trienoic acid (13-HPOT), the precursor for the prostaglandin-like plant hormones cis-(+)-12-oxophytodienoic acid (12-OPDA) and methyl jasmonate (MJ). This study aimed for characterizing the four annotated *A. thaliana* 13-LOX enzymes (LOX2, LOX3, LOX4, and LOX6) focusing on synthesis of 12-OPDA and 4Z,7Z,10Z)-12-[[-(1S,5S)-4-oxo-5-(2Z)-pent-2-en-1yl] cyclopent-2-en-1yl] dodeca-4,7,10-trienoic acid (OCPD). In addition, we performed interaction studies of 13-LOXs with ions and molecules to advance our understanding of 13-LOX. Cell imaging indicated plastid targeting of fluorescent proteins fused to 13-LOXs-N-terminal extensions, supporting the prediction of 13-LOX localization to plastids. The apparent maximal velocity (V*_max_* _*app*_) values for LOX-catalyzed LeA oxidation were highest for LOX4 (128 nmol·s^−1^·mg protein^−1^), with a K_m_ value of 5.8 µM. *A. thaliana* 13-LOXs, in cascade with 12-OPDA pathway enzymes, synthesized 12-OPDA and OCPD from LeA and docosahexaenoic acid, previously shown only for LOX6. The activities of the four isoforms were differently affected by physiologically relevant chemicals, such as Mg^2+^, Ca^2+^, Cu^2+^ and Cd^2+^, and by 12-OPDA and MJ. As demonstrated for LOX4, 12-OPDA inhibited enzymatic LeA hydroperoxidation, with half-maximal enzyme inhibition at 48 µM. Biochemical interactions, such as the sensitivity of LOX toward thiol-reactive agents belonging to cyclopentenone prostaglandins, are suggested to occur in human LOX homologs. Furthermore, we conclude that 13-LOXs are isoforms with rather specific functional and regulatory enzymatic features.

## 1. Introduction

Lipoxygenases (LOX, 1.13.11.X) are non-heme iron- or manganese-containing dioxygenases that occur in most eukaryotes and in some prokaryotes, and that play important roles in cell signaling [1]. LOX catalyze the oxidation of polyunsaturated fatty acids (PUFAs) into 1,3-pentadiene-conjugated PUFA hydroperoxides, which can be enzymatically processed by typical and atypical cytochrome P450 monooxygenases (CYP 74s) [2]. In humans, LOX-coupled cascades yield docosahexaenoic acid (DHA)- or arachidonic acid (ARA)-derived neuroprostanes, leukotrienes and prostaglandins of cyclopentanone- and cyclopentenone-types that are involved in development, signaling and defense [3,4]. Similar physiological processes are described in plants and fungi and involve oxidized PUFAs (oxylipins), such as 12-oxo-phytodienoic acid (12-OPDA), methyl jasmonate (MJ), traumatic acid (TA) or green leaf volatiles [5,6,7]. It is assumed that cyanobacteria and algae were the first living systems to utilize oxygen and PUFAs as LOX substrates and that oxylipin diversification was shaped by the evolution of catalase-related enzymes and oxylipin receptors [1,8]. The mentioned species usually express both 9-lipoxygenases (9-LOX) and 13-lipoxygenases (13-LOX), consisting of the catalytic LOX domain and a polycystin-1, lipoxygenase, alpha-toxin and triacylglycerol lipase (PLAT) domain. The role of the PLAT domain in function and evolution remains obscure. Recent reports demonstrated that prokaryotic LOX lacking the PLAT domain are fully active [1], whereas single PLAT proteins exist in eukaryotic organisms such as plants and display similar stress-associated functions as described for LOXs [9]. In mammals and humans (h), LOX12 and LOX15 a/b are 13-LOX-like enzymes in terms of linolenic acid (LeA) hydroperoxidation [10].

In the plant model of *Arabidopsis thaliana* (*A. thaliana*), LeA and linoleic acid (LA) are the major endogenous PUFAs, with percent ratios of LeA/LA near 3.3 in the leaves and 0.7 in roots [11]. Moreover, four 13-LOX isoforms, namely, LOX2, 6, 4 and 3 (ordered according to their relative abundance in the whole plant, see PAXdB https://pax-db.org/, accessed on 28 May 2021) are annotated as being highly selective toward the formation of 13-S-hydroperoxyoctadeca-9, 11, 15-trienoic acid (13-HPOT) from LeA. Among these, Bannenberg et al. [12] revealed a high instability of LOX2 and LOX6, whereas LOX3 and LOX4 were the most active 13-LOXs (for further details, see [12]). The 9-LOX1 and 5 are, however, reported to be active on both LeA and LA with similar activities, as observed for LOX3 and LOX4 [2,12]. Lipases specific for the cleavage of both PUFAs from respective glycolipids and phospholipids (for whole-plant abundancies, see [11]) hardly exist [13]. Therefore, information on how plant-derived 9-LOX and 13-LOX achieve or regulate their specificities in LA or LeA oxidation is incomplete and could be approached in studies similar to those on the hLOX-dependent orchestration of the PUFA metabolism [14].

α-Dioxygenase 1 (DOX1), 9-LOX and 13-LOX channel the PUFA oxidation products into competing metabolic pathways. In addition, at least seven oxylipin-generating enzymes act on 13-HPOT. Among these, the Cyp74s, allene oxide synthase (AOS) and hydroperoxide lyase (HPL) are best described. AOS, in conjugation with the functional redundant allene oxide cyclases 1–4 (AOC), are responsible for the biosynthesis of 12-OPDA, while HPL delivers traumatic acid (TA) and green-leaf volatiles [15]. Depending on the physiological state, 12-OPDA is converted to JA in four reactions. Briefly, 12-oxophytodienoic acid reductase 3 (OPR3) mediates the cyclopentenone (CP) reduction. The carboxyl side-chain is subsequently shortened three times via ATP-dependent CoA-ligases and FA oxidation-related enzymes [15]. Stelmach et al. [16] reported that *A. thaliana*, followed by *Zea mays* and barley, are the angiosperms most rich in 12-OPDA.

While the compartmentation of oxylipin metabolism is understood, the subcellular distribution of oxylipins and intermediates is essentially unknown. If we assume that 12-OPDA is rapidly processed upon export to the extrachloroplast space, the total 12-OPDA contents would reflect the chloroplast pool, allowing estimating chloroplast 12-OPDA concentrations that are close to or higher than 30 µM in these high 12-OPDA species after wounding. Of the 13-LOX enzymes, LOX6 is essential for adjusting the basal 12-OPDA level in roots and leaves [17], while LOX2 serves as the enzyme involved in the bulk synthesis of 12-OPDA in leaves [18]. Ziegler et al. [19] showed the insensitivity of AOS and AOC toward 12-OPDA at 0.5 mM and Ca^2+^ or Mg^2+^ at 1.0 mM, while the effect of 12-OPDA and divalent cations on At-13-LOX activity has not yet been addressed to the best of our knowledge.

It appeared timely to comparatively address and extend knowledge on biochemical properties of 13-LOX first addressed by Bannenberg et al. [12].

## 2. Results

### 2.1. In Silico and Subcellular Targeting of Arabidopsis 13-Lipoxygenases

**Figure 1:**
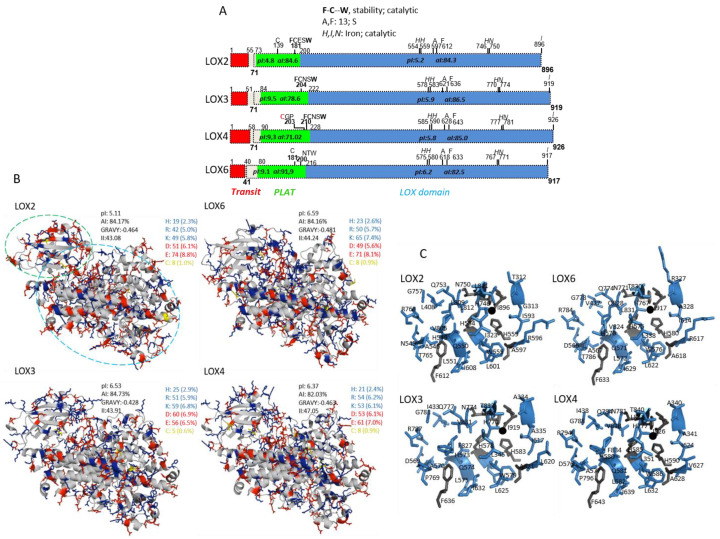
In silico analysis of At-13-LOXs. (**A**) Domain maps of 13-LOXs. (**B**) 3D models of 13-LOX enzymes, highlighting cysteinyl and charged amino acid residues. Tertiary structures of 13-LOX models, based on the crystal structure of SLOX (4wfo), are depicted in grey. Basic (K, R, H) and acidic (D, E) amino acid side-chains (AA) that are positively and negatively charged under physiological pH values are depicted as sticks in blue and red. Of the aliphatic index (AI) determining AA, only Cys (yellow) are shown. The amount and percentage of the pI value determining AA in each 13-LOX, together with Cys residues, are also listed. The PLAT and LOX domains exemplified for LOX2 are circled. (**C**) Active sites (backbone not shown) of 13-LOX are depicted in blue in reference to (**A**), with 13-S determining and catalytic residues highlighted in black.

Based on annotations in publicly available databases and using bioinformatics tools (see Section 4.1), the amino acids sequences of the four 13-LOX isoforms were dissected into putative chloroplast transit sequences, as well as PLAT and catalytic LOX domains (Figure 1A). The 13-LOX sequences differed in length, amino acid composition and physicochemical properties. The calculated isoelectric points (pI) of the LOX domains ranged between 5.2 and 6.2. A major difference was seen for the PLAT domain; while LOX6, LOX4 and LOX3 had a basic pI value (9.1–9.5), the respective domain of LOX2 was strongly acidic (4.8). Distinct characteristics of 13-LOXs were seen when comparing their amino acid sequences, revealing that LOX3 and LOX4 are similar, with 85.0% identity, but only 53% identical to LOX6 and 45% to LOX2, whereas LOX2 is 49% identical to LOX6 [20]. Among the conserved features are metal cofactor-coordinating amino acid residues and the determinants for regio- and stereospecificity (for conventions, see [21]). In addition, the absence of the amino acids typical for manganese LOX, such as Phe 332 or Phe 526, supports 13-LOXs annotation as Fe-LOXs [22]. Interestingly, invariant Trp and one or two Cys residues are located in the PLAT domain of 13-LOX (see domain map Figure 1A). In conjunction with the participation of the PLAT domain in LOX stabilization [23,24,25] and binding to LOX inhibitors, this in silico finding served as a starting point for further studies, as addressed below. Taking into account all active site residues identified or suggested before [20], it is noteworthy that a Pro residue in LOX3 and LOX4 and a Thr residue in LOX2 and LOX6 are in proximity to the 13-S determining Phe (Figure 1B). The 3D models presenting 13-LOXs and their active sites are shown in Figure 1B,C.

**Figure 2:**
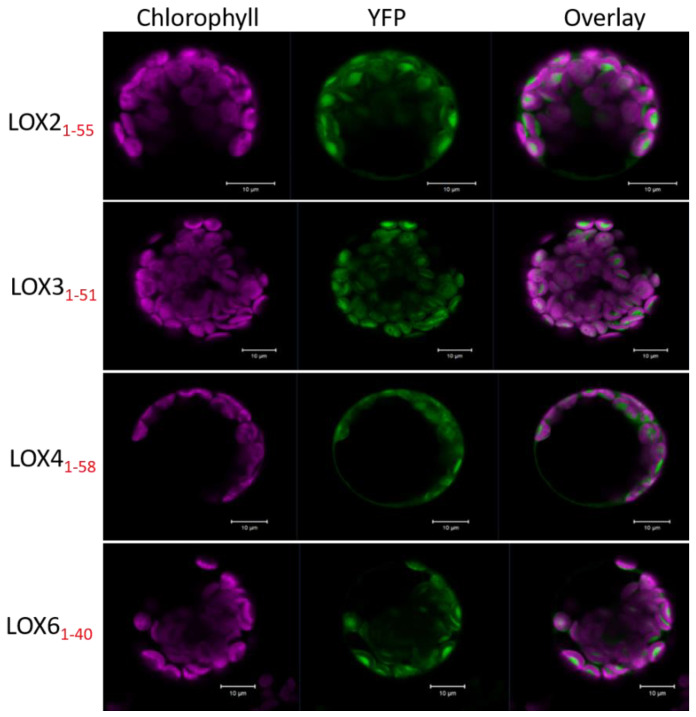
Subcellular localization of fusions of 13-LOXs N-terminal sequences with YFP. Fluorescence microscopic analyses of *A. thaliana* protoplasts transfected with YFP-constructs (LOX2_1-55_-YFP, LOX3_1-51_-YFP, LOX4_1-58_-YFP, LOX6_1-40_-YFP) revealed that the indicated sequences target the reporter to the chloroplast, as shown by the overlay (merge) of the YFP fluorescence signal (green) with chloroplast autofluorescence (magenta). The same pattern of co-localization was observed in several protoplasts. Size bars: 10 μm.

*Arabidopsis* 13-LOX enzymes are most probably plastid-localized, due to their N-terminal extension [2,12,15]. To explore their subcellular targeting, we fused yellow fluorescent protein (YFP) downstream of the putative transit peptide sequences of LOXs, and transiently expressed these constructs in protoplasts isolated from *A. thaliana* leaves in a similar manner, as described for the analysis of rice LOX-1 (Q9FSE5, [26]). As seen in Figure 2, the distribution of the 13-LOX transit peptide:YFP protein fusions matched the pattern of chlorophyll autofluorescence. Merging these images proved co-localization, and indicated that the predicted 13-LOX plastid transit peptides are sufficient to target the reporters to the plastids.

### 2.2. Purification and Enzymatic Characteristics of Arabidopsis 13-Lipoxygenases

To gain further information on biochemical properties, the sequences encoding the mature At-13-LOXs (LOX2_71-896_, LOX3_71-919_, LOX4_71-926_, LOX6_41-917_) were placed as His-tag fusion constructs under the control of the IPTG-inducible promoter in *E. coli* expression vectors. This followed the strategies described in previous reports [21,27,28]. Total proteins were extracted from induced expression strains by mild sonication, and the soluble fusion proteins were obtained in the supernatant containing active LOXs. No activities were observed using control homogenates prepared from *E. coli* cells devoid of LOX plasmids, or when 13-LOX-containing solutions had been boiled (30 s, 85 °C). As shown for LOX6, (see Supplementary Figure S1) this demonstrated that the herein-utilized 13-LOX protein sources were active and specific for PUFA oxidation, containing no impurities that would have caused the unspecific oxidation of PUFA. The theoretical and, thus, expected molecular masses of the recombinant His-tagged 13-LOX were 95,061, 96,791, 97,830 and 100,912 kDa. As judged by SDS-PAGE analysis, dominant bands appeared at the expected mass size (see Figure S2), the proteins were estimated as being of ~90% (LOX4), ~80% (LOX2 and LOX3) and ~50% (LOX6) purity. LOX3, LOX4 and LOX6, unlike LOX2, were enriched by ~5-fold when cleared lysates were purified via metal affinity chromatography. This is demonstrated via SDS-PAGE (Figure S2) and activity analysis (activity analysis is shown for LOX6 in Figure S1). Further attempts to purify 13-LOX were not performed due to our observation of unexpected elution positions during gel-filtration (see Figure S2D) and the instability of 13-LOX [12]. We thus decided to utilize LOX3, LOX4 and LOX6 eluates and LOX2-cleared lysate for subsequent 13-LOX analysis, if not mentioned otherwise.

To analyze the role of 13-LOXs as putative Fe-LOXs (see Section 2.1), we analyzed their iron contents using the ferrozine assay (see Section 4.9), revealing Fe/LOX ratios (mol/mol, means±SD of n ≥ 4) of 0.48 ± 0.1 (LOX2), 0.36 ± 0.1 (LOX3), 0.40 ± 0.1 (LOX4) and 0.27 ± 0.06 (LOX6). The detected iron loads above 20% clearly assign 13-LOXs as Fe-LOXs [29,30], as suggested in Section 2.1.

As chloroplast pH-values range between 7.0 in the dark and >8.0 in the light, we recorded the activities of 13-LOX with their optimal substrate LeA between pH 6.6 and 8.6, to detect the pH-optima of 13-LOX. All members showed high activity around neutral pH and low activity at pH 8.6 (Figure S2C). LOX2 was most sensitive and LOX4 was least sensitive toward pH changes. At pH 6.6, LOX3 and LOX6 activity were lowered by ~30%, in contrast to LOX2 and LOX4, when compared to their optimal pH values at pH 6.6 (3.9 ± 0.4 nmol·s^−1^·mg^−1^) and pH 7.2 (62.3 ± 0.4 nmol·s^−1^·mg^−1^). The apparent affinities of 13-LOX enzymes toward LeA were highest for LOX6 (K*_m app_* = 1.2 ± 0.4 µM) and lowest for LOX2 (K*_m app_* = 26.3 ± 2 µM). The respective velocities of product formation were highest for LOX3 and LOX4 and lowest for LOX2 and LOX6, the latter two had apparent V*_max_*-values~45-fold lower than that of LOX4 (128 ± 18 nmol·s^−1^·mg^−1^), which is partly due to the different degrees of purity. The purities and iron loads below 100% were not considered in the activity calculation or related properties studied herein.

**Figure 3:**
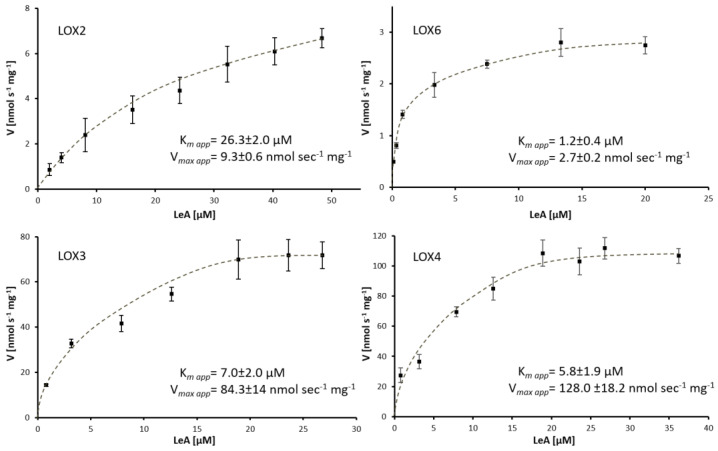
Activity of 13-LOX with their favored substrate, LeA. Graphs are means ± SD of n ≥ 3, derived from a nonlinear regression fit using the Michaelis Menten equation. The apparent (app) value is added to the parameters in order to indicate that 13-LOX enzymes differed in purity and iron load. For more details, see text and Section 4.

The enzymatic parameters of LOX isoforms, with LA as substrate, could only be determined for LOX2 (for more details, see Figure 3 and Figure S3). Relative to LeA, LOX2 was unusually effective in LA- (79%) and ARA-oxygenation (50%) (Table 1).

Regarding LOX2 oxygenates LA at ω-6 (13-HPOD) [12], as revealed from RP-HPLC analysis (Figure S4A), 15-(S)-hydroperoxy-5(Z),8(Z),11(Z),13(E)-eicosatetraenoic acid (15-HPETE) was the oxylipin predominantly formed by LOX2 from ARA and displayed retention times (Rt) of 12.5 min (Rt 15-HPETE 12.5 min). As shown spectrophotometrically, all three LOX2-derived peroxides were metabolized by AOS (Figure S4B), which is supportive to the turnover of ω-6 oxygenated PUFAs by AOS of rice and *Zea mays* [31,32].

We intentionally tested whether the 22:6 (ω-3) PUFA DHA is a substrate of 13-LOX. As shown in Table 1, 13-LOX enzymes dioxygenated DHA, with rates of ~7 to 56% relative to LeA, of which LOX2 was the most active enzyme. In line with the previous data on SLOX and LOX6 [33], a dominant oxylipin derivative of DHA, after incubation of DHA with LOX2, fractioned at Rt ~13 min. The MS analysis of this fraction revealed the presence of HPDH, as indicated by the major signal appearing at *m*/*z* of 383.2 due to the association of HPDH (MW = 360.5 g·mol^−1^) with sodium [M+Na]^+^. The molecular ion was subjugated to an MS^2^ experiment and the formation of the fragment ion *m*/*z* 297 confirmed the position of hydroperoxidation at ω-6, due to the loss of an 86 Da fragment (C_5_H_10_O), in accordance with the findings of [33]. This hypothesis was further endorsed by the confirmation of the sum formulas of the molecular ion and the *m*/*z* 297.2 fragment ion (Figure S5 and Table S2). The specificity of LOX3 and LOX4 was rather poor for producing 17-HPDH (see Figure S5). We observed that 13-LOXs were highly sensitive toward PUFAs or their oxidation products, whereas LOX2 was most robust. As analyzed for their favored substrates, more than 50% inhibition occurred when the enzymes (0.22 µg/µL) were pre-incubated with 29 µM LA (LOX6) or 118 µM LA (LOX3 and LOX4). The effect of LA and LeA on 13-LOX inhibition is depicted in Figure S6, revealing similar trends when pre-incubated with LeA.

### 2.3. Arabidopsis 13-LOX Isoforms Mediate the Synthesis of 12-OPDA and OCPD

**Figure 4:**
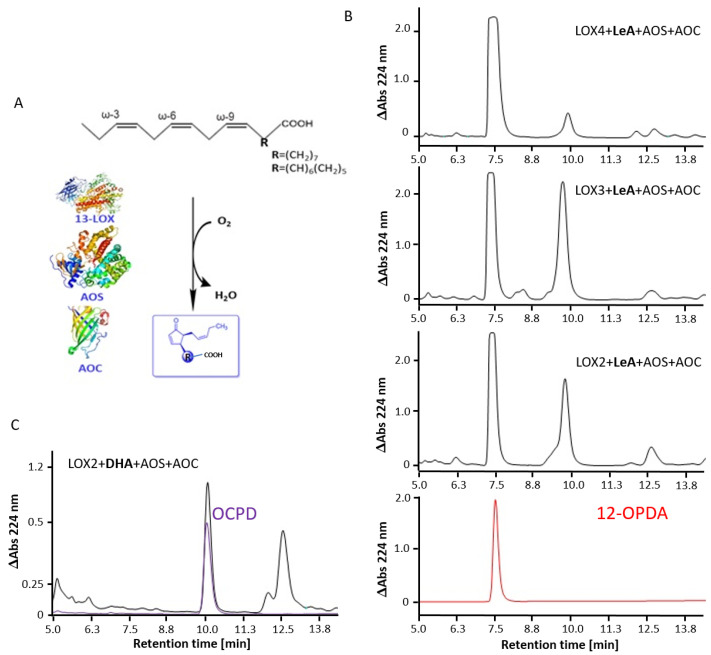
The 13-LOX mediated synthesis of 12-OPDA and OCPD. (**A**) Flow scheme for anticipated C18 and C22 CP synthesis from ω-3 PUFAs LeA and DHA, with 3-D models of LOX2 (based on SLOX, 4wfo), AOS (3dsk) and AOC (1z8k). (**B**) RP-HPLC analysis of 12-OPDA and (**C**) OCPD one-pot synthesis extracts, the peak of high intensity appearing at 7.4–7.5 min and 10.1 min corresponded to LeA- and DHA-derived 12-OPDA and OCPD, as revealed by a comparison with the retention times of standards.

13-HPOT is the substrate for the subsequent synthesis of 12-OPDA. As shown previously for LOX6 [33], 12-OPDA was produced by all At-LOX lysates, coupled to AOS and AOC, as revealed by the comparison of retention times with the standard (Figure 4).

The result underscores the involvement of 13-LOXs in 12-OPDA synthesis and the potential mechanism of feedback modulation by 12-OPDA, as investigated below. Due to the aforementioned criteria, LOX2 was, thus, utilized for OCPD synthesis. As revealed by the comparison of retention times with the OCPD standard, LOX2 like SLOX and LOX6, coupled in a one-pot synthesis with AOS and AOC [33], enabled the formation of the promising bioactive compound OCPD from DHA.

### 2.4. Effect of Metal Ions and LOX-Associated Phytohormones on Arabidopsis 13-LOX Activities

Ca^2+^ and Mg^2+^ play important roles in enzyme regulation. Stromal Mg^2+^ increases in the light phase, when the pH is >8.0 [34]. In addition, the activities of mammalian and plant lipoxygenases are affected by divalent cations [23,35,36]. Therefore, we investigated whether the same result occurs for 13-LOXs and included activity studies with Cu^2+^ and Cd^2+^. At a tested concentration of 1 mM, 13-LOX enzymes were differentially stimulated by Ca^2+^ and Mg^2+^. In comparison to control (mean values n ≥ 3), LOX2 activity increased more in the presence of 1 mM Ca^2+^ (590%) than 1 mM Mg^2+^ (70%). LOX4 and LOX3 were slightly activated (6% and 35%) by Mg^2+^, whereas Ca^2+^ stimulated LOX3 activity. Ca^2+^ and Mg^2+^ enhanced LOX6-catalyzed oxygen consumption by 21% and 42%, respectively (Table 2).

The activity of 13-LOXs was abolished (LOX3, LOX4, LOX6) or strongly decreased (LOX2) by the addition of 100 µM Cu^2+^.

In contrast to the inhibition of LOX3, 4 and 6 in the presence of 1 mM CdCl_2_, the rate of oxygen consumption increased when LOX2 was injected into LeA supplemented with 1 mM Cd^2+^ (Table 2). The concentration dependency of Cd^2+^ on LOX2 activity was analyzed spectrophotometrically (Figure S6B), revealing the following values (nmol·s^−1^·mg^−1^, mean ± SD of n ≥ 3): 0.8 ± 0.2 for control, 2.3 ± 0.3 in the presence of 40 µM Cd^2+^, 5.2 ± 0.9 in 150 µM Cd^2+^ and 5.7 ± 0.7 in 1180 µM Cd^2+^. With regard to applications, e.g., for the optimization of 13-LOX activity in 12-OPDA synthesis, we were interested in exploring whether 13-LOXs are also activated when lysed in buffer without supplements, as utilized in 12-OPDA synthesis (see Section 4.8). As suggested from the dialyzed LOX2 lysate, all 13-LOXs except LOX2 were inhibited by Cd^2+^, whereas Ca^2+^ or Mg^2+^ stimulated LOX3 and LOX6 activity, in line with Table 2 (see Table S3).

The next assay addressed the hypothesis that 13-LOX may be inhibited by its product 12-OPDA, as one possible mechanism of feedback regulation. We incubated 12-OPDA with 13-LOXs and analyzed the results for the remaining LOX activity. The non-physiological Michael acceptor N-ethylmaleimide (NEM) was also included as a control to support any observations made with MJ and 12-OPDA. As seen in Table 3, a decrease in LOX activity occurred in the presence of 12-OPDA, which was less pronounced with the 12-OPDA-derivative MJ. MJ was also tested to reveal insights into the selectivity and feedback modulatory aspects: (i) the cyclopentanone-like-prostaglandin is a model jasmonate-like compound devoid of α,β-unsaturated carbonyl system, unique to Michael acceptors (for more details, see [37]); (ii) MJ was revealed to enhance the transcription of LOX2 and LOX3 (see Section 3.5). In several systems, endogenous JA accumulation is inhibited by the application of salicylic acid (SA) to plants [19]. Therefore, we also explored whether this phytohormone, in addition to the LOX-HPL-derived TA, affects 13-LOX activity in vitro. The first tests did not result in any significant effects of SA and TA on LOX activity. To augment these observations, LOX activity was spectrophotometrically assayed in presence of reactive molecules. Due to the 10-fold higher sensitivity of detection [38], we used less enzyme but kept the same compound concentrations of 280 µM and 4.8 µM LeA. Here, NEM, 12-OPDA and MJ (except for LOX4) diminished LOX activity more intense, whereas the effects of the other tested chemicals on 13-LOX were in the same range as recorded polarographically (see Table 3).

**Figure 5:**
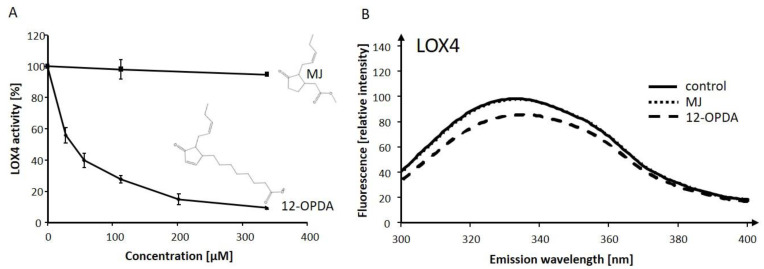
Analysis of the interaction of 12-OPDA and MJ with LOX4. (**A**) Dose-dependent inhibitory effects of 12-OPDA and MJ on the activity of LOX4. LOX4 (0.22 µg/µL) was incubated with compounds at indicated concentrations for 10 min at 30 °C, and residual LOX activity was measured spectrophotometrically by the injection of 3 µL incubation mixture into 123 µL LeA (4.8 µM in 50 mM Tris-HCl, pH 7.2). Values (nmol·s^−1^·mg^−1^, means ± SD (n = 3)) are given relative to LOX4 activity (38.0 ± 2.0) obtained with solvent (control). (**B**) Intrinsic fluorescence of LOX4 incubated with 12-OPDA, MJ and solvent (control). Here, 120 µL LOX4 (0.2 µg/µL) was mixed with 15 µL chemical (1.4 mM in 50 mM K-Pi, pH 8.0) or solvent (control) and analyzed for intrinsic fluorescence emission after excitation at λ_Exc_ = 280 nm. Mean graphs are shown (n = 4).

We concluded that of the three compounds derived from 13-LOX, only MJ and 12-OPDA were inhibitory on LOX activity, supporting a model of feedback inhibition. To gain additional biochemical information, we aimed at determining enzyme inhibitory constants. Furthermore, we analyzed whether MJ and 12-OPDA alter the LOX protein conformation, which is often accompanied by protein functional changes [33]. Incubation of LOX4 (0.22 µg/µL) with indicated amounts of chemicals revealed the inhibition of LOX4 by 12-OPDA, with an IC_50_ value of 48 ± 3 µM (Figure 5A). Incubation of LOX2, LOX3 and LOX6 with 12-OPDA revealed that a level of 108 µM significantly (*p* ≤ 0.05) decreased their activities in comparison to the control (see Figure S7A). An IC_50_ value of 130 ± 31 µM was estimated for LOX3. Due to the effects of pH, reliable IC_50_ values could not be derived for LOX2 and LOX6; however, as inferred from the dose-response curves and data shown in Table 3, these IC_50_ values likely range between 280 and 338 µM.

Intrinsic fluorescence studies of LOX4 (see Figure 5B) revealed a decrease in fluorescence emission in the presence of 156 µM 12-OPDA. At the LOX4 fluorescence emission maxima of 333 nm (λ_Exc_ = 280 nm), which was not altered by the indicated chemicals, the relative fluorescence emission mean values (± SD of n = 4) were as follows: 98.1 ± 3.9 (control), 86.2 ± 3.9 (12-OPDA), 98.0 ± 2.4 (MJ). Thus, a change in the intrinsic fluorescence of LOX4 was not observed when incubated with MJ. Unlike LOX4, MJ altered the intrinsic fluorescence of LOX2 and LOX3 and, as observed by photometry, the activity decreased by more than 40% at 280 µM MJ (see Table 3 and Figure S7B) in comparison to 7%, as observed for LOX4 at 338 µM (see Figure 5A). To further evaluate the mechanism of inhibition of 13-LOXs by MJ and 12-OPDA on 13-LOXs, kinetic studies were undertaken. Michaelis–Menten plots and the corresponding Lineweaver–Burk double reciprocal plots at increasing inhibitor and substrate concentrations are presented in Supplementary Figures S8–S10. The Lineweaver–Burk plot of 12-OPDA against LOX2 revealed a decrease in the velocity of the reaction when the enzyme was saturated by substrate at apparent Vmax-values. Together with the change in the apparent Michaelis–Menten-constant, 12-OPDA tested at 226 µM was consistent with a non-competitive mode of LOX2-inhibition. As for MJ, the intersecting lines on the *y*-axis were only slightly changed, whereas the slope was increased in comparison to control and 12-OPDA incubates. The result is in line with a competitive effect of the cyclopentanone on LOX2.

**Figure 6:**
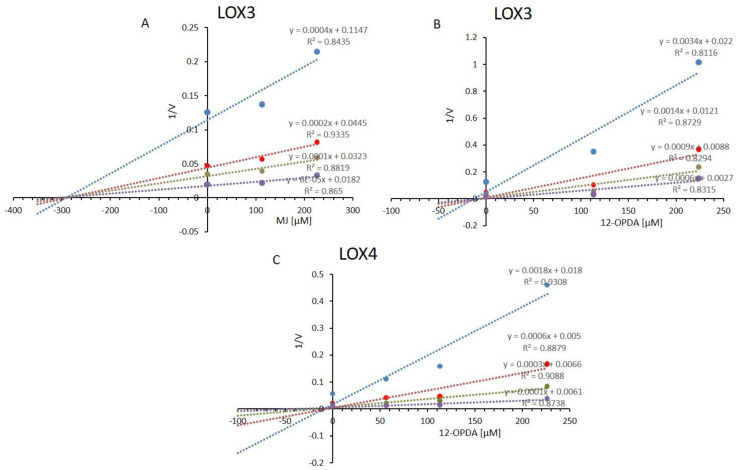
Dixon plots for the inhibition of LOX3 by MJ (**A**) and 12-OPDA (**B**) and LOX4 by 12-OPDA (**C**) Blue symbols and fitted straight lines represent enzyme activity with 1.6 µM substrate, while red, green and violet represent enzyme activities with 4.8 µM, 12.8 µM and 25.6 µM LeA. As for LOX4, blue symbols and fitted straight lines represent enzyme activity with 1.6 µM substrate, while the red, green and violet represent enzyme activities with 9.6 µM, 16 µM and 32 µM LeA. Analysis of pre-incubated protein (0.22 mg/mL), at 30 °C (control, solvent of 12-OPDA or MJ), was performed as described in Section 4.6. Values were obtained from the data presented in Figures S9 and S10.

As for LOX6, the identified inhibitor 12-OPDA was non-competitive at concentrations of 226 µM (see Figure S8). As for LOX3, MJ only minimally affected the apparent Km-values, ranging between 10 µM and 11 µM, in comparison to the control (no effector present) value of 11 µM, observed with a Lineweaver–Burk plot, and 9 µM with Michaelis–Menten plot. The apparent Vmax values declined from 66 µM (control) to 39 µM, with increasing MJ. The Dixon plot for MJ produced intersecting lines on the *x*-axis, confirming the non-competitive mode of inhibition and providing a Ki value of 295 µM (Figure 6A). In short, 12-OPDA acted in a non-competitive or mixed mode. As seen in the respective Michaelis–Menten and Lineweaver–Burk-plots, both the apparent Vmax and Km-values were affected. The Dixon plot (Figure 6B) was in line with a noncompetitive mode of inhibition, with an estimated Ki-value of 13 µM, which is in disagreement with the titrated IC_50_-value of 130 µM. As for LOX4, the Dixon plot revealed a Ki-value of 10 µM, similar to that of the observed IC_50_ value (Figure 6C).

By considering the Michaelis–Menten curves (Figure S10A), where I = Ki would reveal half-maximal activity for noncompetitive inhibition, the value of 10 µM seems overestimated. The Lineweaver–Burk plot revealing a decrease in apparent V_max_- and an increase in apparent Km-values, together with the Dixon plot, supported the conclusion that 12-OPDA was either a non-competitive or mixed LOX4 inhibitor. Slight decreases in apparent V_max_ values might be due to minor variations in enzyme quality or the pre-incubation procedure at 30 °C, as the activities of 13-LOXs decrease at elevated temperatures. LOX6 was most sensitive, as depicted by the half-life values in Figure S11.

To translate our findings on the impacts of 12-OPDA on 13-LOX to in vivo conditions, we assayed root and leaf extracts from *A. thaliana* for LOX activity with no positive results, as was in line with previous reports [39]. An alternative source of 13-LOX is the dicot vegetable pea [40]. We detected pea LOX protein by antibody staining and activity in enzyme tests in young roots; that is, the organ depleted of LOX inhibitors, in contrast to leaves [41] (Figure S12). The oxygen-electrode recording with 36 µg pea root extract, pretreated with 1 mM 12-OPDA, revealed an inhibition of activity by about 43% (2.0 ± 0.5 nmol·mL^−1^·min^−1^, n = 4) relative to the control (4.6 ± 0.5 nmol·mL^−1^·min^−1^, n = 4). Thus, 12-OPDA may also inhibit 13-LOX in vivo.

### 2.5. The Role of Cysteine Residues in Activity Regulation of AtLOX4

**Figure 7:**
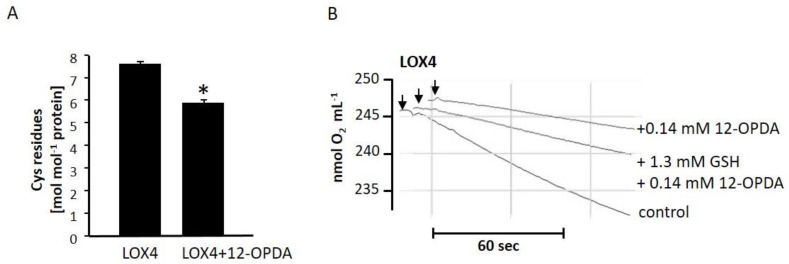
Involvement of thiols in the interaction of LOX4 with 12-OPDA. (**A**) Free thiols of LOX4 incubated with 12-OPDA. Asterisks indicate a significant difference (*p* ≤ 0.05) in comparison to control incubate (12-OPDA solvent), calculated using one-way ANOVA with post hoc Tukey HSD. (**B**) O_2_-electrode recordings on the effect of GSH on the efficacy of the herein-identified LOX inhibitor 12-OPDA. GSH or GSH solvent (40 mM K-Pi, pH 8.0) was pre-incubated for 5 min with LOX4 before 12-OPDA as indicated by the arrows was added to the mixture. After 10 min, the incubation mixture was injected into 1.8 mL 20 µM LeA at 25 °C. The recording of LOX4 incubated with 40 mM K-Pi, pH 8.0 and solvent is shown as a control. The three recordings were performed thrice with the same result.

To analyze whether cysteinyl thiols of 13-LOX are involved in activity regulation, we focused on LOX4. At first, we were interested in whether OPDA covalently modifies the cysteines of LOX4, and whether this type of reaction might be the reason for activity impairment. Here, we determined its free thiol content using the method adopted by Ellman (Figure 7A). Essentially, all eight Cys residues (Figure 1B; 7.6 ± 0.1 mol·mol^−1^, n = 4) were detected in the reduced state. After incubation with 12-OPDA, six Cys residues (5.9 ± 0.1, n = 4) remained, suggesting that 1–2 reactive thiols are involved in LOX4 activity modulation by 12-OPDA. GSH potentially reacts with 12-OPDA in a nucleophilic addition reaction and could decrease or even abolish 12-OPDA reactivity. To reveal whether the thiol antioxidant GSH protects 13-LOX from inactivation by the cysteine-reactive 12-OPDA, we incubated 12.2 µg LOX4 with 1.3 mM GSH, prior to the addition of 140 µM 12-OPDA, and analyzed the results for residual activity. In comparison to the control (LOX4: 9.8 ± 0.1 nmol·mL^−1^·min^−1^ ≙ 100%, n = 2), GSH showed a minor protective effect on LOX4 against 12-OPDA, namely, by ~7.1% (3.6 ± 0.1 nmol·mL^−1^·min^−1^ ≙36.7%, n = 2) compared to incubation with 12-OPDA alone (2.9 ± 0.1 nmol·mL^−1^·min^−1^ ≙29.6%, n = 2; see Figure 7B).

Inhibition remained almost unchanged after doubling the LOX4 concentration. In this case, GSH was protected by ~11.7% (LOX4: 19.7 ± 0.1 nmol·mL^−1^·min^−1^ ≙100%, n = 4, LOX4 + 140 µM 12-OPDA: 6.7 ± 0.1 nmol·mL^−1^·min ≙34%, n = 2, LOX4 + 1.3 mM GSH + 140 µM 12-OPDA: 9.0 ± 0.1 nmol·mL^−1^·min^−1^ ≙45.7%, n = 2). In addition, a twofold increase of GSH concentration failed to protect LOX (LOX4 + 2.6 mM GSH + 140 µM 12-OPDA 8.5 ± 0.6 nmol·mL^−1^·min^−1^, n = 3). Only the extended pre-incubation of 12-OPDA with GSH protected LOX4 from inactivation by 12-OPDA (see Figure S13), demonstrating that the electrophilic α,β-unsaturated carbonyl system of 12-OPDA is likely to be involved in the inhibition of LOX4 activity caused by 12-OPDA.

**Figure 8:**
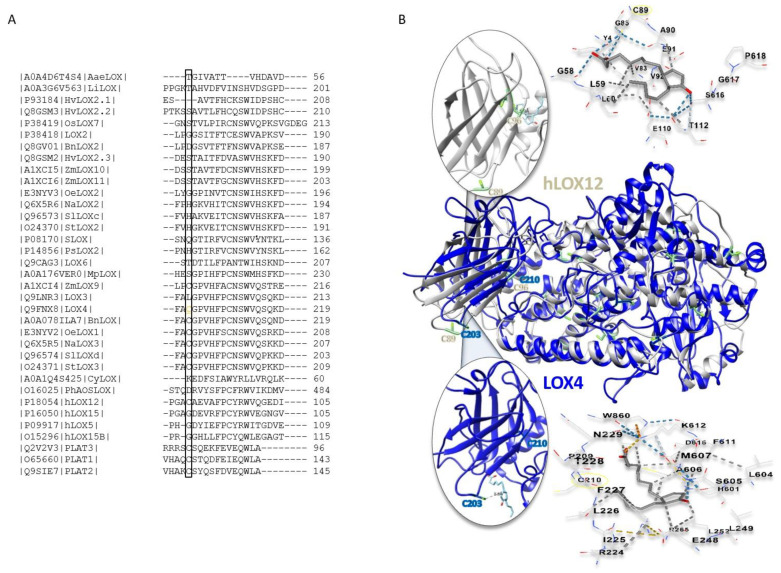
In silico analysis of LOX4, related proteins, and their interaction with the 12-OPDA. (**A**) Cys203 locates in the PLAT domain of LOX4 and is present in hLOX12, PLAT proteins and plant 13-LOXs, as revealed by multiple sequence alignment (not fully shown) with LOXs from fungi (*Agrocybe aegerita*, Aae), algae (*Lobosphaera incisa*, Li), cyanobacteria (*Calothrix* sp. *HK-06*, Cy), moss (*Marchantia polymorpha*, Mp), anthozoa (*Plexaura homomalla*, Ph), monocot (*Oryza sativa*, Os, *Zea mays*, Zm, *Hordeum vulgare*, Hv) and dicot plants (*Glycine max*, S, *Brassica napus*, Bn, *Olea europaea*, Oe, *Pisum sativum*, Ps, *Nicotiana attenuata*, Na, *Solanum lycopersicum*, Sl, *Solanum tuberosum*, St) and humans (h). Codes represent Uniprot/SwissProt IDs. (**B**) Structural alignment of LOX4 with LOX12, and blind docking studies with LOX4 and hLOX12, revealed that PLAT Cys (shown as sticks) are similarly positioned (<6 Å) and are addressed by 12-OPDA, with distances not exceeding 6Å (shown as a blue and grey stick model). The models were obtained by in silico studies with Swiss Dock and CB-DOCK online tools. One of the top 10 and top 5 possible docking modes is presented. LOX domains are not fully shown. The remaining Cys residues and the LOX stabilizing interaction between LOX4Trp213 and LOX4Arg327, Trp99 and Arg165 in hLOX12 are also presented with side-chains highlighted.

The PLAT domain is involved in LOX activity regulation. In human hLOX5, the PLAT domain is the reaction site of inhibiting Michael systems [42,43]. Sequence alignment of the mentioned LOXs and annotated plant 13-LOXs revealed that two cysteine residues in the respective PLAT domain partially align (Figure 8A). The thiol-bearing F-C-W motif is involved in the stability and catalysis of LOXs [24,25] and is absent in LOX6. Furthermore, the N-terminal Cys203 of LOX4 is also present in hLOX12 in other plant LOX, e.g., *Zea mays*, and in PLAT 1–3. Among the At-13-LOXs, LOX4 displays the highest sequence identity with hLOXs, this being 26.43% and 27.34% to LOX5 and LOX12 (supplementary File S1).

Structural alignment of LOX4 with LOX12 predicts that the Cys residues of the PLAT domain are located at similar positions. Furthermore, blind docking studies employing the CB-DOCK and Swiss Dock online tools revealed interdomain interaction with 12-OPDA (Figure 8B), similar to that reported for hLOX5 with 3-acetyl-11-keto-beta-boswellic acid [43]. Based on these results with LOX4, namely, its sensitivity to thiol modifications, the intrinsic fluorescence change, and in silico analyses, it appeared that C203 might be involved in activity regulation. Therefore, C203 of LOX4 was replaced with Ser by site-directed mutagenesis. Unfortunately, we were unsuccessful in obtaining the site-directed variant of 13-LOX as immunoreactive heterologously expressed protein (data provided on request).

## 3. Discussion

### 3.1. Synthesis of Functional 13-LOX Isoforms

In this study, *Arabidopsis* 13-LOXs could be obtained from an *E. coli* protein expression system, previously derived from an insect expression system used by Bannenberg et al. [12]. Despite the disadvantage of utilizing partially purified proteins for biochemical characterizations, the data presented herein extend the molecular characteristics of At-LOX, first provided by Bannenberg et al. [12]. Difficulties in the synthesis of functional 13-LOX or hLOXs were observed in previous studies and the utilization of enzyme-containing lysates or partially purified LOXs is not unusual [12,14,44,45] when gaining the biochemical characteristics of LOXs. The iron loads and enzyme purities of 13-LOXs were not taken into consideration in the determination of activity characteristics; thus, the activities are apparent values that might alter if isolated with iron loads of 100% and increased purities, especially for LOX6. Their apparent K*_m_* and V*_max_* activity values with the favored substrates, LA or LeA, are similar when compared to the 13-LOXs of other plant sources, for example, tomato, banana, melon and soybean, with values between 1.4 and 200 µM [21,46,47] and hLOXs [48].

### 3.2. Subcellular Localization of 13-LOX Isoforms

*Arabidopsis* 13-LOXs and orthologs are predicted to be located in plastids [2,12,15]. This was previously indicated in proteomics studies for LOX2 and LOX6 [49]. Accordingly, the putative transit peptide (Figure 1A) of each *A. thaliana* 13-LOX isoform was sufficient to target YFP to the chloroplasts in transfected protoplasts (Figure 2); thus, 13-LOXs are likely to reside in plastids. However, it is likely that 13-LOXs locate to non-photosynthetic organelles as well, as shown for the LOX6 and LOX2 that were also identified in root plastids [17] and nucleus [50].

### 3.3. Substrate Specificities and Evolutionary Relations

As reported in [12], LOX3, LOX4 and LOX6 were maximally active at neutral pH (pH 7.2). In slight contrast to our data, [12] reported LOX2 activities below 50% with LA (~40%) and ARA (~15%) and revealed no pH optimum. This might be due to different methods for protein and substrate preparation. Our observation of LOX2, as being an enzyme with good peroxidation activity toward ARA and LA, fits into its evolutionary relatedness to potato LOX2 (O24370) and the rice leaf pathogen-inducible lipoxygenase (OsLOX7, P38419), shown to have similar activities toward ARA and LA [31,51]. The identification of ω-6 DHA peroxidation might indicate that LOX2 is closer to hLOX15 than to hLOX12, which is also corroborated by their sequence similarities of 26.99 vs. 25.55% (See Supplementary File S1). LOX15 acts as ω-6 LOX toward both LeA and DHA. The 3D modeling and the protein alignment of LOXs suggest that Pro residues (P769 and P796 in LOX3 and 4) are present in the active site and, together with Cys 203 (according to LOX4), could play decisive roles in differentiating LOX3/4 from LOX2/6 functional characteristics in plants. The lack of these particular prolyl and cysteinyl residues assigns these 13-LOX to a group different from LOX3 or LOX4 (see Figure 1), e.g., AtLOX2 and StLOX2, in contrast to AtLOX4, with StLOX3 (O24371), as explored in Supplementary File S1.

### 3.4. Differential Impact of Divalent Cations on 13-LOX Isoforms

Apart from the above outlined enzymatic characteristics, clear differences were also observed for 13-LOX sensitivity to alkaline earth metals, as previously hypothesized by [52]. LOX2 and LOX6 were strongly activated by Ca^2+^ and Mg^2+^, whereas LOX4 and LOX3 were only moderately or not activated by these ions. Whether this is due to the high number of acidic residues (Figure 1B) that often bind Ca^2+^ and Mg^2+^ [53] or owing to Ca^2+^/Mg^2+^-binding motifs, as observed for human LOXs [25], awaits elucidation (see Supplementary File S1). In planta, Cd^2+^ and Cu^2+^ affect the oxylipin blend [54] and abundance of 13-LOX transcripts [55,56]. We report that 13-LOXs are all inhibited by Cu^2+^, while Cd^2+^ stimulated LOX2 activity, similar to Ca^2+^ (Table 2). Ca^2+^ and Cd^2+^ have partly similar properties [53]. These in vitro data may provide an explanation for the earlier findings of Montillet et al. [57], who observed that Cd^2+^ increased the content of 13-HPOT in *A. thaliana* and might relate to the specific activation of LOX2 by Cd^2+^ in vivo.

### 3.5. Suicide Inhibition and Feedback Regulation

Wu [58] and Conrad [59] reported some sort of catalytic self-inhibition of SLOX and hLOX12/15 by their substrates. Likewise, we noted the enzyme inhibition of LOX3, LOX4 and LOX6 when incubated with LA or LeA, at concentrations enabling high LOX2 activity (see Figure S6A). This might imply the PUFA-dependent regulation of the 9-LOX (LA-specific, [12]) and/or 13-LOX (LeA-specific, [12]) pathways in vivo. The results support the view that all 13-LOXs potentially channel 13-HPOT in 12-OPDA biosynthesis, as anticipated for LOX6 in leaves and roots, LOX2 in leaves, and LOX3 and LOX4 (shown for JA) in inflorescences and wounded leaves [17,20,60].

Similar to hLOX5, hLOX12, pea and sLOXs [58,61,62,63], we observed that cysteinyl residues are involved in the activity of 13-LOXs, as demonstrated by inhibition with NEM. As listed in Table 3, the physiological Michael acceptor 12-OPDA likewise decreased LOX activities. This is in accordance with a model of thiol-sensitive feedback inhibition. From the dose-response curves shown in Figure 5 and Figure S7, the IC_50_ values were estimated both below and above 50 µM 12-OPDA for 13-LOXs. The higher concentration of 12-OPDA required to inhibit LOX2, LOX3 and LOX6, in comparison to LOX4, might be due to differential 12-OPDA sensitivities.

The gene expression of LOX3, unlike LOX4, is under the control of jasmonates [20] and, like LOX2, LOX3 is induced by MJ [64,65]. It is interesting to note that MJ, a 12-OPDA derivative (for the structure, see Figure 5), more actively interacted with LOX2 and LOX3, and that LOX4 and LOX6 were insensitive to MJ. As inferred from the photometric and polarographic activity determinations (Table 3), high substrate concentrations (30 µM) decreased the MJ’s inhibitory action, which is suggestive of a competitive inhibition mode that differs from 12-OPDA. This was confirmed by kinetic studies revealing that, except for LOX3, MJ was identified as a competitive inhibitor. It is of note that high concentrations of substrate eliminated MJ’s activity on LOX3 when probed at inhibitor concentrations of 113 µM. This competitive characteristic was also the case for LOX6 with 12-OPDA (see Figures S8B and S9A), suggestive of a mixed mode of enzyme inhibition [66]. The types of inhibition tentatively assessed herein might be complicated and not uniform [67], but in-depth physical chemistry analysis, as performed in Mogul et al. [67], was beyond the scope of this manuscript. Next to substrate and inhibitor concentration-dependent LOX inhibition, 12-OPDA inhibited in a time-dependent manner, as shown for LOX4 (see Figure S10B). Furthermore, it was demonstrated that 12-OPDA modifies LOX4 cysteinyl thiols and inhibits LOX4, due to its electrophilic character (Figure S13B). The interaction sites and the extent of feedback inhibition by MJ and 12-OPDA on 13-LOX isoforms await clarification.

The co-expression of HPL with LOX2 and LOX3 (see ATTED II (http://atted.jp/) entries, accessed on 22 May 2021) and network association with remaining LOXs (see STRING entries (https://string-db.org/, accessed on 22 May 2021) might relate to additional feedback regulatory mechanisms on oxylipin synthesis, as mentioned in [19]. Our data reveal that TA did not influence the activities of LOX2 and its isoforms. Therefore, feedback inhibition from the HPL branch-derived TA to 13-LOX seems unlikely to occur in vivo.

### 3.6. Cyclopentenone Prostaglandins as Potential LOX Inhibitors

LOX inhibitors were reported to enhance the proteolysis of hLOX into its two domains [43], supporting the predicted docking of 12-OPDA to the inter-domain and the potential involvement of C203 in LOX4 stabilization (Figure 8). Whether 12-OPDA targets C203 or other cysteinyl thiols in PLAT domain proteins should be investigated in future studies to elucidate the specificity of protein activity regulation by 12-OPDA, as demonstrated in this study for At-13-LOXs. Effective 13-LOX, hLOX12 and hLOX5 inhibitors are reported to exhibit IC_50_ values below 5 µM [66], qualifying 12-OPDA as a weak LOX inhibitor. However, the potential interaction of 12-OPDA or cyclopentenones related to 12-OPDA, for example, PGJ_2_ or 15d-PGJ_2_, with proteins of LOX activity, such as the LOX4-related hLOX12 (see Figure 8), provide novel insights and promising perspectives. Together with the extended 12-OPDA and OCPD synthesis protocols provided herein and in the 12-OPDA derivative synthesis protocol [68], these studies are also potential starting points for future investigations.

## 4. Materials and Methods

### 4.1. Data processing, Databases and Computational Studies

Data processing, calculations and presentations were performed using Microsoft Office 2016. Statistical analyses relied on astatsa.com/OneWay_Anova_with_TukeyHSD (2016 Version, CC, Navendu Vasavada, accessed on 28 May 2021). IC_50_ values were determined with the aid of the IC_50_ online calculator (www.aatbio.com/tools/ic50-calculator accessed on 30 May 2021). Protein sequences were retrieved from the Uniprot database (www.uniprot.org/ accessed on 14 May 2021). The prediction of chloroplast sequences, domains and physicochemical parameters of At-LOXs were obtained using ChloroP (http://www.cbs.dtu.dk/services/ChloroP/ accessed on 20 April 2021), NCBI’s “conserved domains” (www.ncbi.nlm.nih.gov/Structure/cdd/wrpsb.cgi accessed on 14 May 2021) and the tools available at the Expasy bioinformatics resource portal (www.expasy.org/ accessed on 20 April 2021). The 3D models of At-LOXs were generated based on soybean 13-LOX (SLOX, PDB 4wfo), and hLOX12 was modeled based on hLOX15 (2p0m.1) by the SWISS MODEL homology-modeling server (swissmodel.expasy.org/ accessed on 14 May 2021) using default settings [69]. The quality of the models was estimated with global model quality estimation (GMQE) and QMEAN values of 0.69 and −2.97 (LOX2), 0.69 and −2.44 (LOX3), 0.76 and −2.31 (LOX4), 0.67 and −3.58 (LOX6) and 0.86 and −2.34 (hLOX12). Active site residues were identified by the alignment of 13-LOXs with LOX3, based on [20]. The finalization and depiction of structures were performed with Pymol (the PyMOL molecular graphics system, version 1.2r3pre, Schrödinger, LLC) or UCSF Chimera [70].

Blind docking was performed with Swiss docking (www.swissdock.ch/docking, accessed on 28 April 2021) [71] and CB-Dock (clab.labshare.cn/cb-dock/php/, accessed on 28 April 2021) [72], with LOX models (see above) and MOL2 files of 12-OPDA generated from Pubchem structural data files (pubchem.ncbi.nlm.nih.gov/ accessed on 28 April 2021) via open source program open Babel [73]. Multiple protein alignment was performed with Clustal Omega (www.ebi.ac.uk/Tools/msa/clustalo/, accessed on 29 April 2021) [74].

### 4.2. Chemicals and Reagents

DHA (99%, 271,551), ARA (≥95.0%, 10,931), LeA (≥99%, L2376), linoleic acid (LA) (≥99%, L1376) and MJ (95%, 392,707) were purchased from Sigma (Darmstadt, Germany). TA was obtained from Tokyo Chemicals (TCI, Tokyo, Japan). NEM, salicylic acid (as sodium salt), buffers, other chemicals and solvents were of the highest analytical grade and were obtained from Sigma, Roth (Karlsruhe, Germany) and Merck (Darmstadt, Germany). Then, 12-OPDA and OCPD were synthesized as described previously [33]. Substrates were prepared according to [75] by mixing equal volumes of 40 mM PUFA stock solution (in EtOH) with 0.1 M KOH and the addition of dH_2_O to obtain a 2.0 mM solution, which was further diluted as indicated.

### 4.3. Subcellular Localization Studies

The LOX2, LOX3, LOX4 and LOX6 encoding cDNAs of putative chloroplast transit peptides (see the respective TAIR (https://www.arabidopsis.org/ accessed on 18 March 2019) entries At3g45140, At1g17420, At1g72520 and At1g67560) were fused to the yellow fluorescent protein (YFP). These were cloned into the 35S-YFP-NosT vector using BamHI and AgeI restriction sites, which were added to the coding sequences by PCR (for primer sequences, see Supplementary Table S1). The protoplast preparation and transfection were performed as described in [76]. The subcellular distribution of the fluorescent protein was examined by confocal laser scanning microscopy (LSM 5 Exciter, Zeiss) with a C-Apochromat 40×/1.2 W autocorr objective. YFP was excited at 488 nm and a 2% line of the argon-ion laser, and the emission was recorded with the BP 505–600-nm filter. The chlorophyll autofluorescence was detected with the LP 650-nm filter and excited at 488 nm. The pinhole was 90 µm and the pixel dwell time was 4.58 µs, with a line average of 4, and images were encoded by 12-bit. Fluorescence images and spectra were analyzed with the ZEN Digital Imaging Software. The magenta color was used to code the chlorophyll fluorescence and green color for YFP.

### 4.4. Cloning, Expression and Preparation of Lipoxygenase Wild-Type and Variant LOX2, LOX3, LOX4, LOX6 and LOX4 C203S

The cDNAs encoding 13-LOX proteins were amplified from the RAFL cDNA clones obtained from the RIKEN Bioresource Center (https://web.brc.riken.jp/en/accessed on 14 May 2018) by PCR, using gene-specific primers (Supplementary Table S1). The cDNAs were cloned into the Invitrogen pET15b vector (Merck, Darmstadt, Germany) or Invitrogen pEXP5-NT-TOPO vector (Fisher, Schwerte, Germany; LOX6) in frame with the N-terminal His-tag and transformed into Nico21(DE3) cells (NEB) for heterologous expression in *E. coli*. LOX4C203S was generated with LOX4 as a template and in vitro mutagenesis primers (Supplementary Table S1). The correctness of the constructs was verified by DNA sequencing. Then, 600 mL Luria–Bertani medium, containing 600 µg/mL ampicillin, was inoculated with 50 mL of a non-induced overnight bacteria culture and incubated at 37 °C and 150 rpm to an OD_600_ of 0.7–0.8. Protein expression was then induced by the addition of isopropyl-β-D-thiogalactopyranoside (IPTG) to a final concentration of 0.35 mM and 0.1 mM of NH_4_Fe-(III)-citrate. Induced cultures were grown at 25 °C, with orbital shaking at 120 rpm for 20 h. The cultures were centrifuged at 6000 rpm for 30 min at 4 °C and the harvested cell pellets were stored at −80 °C.

For protein purification, 4 mL of ice-cold lysis buffer (50 mM Tris-HCl, pH 8.0, 5.0 mM imidazole, 10% glycerol, 50 mM NaCl, 0.05 mM PMSF, 0.1 mM pepstatin, 0.1 mM pefablock, 0.25 mM EDTA and 2.5 mM ß-mercaptoethanol) was added per gram of cell pellet. Cells were lysed by ultrasonication (30 s for each g of cell pellet, with 20 s pause intervals on ice; Sonicator Bandelin HD 2070, amplitude 70%). The lysate was centrifuged (25 min, 10,000 rpm, 4 °C) and the resulting supernatant (L) was incubated with Ni^2+^-NTA (equilibrated with lysis buffer) for 30 min at 4 °C. The washing (50 mM Tris-HCl, pH 8.0, 10 mM imidazole, 10% glycerol, 50 mM NaCl) and elution (50 mM Tris-HCl, pH 8.0, 250 mM imidazole, 10% glycerol, 50 mM NaCl) of proteins was performed according to the manufacturer’s instructions (Roth, Karlsruhe, Germany, His/Ni NTA-HP Beads (0805)) at 4 °C. The purification of LOX2 via Ni^2+^-NTA was unsuccessful. After overnight dialysis of eluates or L at 4 °C against a 1000-fold volume of buffer (50 mM Tris-HCl, pH 8.0) enzymes, denoted as eluates (E) and cleared lysates (Cl), were frozen in liquid N_2_ and stored at −80 °C. Enzyme quality was checked by the dilution of proteins with Laemmli buffer without a reducing agent, heating (95 °C, 5 min), and separation on 7.5% SDS-PAGE, followed by Coomassie blue staining. Protein amounts were determined with a Bio-Rad assay standardized with bovine serum albumin.

### 4.5. Lipoxygenase Activity

The LOX-catalyzed hydroperoxidation of PUFAs was assayed using two different methods. Firstly, oxygen electrode measurements were performed at 25 °C (Oxygraph + PC-operated oxygen electrode control unit with USB 2.0 connectivity, Hansatech, Norfolk UK) with a stirring speed of 50 rpm. The electrode was calibrated as described in the manufacturer’s manual. Air-saturated substrate medium (1.8 mL, 245 ± 15 nmol O_2_ mL^−1^) was equilibrated to a constant baseline for 5 min prior to injection of the sample via a Hamilton syringe. The LOX-initiated oxygen consumption was monitored as a function of time, and the linear slope after enzyme injection was used for activity calculations. No oxygen consumption occurred when the enzyme was omitted from the injection medium. Secondly, in the spectrophotometric approach, the LOX-catalyzed epoxide formation was monitored at 234 nm (ε = 25,000 L·mol^−1^·cm^−1^; [77]) (Shimadzu 2401 spectrophotometer) at 25 °C. Activities were calculated from the initial linear rates. All buffers were saturated with air. Kinetic parameters were determined by adding fixed LOX amounts to 6–10 different PUFA concentrations and plotting the LOX activity (nmol hydroperoxide·s^−1^·mg^−1^) against substrate concentration, applying the Michaelis–Menten equation using the Microsoft Excel-Solver. All LOX incubates and buffers supplemented with metal salts were checked for unchanged pH values in comparison to control or salt-free buffers.

The regiospecificity of LOX-mediated DHA and ARA oxygenation was determined using the method described in [33] with minor modifications. Then, 13-LOX-expressing cell sediment (3 g) was re-suspended in 4 mL 50 mM Tris-HCl, pH 8.0, and then sonicated (2 × 30 s, 1 × 45 s). Then, 50 mM Tris-HCl, pH 8.0 (4 mL), 26 µL PUFA and 25 µL EtOH were added to the supernatant obtained by centrifugation (4 °C, 5500 rpm, 25 min). After incubation for 25 min on ice, lipid extraction was performed as described in [33]. Pooled CHCl_3_ phases were evaporated under inert gas (N_2_). The residue was dissolved in 360 µL MeOH and filtrated (0.22 µm) prior to loading on RP-HPLC (Dionex HPLC system) with the following parameters and settings: HPLC guard column, VP10/8 Nucleodur C18 Isis (Machery and Nagel, Düren, Germany); HPLC main pillar, VP250/10 Nucleodur C18 Isis, 5 µm (Machery and Nagel, Düren, Germany); Solution A (A), 80% MeOH, 19.9% Millipore Water (MPW), 0.1% acetic acid; Solution B (B), 99.9% MeOH, 0.1% acetic acid, column temperature 30 °C, flow rate 4.166 mL/minute. After column equilibration for 10 min with A, the run was started by the injection of 10 µL of the synthesis mix. After 9 min, B was increased from 0% to 25% within 5 min and, during the following 2 min, B was increased to 100%. The UV spectrum of the eluate was monitored at 224 nm (Chromeleon, Version 6.60, Dionex; Thermo Fisher, Waltham, MA, USA).

As for the DHA-LOX product analysis, major peaks fractionating at an elution time of ~13 min were collected and analyzed with a Q-IMS-TOF mass spectrometer Synapt G2Si (Waters GmbH, Manchester, UK) operated in resolution mode and interfaced to a nano-ESI ion source. N_2_ served both as the nebulizer gas and the dry gas. N_2_ was generated with the nitrogen generator NGM 11. Argon served as the collision gas for MS^2^ experiments by CID (collision-induced dissociation). Samples from the LC-separation were introduced by static nano-ESI using in-house pulled glass emitters. The mass axis was externally calibrated with fragment ions of Glu-1-fibrinopeptide B as the calibration standard. In MS experiments, the protonated signal of leucine-enkephalin was used as an internal mass standard. Scan accumulation and data processing was performed with MassLynx 4.1 (Waters GmbH, Manchester, UK) on a PC workstation. The spectra were generated by accumulating and averaging 50 single spectra. Determinations of the exact masses were performed using centroided data.

ARA-LOX2 product analysis was performed by the comparison of retention times with 15-hydroperoxyarachidonic acid (15-HPETE) standard, prepared by incubation of ARA with SLOX (Sigma-Aldrich, Taufkirchen, Germany, L7395) as described in [32].

### 4.6. Effect of pH and Various Compounds on LOX Activities

The pH profiles of LOX activities were determined spectrophotometrically with 9.6 µM LeA, dissolved in K-P_i_, pH 6.6, or Tris-HCl, pH 7.2–8.6. The effect of chemicals on LOX activity was determined using both techniques: (i) with an O_2_-electrode by injecting preincubated (5 min at 35 °C and 5 min at 25 °C) enzyme to 30 µM LeA (in 50 mM Tris-HCl, pH 7.2); and (ii) with a spectrophotometer by incubating 13.5 µL (0.25 µg/µL) 13-LOX with 1.5 µL 2.8 mM chemical stocks or solvent (10 min, 30 °C) and the injection of 3 µL incubate into 123 µL LeA (4.8 µM in 50 mM Tris-HCl, pH 7.2). Inhibitory studies were performed spectrophotometrically by the pre-incubation of 15 µL enzymes (0.25 µg/µL) with 2 µL chemical stocks (120–2880 µM) or solvent (0 µM) for 10 min at 30 °C, and the successive injection of incubate (3 µL) into 123 µL 4.8 µM LeA (in 50 mM Tris-HCl, pH 7.2). This approach was also used for kinetic inhibition studies, using four different substrate concentrations for the determination of inhibition type and inhibitor constant (Ki)-values, with Michaelis–Menten, Lineweaver–Burk and Dixon plots.

The effect of 12-OPDA on LOX4 at two enzyme concentrations in the presence of GSH was analyzed by preincubating (25 °C, 5 min) 1.5 µL 40 mM GSH with 45 µL LOX4 (0.27 or 0.54 µg/µL) and further incubation (10 min, 25 °C) with 5 µL 12-OPDA (1.4 mM in EtOH) or EtOH (control). The effect of doubling the GSH concentration was studied as described, using a stock of 80 mM and a LOX4 concentration of 0.54 µg/µL. The influence of the 12-OPDA-GSH adduct on LOX4 activity was studied by adding 45 µL (0.54 µg/µL) LOX4 to 5 µL of the 12-OPDA-GSH mixture in 40 mM K-P_i_, pH 8.0 (see below). The control was performed by adding 5 µL GSH mixture excluding 12-OPDA to 45 µL (0.54 µg/µL), while the 12-OPDA-LOX4 sample was obtained by adding 5 µL 1.4 mM 12-OPDA (in 50% 40 mM K-P_i_, pH 8.0) to 45 µL (0.54 µg/µL) LOX4 and incubating at 25 °C for 10 min. All prepared samples were injected into 20 µM LeA, and oxygen consumption was recorded as described. Then, 12-OPDA-GSH adduct was prepared by incubating (35 °C, 6 h) 2.8 mM 12-OPDA (in EtOH) with an equal volume of 35 mM GSH (in 40 mM K-P_i_ pH, 8.0).

### 4.7. Effect of Divalent Cations on LOX-Catalyzed LeA Dioxygenation

The dependence of LOX activity on CaCl_2_, MgCl_2_, CdSO_4_ and CuCl_2_ dissolved in 20 mM HEPES, pH 8.0, was determined O_2_-polarographically [78]. Briefly, 55.0 µL of protein (0.5 µg/µL) was injected into an O_2_-electrode cuvette filled with 30 µM LeA (±1.0 mM salts). For CuCl_2_, 100 µM was used, due to the strong metal-catalyzed oxidation of LeA. The Cd^2+^ effect was studied spectrophotometrically, using 4 µg LOX2 at indicated concentrations, preincubated for 10 min at 25 °C. The residual LOX activity was measured by injecting 8 µL incubate (0.2 µg/µL) into 110 µL LeA (9 µM in 20 mM HEPES, pH 8.0). The effect of metals on LOX was studied with *E. coli*-expressed protein pellets dissolved in TRIS-HCl, pH 8.0. The protocol followed the details outlined for the determination of the regiospecificity of LOX-mediated DHA and ARA oxygenation (Section 4.5) and used synthesized 12-OPDA and OCPD as described in Section 4.8.

### 4.8. LOX-Mediated Synthesis of 12-OPDA, DHA-Peroxide, ARA-Peroxide and OCPD

Synthesis of 12-OPDA and OCPD with 13-LOX-, AOS- and AOC-lysates was performed as described in [33], here expanded to the testing of LOX2, LOX3 and LOX4. The RP-HPLC analysis of oxylipin extracts was performed as described (see Section 4.5) using an injection volume of 20 µL. Successful formation of 12-OPDA and OCPD was confirmed by comparing the elution times with standards as synthesized in [33]. The time course of LA, ARA and LeA peroxide formation by LOX2, and consumption by AOS, was demonstrated spectrophotometrically at 234 nm [77] by adding 3 µg LOX2 to 12 µM PUFA (in 50 mM Tris-HCl, pH 7.2), followed by the addition of 1.5 µg AOS in a total volume of 129 µL.

### 4.9. Determination of Iron Content and Free Cysteines

The iron content was analyzed as described [79]. Free protein thiols were determined as described in [80] with slight variations. Briefly, 150 µL LOX4 (4.2 µM) was mixed with 1.5 µL 30 mM 12-OPDA (in EtOH) or EtOH (solvent control). After 20 min of incubation at 25 °C, samples were mixed with 10 µL dithionitrobencene (DTNB, 2 mM in 40 mM K-P_i_, pH 8.0) and incubated for 30 min in the dark. Samples were measured at 412 nm, ε_412nm_ = 13,600 L^−1^·mol^−1^·cm^−1^ (KC4 microplate reader, Biotec, 25 °C) for determination of the thiol/protein ratio with background correction (150 µL 40 mM K-P_i_, pH 8.0, 10 µL DTNB 2 mM, 1.5 µL ligand stock or EtOH).

### 4.10. Ex Vivo Evaluation of 12-OPDA as a LOX Inhibitor

To evaluate 12-OPDA as a LOX inhibitor under native conditions, we employed pea roots as a plant LOX source. The reliability was assessed with the criterion that an increase in A_234nm_ and, ideally, oxygen consumption occurred via the injection of plant tissue extract (0.5–2 µg/µL protein in 50 mM Tris-HCl or K-P_i_, pH 6.6–8.0) in LeA (tested at 4–100 µM). Furthermore, boiling of plant tissue extract (5 min, 95 °C) resulted in decreased activities, determined as described in the following. Pea seeds (*Pisum sativum* “Kleine Rheinländerin”) were placed in darkness for 4 days on moist paper. The emerged roots were cut, frozen in liquid N_2_, and stored at −80 °C. Frozen tissue (~50 mg/mL) was pulverized in a pre-chilled mortar and immediately ground with 50 mM Tris-HCl, pH 7.2. After centrifugation (14,000 rpm, 4 °C), the clear supernatant was used as a protein source (pea protein).

The immunological detection of 13-LOX was performed, based on [81]. Pea proteins (8 µg) were separated via 7.5% SDS-PAGE and subsequently transferred (30 min, 2.0 mA/cm^2^) with Towbin transfer buffer onto nitrocellulose membrane, with semi-dry blotter. After 1 h blocking with blocking solution (1% (*w*/*v*) non-fat dry milk in TBST (150 mM NaCl, 50 mM Tris-HCl, pH 7.5, 0.05% tween 20)) and overnight incubation (4 °C) with LOX2 (At3g45140) serum (Agrisera, AS07 258, 1:25,000 in blocking solution), the blot was washed with TBST and incubated (2 h, 25 °C) with horseradish peroxidase-coupled anti-rabbit IgG as second antibody (1:20,000 in blocking solution) for the subsequent luminescence detection of immunodecorated LOX on X-ray films (30 s incubation). The effect of 12-OPDA on pea root LOX activity was studied by incubating 60 µL (0.6 µg/µL protein) with 2 µL 30 mM 12-OPDA or EtOH (control) for 45 min at 35 °C. The mixture was injected in 1.8 mL of 20 µM LeA (in 50 mM Tris-HCl, pH 7.2) and recorded for oxygen consumption.

## 5. Conclusions

This report provides novel insights into the regulatory effects of physiologically relevant compounds on 13-LOX isoforms from *A. thaliana*. We confirmed that all 13-LOXs carry chloroplast signal peptides and are potential sources of 12-OPDA. Our hypothesis of LOX inhibition by 12-OPDA was challenged via two methods, revealing that among recombinantly expressed proteins, LOX3 and LOX4 were the most sensitive to inhibition by 12-OPDA. Thus, feedback modulation of these oxygenases by 12-OPDA might be relevant in vivo, to decrease the potential accumulation of potentially toxic LOX products under conditions of stress. In addition, we found that LOX3 and LOX2 were most sensitive toward inhibition by MJ. Insight into selective interactions is also provided for divalent metals; of which, the highly toxic cadmium metal ion activated LOX2 and inactivated LOX3, LOX4 and LOX6. Furthermore, we suggest similarities between *A. thaliana* 13-LOXs and human LOXs, known to be involved in inflammation and diseases.

## Figures and Tables

**Table 1:** Substrate specificities of LOX2, LOX3, LOX4 and LOX6.

PUFA	Relative Activity (%)			
	LOX2	LOX3	LOX4	LOX6
LeA	100	100	100	100
LA	79 ± 7	12 ± 1	11 ± 1	17 ± 1
ARA	50 ± 4	5 ± 1	4 ± 1	8 ± 3
DHA	56 ± 3	7 ± 1	8 ± 1	31 ± 3

Activities were determined spectrophotometrically at a wavelength of 234 nm, employing 4 µg 13-LOX and 31 µM PUFA in 50 mM Tris-HCl, pH 7.2, in a total volume of 128 µL. Here, 100% refers to the activities (nmol·s^−1^·mg^−1^) obtained with LeA, LOX2: 4.4 ± 0.3, LOX3: 71 ± 2, LOX4: 118 ± 2, LOX6: 2.3 ± 0.2. All values are means ± SD of n ≥ 3.

**Table 2:** Effect of divalent metals on 13-LOX activity.

Metal Salts	Relative Activity (%)			
	LOX2	LOX3	LOX4	LOX6
CaCl_2_ (1 mM)	690 ± 60% *	117 ± 10% *	100 ± 4%	121 ± 15% *
MgCl_2_ (1 mM)	170 ± 18% *	135 ± 4% *	106 ± 3% *	142 ± 15% *
CdSO_4_ (1 mM)	220 ± 18% *	no activity	no activity	no activity
CuCl_2_ (100 µM)	50 ± 20% *	no activity	no activity	no activity

Enzymes (55 µL of 0.5 µg/µL LOX2, LOX3, LOX4 or 100 µL of 1.0 µg/µL LOX6) were injected into LeA (30 µM in 20 mM HEPES pH 8.0) ± 1 mM MgCl_2_ or CaCl_2_, CuCl_2_ (100 µM) and CdSO_4_ (1 mM). The control values of oxygen consumption in the absence of metal substitutions (nmol·mL^−1^·min^−1^) were as follows. LOX2: 2.3 ± 0.3, LOX3: 13.8 ± 1.0, LOX4: 20.0 ±1.0, LOX6: 1.8 ± 0.8. All values are means ± SD of n ≥ 3. Asterisks denote a significant difference (*p* < 0.05) in comparison to the control (post hoc Tukey HSD).

**Table 3:** The effect of various chemicals on 13-LOX activity.

**Polarography**	**Relative Activity (%)**			
	**LOX2**	**LOX3**	**LOX4**	**LOX6**
NEM	84 ± 4% *	51 ± 6% *	11 ± 3% *	47 ± 15% *
12-OPDA	64 ± 4% *	60 ± 5% *	21 ± 5% *	50 ± 10% *
MJ	87 ± 10% *	85 ± 5% *	95 ± 3%	94 ± 6%
TA	95 ± 8%	100 ± 5%	100 ± 9%	101 ± 12%
SA	96 ± 7%	101 ± 5%	101 ± 4%	103 ± 5%
**Photometry**	**Relative activity (%)**			
	**LOX2**	**LOX3**	**LOX4**	**LOX6**
NEM	35 ± 10% *	29 ± 10% *	3 ± 2% *	13 ± 2% *
12-OPDA	49 ± 10% *	35 ± 5% *	6 ± 4% *	59 ± 10% *
MJ	62 ± 10% *	60 ± 4% *	99 ± 7%	86 ± 6% *
TA	105 ± 5%	103 ± 4%	106 ± 4%	100 ± 9%
SA	100 ± 8%	106 ± 5%	103 ± 8%	100 ± 9%

Upper table, polarographic recordings. LOX6: 90 µL (1.0 µg/µL) was incubated with 10 µL 2.8 mM chemicals or solvent, LOX2, LOX3: LOX4: 45 µL (0.5 µg/µL) were incubated with 5 µL 2.8 mM chemicals or solvent and injected into 1.8 mL LeA (30 µM in 50 mM Tris-HCl, pH 7.2). Values of oxygen consumption (nmol·mL^−1^·min^−1^) are means (± SD of n ≥ 3), and the control values were: LOX2: 3.6 ± 0.6, LOX3: 14.6 ± 1.0, LOX4: 18.6 ± 3.0, LOX6: 2.8 ± 0.7. Lower table, photometric recordings. Enzymes (0.225 µg/µL) were incubated (10 min at 30 °C) with 280 µM chemicals or solvent (0 µM, control) and injected (3 µL) into 123 µL LeA (4.8 µM in 50 mM Tris-HCl, pH 7.2). Here, 100% refers to the control activity. Values of conjugated diene formation were measured at 234 nm (nmol·s^−1^·mg^−1^) and are means (n ≥ 3 ± SD). Control values: LOX2: 1.7 ± 0.2, LOX3: 33.1 ± 2.0, LOX4: 37.0 ± 2.0, LOX6: 2.5 ± 0.3. Asterisks denote a significant difference (*p* < 0.05) in comparison to the control (post hoc Tukey HSD).

## Data Availability

All necessary data are contained in this paper. Upon request, additional data can be sent.

## Supplementary Materials

Supplementary materials can be found at https://www.mdpi.com/article/10.3390/ijms221910237/s1.
