# Supplementary material for: Biochemical Characterization of 13-Lipoxygenases of Arabidopsis thaliana
Source: Int J Mol Sci. 2021 Sep 23;22(19):10237. doi: 10.3390/ijms221910237 (PMC8508710; doi:10.3390/ijms221910237)
Supplement: Supplementary file 1 [file ijms-22-10237-s001.zip › ijms-1305611-supplementary-Figures.pdf]

International Journal of
Molecular Sciences

MDPI

Supplementary Materials to

Biochemical characterization of chloroplast 13-lipoxygenases of *Arabidopsis thaliana by* Daniel Maynard[1], Kamel Chibani[1], Sonja Schmidtpott[1], Thorsten Seidel [1], Jens Sproß [2], Andrea Viehhauser[1], Karl-Josef Dietz[1§]

Table of contents:

.	page
**Table S1.** Oligonucleotides and cDNA sources used in this study	2
**Table S2.** Confirmation of the sum formulas of HPDH (C22H32O4) and its ω-6 fragment ion (C17H22O3+Na+).	3
**Table S3.** Metal ion effects on 13-LOXs under conditions employed for 12-OPDA synthesis.	4
**Figure S1** Heterologous expressed 13-LOX are functional enzymes	5
**Figure S2.** SDS-PAGE analysis, pH-profiles and size exclusion separations of 13-LOXs.	6
**Figure S3.** Enzymatic analysis of LOX2 with LA as substrate.	7
**Figure S4.** 13-LOX and AOS mediated conversion of PUFAs and PUFA peroxides demonstrated for LOX2.	8
**Figure S5.** Positional analysis of 13-LOX-generated DHA-peroxide (HPDH).	9
**Figure S6.** Residual activities of 13-LOXs pre-incubated with LA and LeA and LOX2 activation by Cd^{2+}.	10
**Figure S7.** Interaction and activity studies of LOX2, LOX3 and LOX6	11
**Figure S8.** Michaelis Menten and Lineweaver-Burk plots depicting the MJ- and 12-OPDA-effect on LOX2 activity and the 12-OPDA effect on LOX6.	12
**Figure S9.** Michaelis Menten and Lineweaver-Burk plots depicting the effect of MJ and 12-OPDA on LOX3.	13
**Figure S10.** Michaelis Menten, Lineweaver-Burk and time dependent enzyme inhibition study characterizing the effect of 12-OPDA on LOX4 activity at high substrate concentration.	14
**Figure S11.** 13-LOX inactivation curves.	15
**Figure S12.** Ex vivo studies on the inhibitory effects of 12-OPDA on LOX in pea protein extract.	16
**Figure S13.** LOX4 activities in presence of various 12-OPDA and GSH concentrations.	17
**Word file S1.** Clustal omega sequence analysis of selected lipoxygenases.	18

Table S1. Oligonucleotides and cDNA sources used in this study.

construct	template	primer pairs (5´-3´)	expression vector
LOX2$_{71\text{-}896}$	RAFL09-06-022	F:CCC GCT AGC ATG GCA CAG AAT ATT AAA GTA<br>R:CCC CGG ATC CTC AAA TAG AAA TAC TAT A	pet15b
LOX3$_{71\text{-}919}$	RAFL09-85-I10	F: CCC CAT ATG GCA GAC AGA AAG AGC GAA<br>R: CCC CCT CGA GTT ATA TAG ATA CAC TAT T	pet15b
LOX4$_{71\text{-}926}$	RAFL08-10-015	F:CCC CCA TAT GGC AAT TAG CAC TGT CGG TAG A<br>R:CCC CGG ATC CCT AAA TAG ATA CAC TAT T	pet15b
LOX6$_{41\text{-}917}$	RAFL04-16-H12	F: GCT GTG ATC AGC CGT GAA G<br>R: CCC CGG ATC CCG AAC CTG TCT GGA TAT AGG	pEXP5NT/Topo
LOX4 C203S	LOX4	F: GAG GGT TTC GCA TCT GGC CCT GTT CAC TTC CCC<br>R: GGG GAA GTG AAC AGG GCC AGA TGC GAA ACC CTC	pet15b
LOX2$_{1\text{-}55}$	Col-0-RNA	BamF: aaaaggatcca ATGTATTGTA GAGAGTCC<br>AgeR: ttttaccggtCGTGAAGCGGTGACCTT	35S-YFP-NosT
LOX3$_{1\text{-}51}$	Col-0-RNA	BamF: aaaaggatcca ATGGCCTTAG CTAAAGAG<br>AgeR: ttttaccggtACGACGCCGGATTTGGT	35S-YFP-NosT
LOX4$_{1\text{-}58}$	Col-0-RNA	BamF: aaaaggatcca ATGGCTTTAG CTAATGAG<br>AgeR: ttttaccggTACAACGCCGCTTGAGTT	35S-YFP-NosT
LOX6$_{1\text{-}40}$	Col-0-RNA	BamF: aaaaggatcca ATGTTCGTAG CATCTCCG<br>AgeR: ttttaccggTCGAACCTGTCTGGATAT	35S-YFP-NosT

Table S2. Confirmation of the sum formulas of HPDH ($C_{22}H_{32}O_4$) and its ω-6 fragment ion ($C_{17}H_{22}O_3$+Na^+), synthesized by LOX2, LOX3, and LOX4, respectively.

	**HPDH+Na^+**	**Δmass (ppm)**	**ω-6 fragment of HPDH+Na^+**	**Δmass (ppm)**
	m_{Theo} 383.2193	n.a.	m_{Theo} 297.1461	n.a.
**LOX2**	m_{Exp} 383.2184	2.30	m_{Exp} 297.1465	1.28
**LOX3**	m_{Exp} 383.2205	3.18	m_{Exp} 297.1468	2.29
**LOX4**	m_{Exp} 383.2189	0.99	m_{Exp} 297.1471	3.30

Table S3. **Metal ion effects on 13-LOXs under conditions employed for 12-OPDA synthesis.** Proteins were expressed in *E. coli* and lysed in 50 mM TRIS buffer, pH 8.0. After centrifugation, supernatants were diluted 1:10 resulting in protein concentrations of 0.6-0.7 mg/ml and injected into 50 mM HEPES, pH 8.0, supplemented with 1mM metal salts and subsequent addition of 6 µL 0.2 mM LeA. Absorptions at 234nm were recorded for at least 300 sec and initial oxidation rates were used for calculation. Activities relate to control values obtained with 50 mM HEPES, pH 8.0, and are means±SD of three measurements. $CuCl_2$ was not tested due to its inactivating properties on 13-LOXs. $CdCl_2$ time-dependently inactivated LOX3, LOX4 and LOX6 (not shown). Details see main text.

**Metal ions**	**Relative activity [%]**			
	**LOX2**	**LOX3**	**LOX4**	**LOX6**
$CaCl_2$ [1 mM]	550±40%	104±2%	85±6%	107±11%
$MgCl_2$ [1 mM]	188±28%	108±4%	107±2%	123±10%
$CdSO_4$ [1 mM]	950±240%	63±3%	40±6%	40±10%

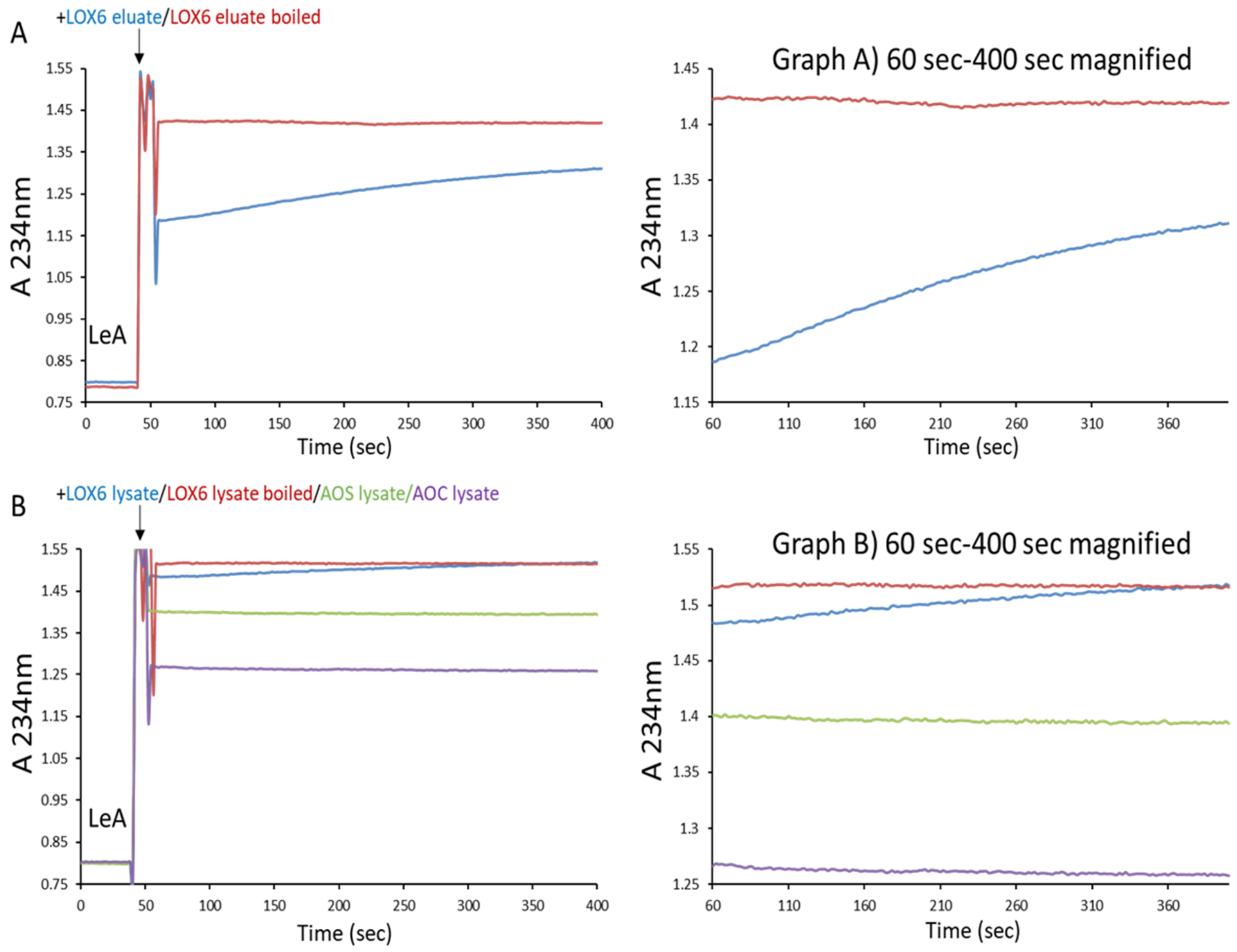

Figure S1 Heterologous expressed 13-LOX are functional enzymes. As shown for LOX6, 13-LOX are active proteins, that specifically oxidize PUFA, as shown by comparison of LOX6 eluate and LOX6 lysate with lysates of AOS, AOC, and boiled LOX6 eluate and -lysate (30 sec 85°C) incubated with LeA. The assay was conducted as follows. 10 uL of 0.2mM LeA was mixed with 100 µL TRIS, pH 8.0, 50 mM and after 40 sec, 2 µL proteins (LOX6 eluate (see A), LOX6 lysate (see B), AOS&AOC lysate (see B)) or boiled proteins (LOX6 eluate (see A), LOX6 lysate (see B)) of 1.1 µg/µL protein content were added. The absorption was recorded at 234 nm for a time interval of 400 sec at 25°C. Measurements are representative of multiple runs.

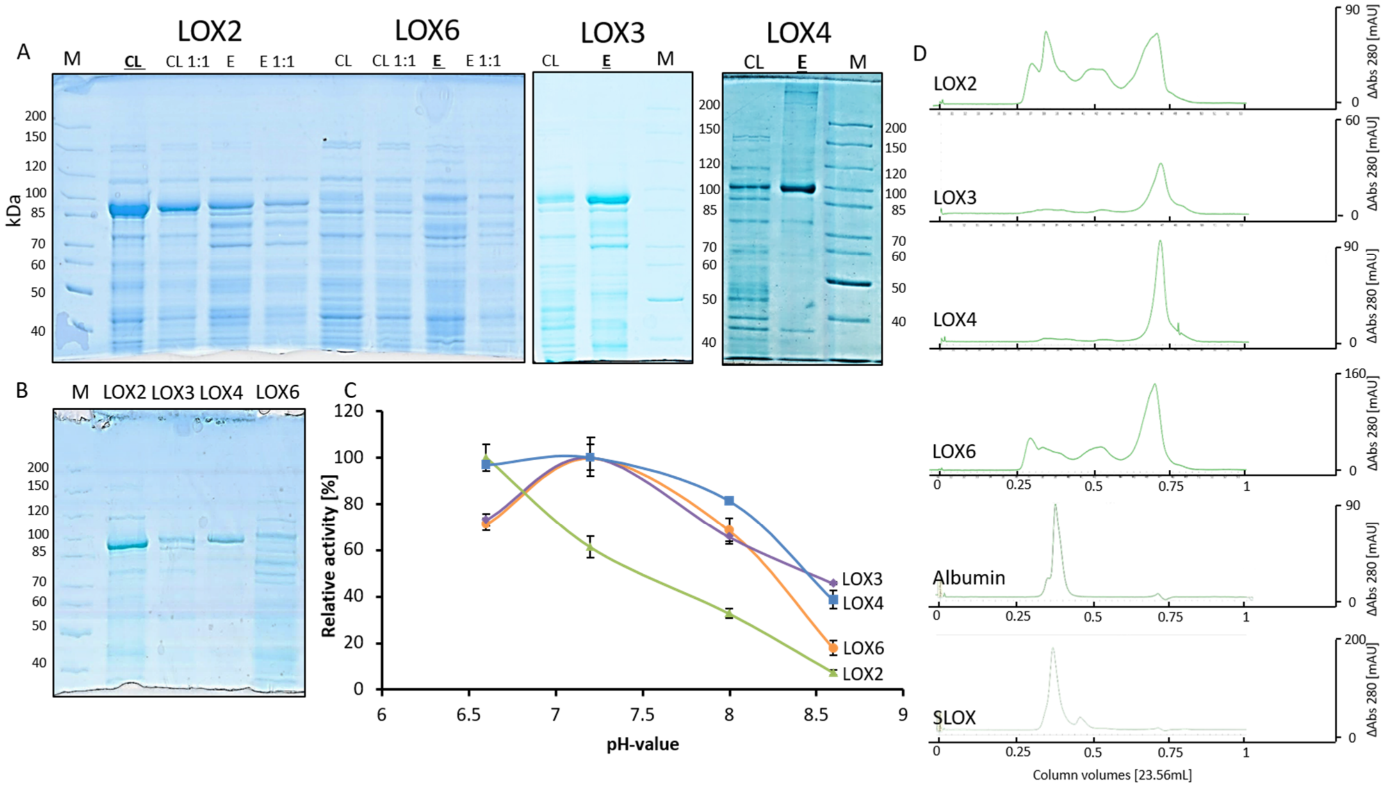

[Figure S2.](#) **SDS-PAGE analysis, pH-profiles and size exclusion separations of 13-LOXs. (A)** SDS PAGE dilution analysis of LOX2 and LOX6 cleared lysates (CL) and Ni^{2+}-NTA eluates (E). 8 µg and 4 µg protein/lane as indicated were analyzed on 7.5% SDS-PAGE and stained with Coomassie Brilliant Blue. Below, analysis (4 µg protein/lane) of LOX3 and LOX4 CL and eluates. M indicates the molecular mass marker. Each protein fraction contained 13-LOX that migrated to the expected mass. LOX3, LOX4 and LOX6 eluates (E) were utilized for characterization, as eluates were liberated from protein impurities in comparison to CL unlike for LOX2, here CL were used. (**B**) Combined SDS-PAGE of 13-LOX proteins utilized herein. 3 µg protein/lane of LOX3-LOX6 (E) and LOX2 (Cl) were loaded on 7.5% SDS-PAGE and stained as in A. M indicates the molecular mass marker. Further details see Materials and Methods. (**C**) 13-LOX activity pH profiles. Assays were performed with its preferred substrate LeA (9.8 µM in 50 mM K-Pi, pH 6.6 and 50 mM Tris-HCl, pH 7.2-8.6). Enzymatic activities relate to conjugated diene formation as determined spectrophotometrically at 234 nm. All data relate to maximum activity (LOX2: 3.9±0.4, LOX3: 50.5±2.8, LOX4: 62.3±0.4, LOX6: 1.6±0.1 nmol $s^{-1}.mg^{-1}$) normalized to 100% and are expressed as mean ± SD of n≥3. **D**) Gel-chromatographic analysis of 13-LOXs. Proteins of 1 mg/ml (LOX3) or-2 mg/ml (LOX2, 4 and 6) were loaded on TRIS 50 mM, pH 8.0, NaCl 100mM (running buffer) equilibrated column ENrich™ SEC 650 10 x 300 Column (Biorad), at a flowrate of 0.5 ml per min in combination with an FPLC application (NGC, BioRad). Absorbance at 280 nm was monitored with the integrated UV- detector. Albumin 66.5 kDa (1 mg/ml) and SLOX ~100 kDa (2 mg/ml) (Sigma Aldrich, Taufkirchen, Germany, L7395) were separated as well to compare elution profiles, revealing the unexpected elution patterns of 13-LOXs. Every run was followed by washing with two column volumes running buffer.

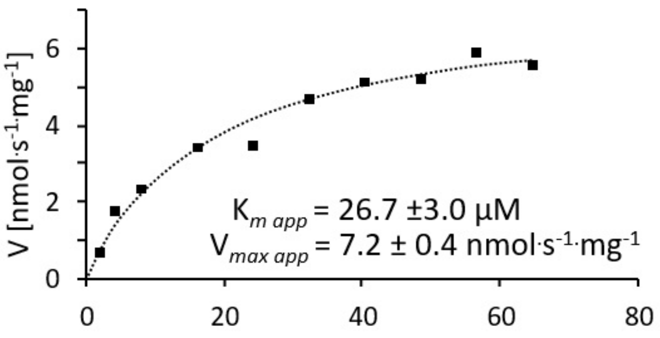

Figure S3. **Enzymatic analysis of LOX2 with LA as substrate.** Graph is representative for at least three determinations yielding highly similar kinetic parameters, shown as mean±SD derived from nonlinear regression fit using Michaelis Menten equation.

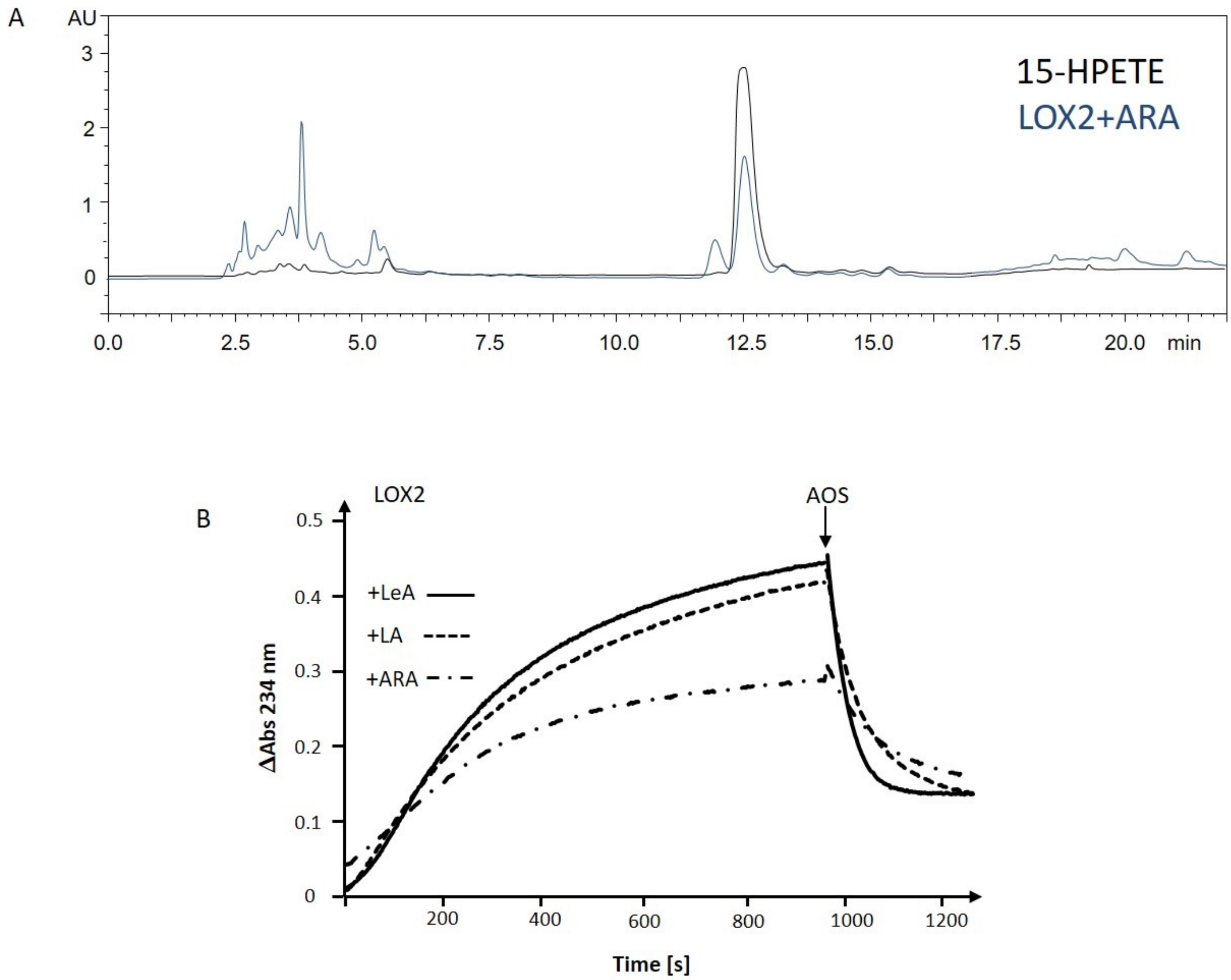

Figure S4. 13-LOX and AOS mediated conversion of PUFAs and PUFA peroxides demonstrated for LOX2. (**A**) Positional analysis of LOX2-generated ARA-peroxide. RP-HPLC analysis of oxylipins extracted from LOX2-ARA incubate (blue graph) and RP-HPLC analysis of 15-HPETE standard (black graph). (**B**) Photometric demonstration of LeA, LA and ARA peroxidation and successive allene oxide synthesis by monitoring LOX2-mediated increase and AOS-mediated decrease of absorbance at 234 nm as described in Material and Methods.

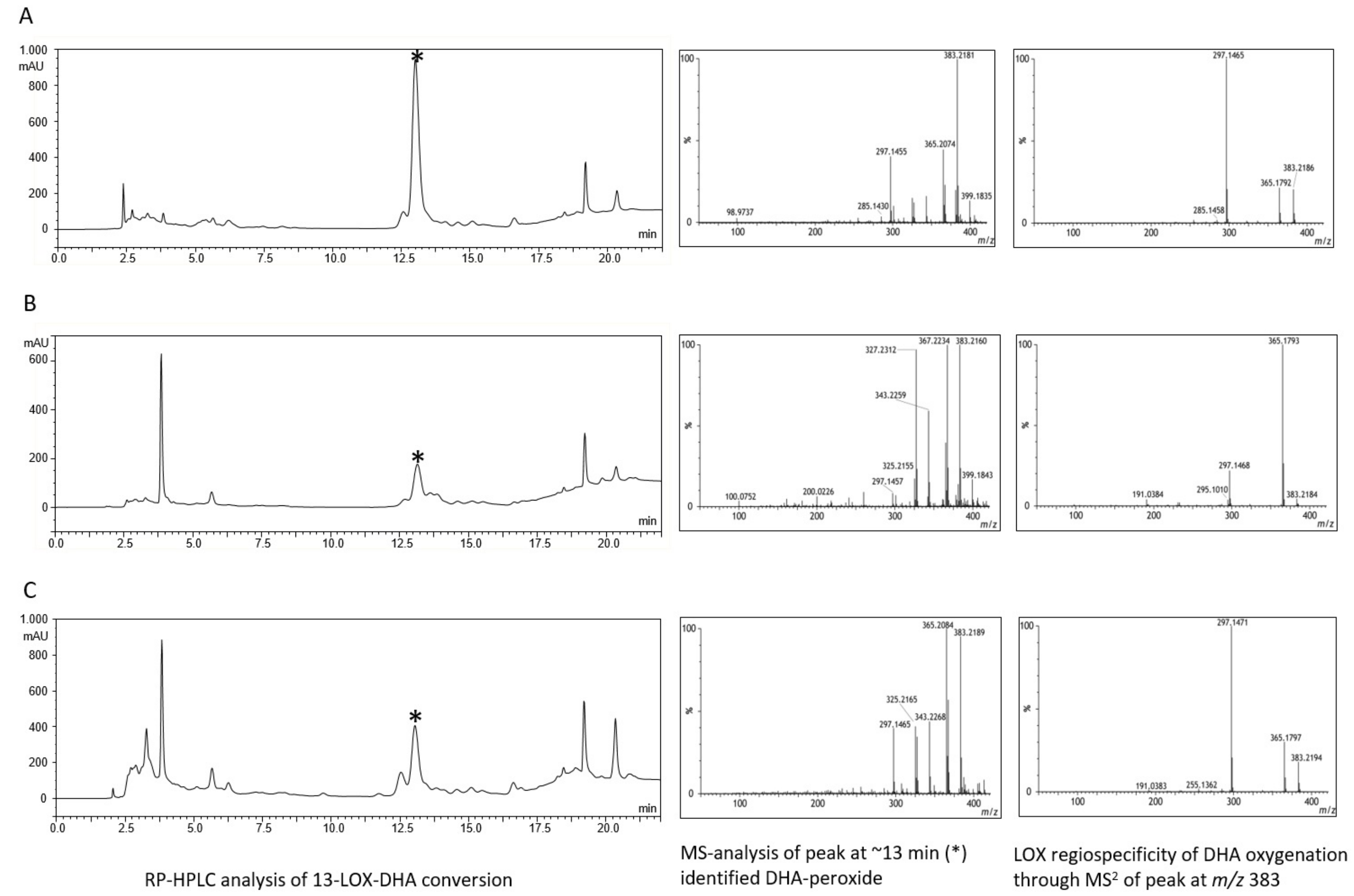

Figure S5. Positional analysis of 13-LOX-generated DHA-peroxide (HPDH). Lysates of LOX2, LOX3 and LOX4, with a protein amount of ~20 mg/mL were incubated with DHA and oxygenation products were extracted using Bligh Dyer Method as described in Material and Methods. Fractions eluting at ~13 min indicated by asterisks were analyzed via MS and MS^2 revealing that LOX2 (**A**), LOX3 (**B**) and LOX4 (**C**) synthesized HPDH, as indicated by the major signal appearing at *m/z* 383.2 due to association (HPDH, M.W. 360.5) with sodium $[M+Na]^+$. The LOX specificity towards DHA oxygenation was gained by MS^2 analysis of the molecular ion (*m/z* 383.2). The mass fragmented into an ion of *m/z* 365.2 due to loss of water $[M+Na-H_2O]^+$and the fragment ion appearing at *m/z* 297.1 indicated that the mass loss was due to a fragment with the mass of 86 Da ($CH_3CH_2CHCHCH_2$-OH). The appearance of the latter fragment confirmed the position of hydroperoxidation at ω-6 [33]. This hypothesis is further endorsed by the confirmation of the sum formulas of the molecular ion and the *m/z* 297.2 fragment ion using accurate mass (Table S1).

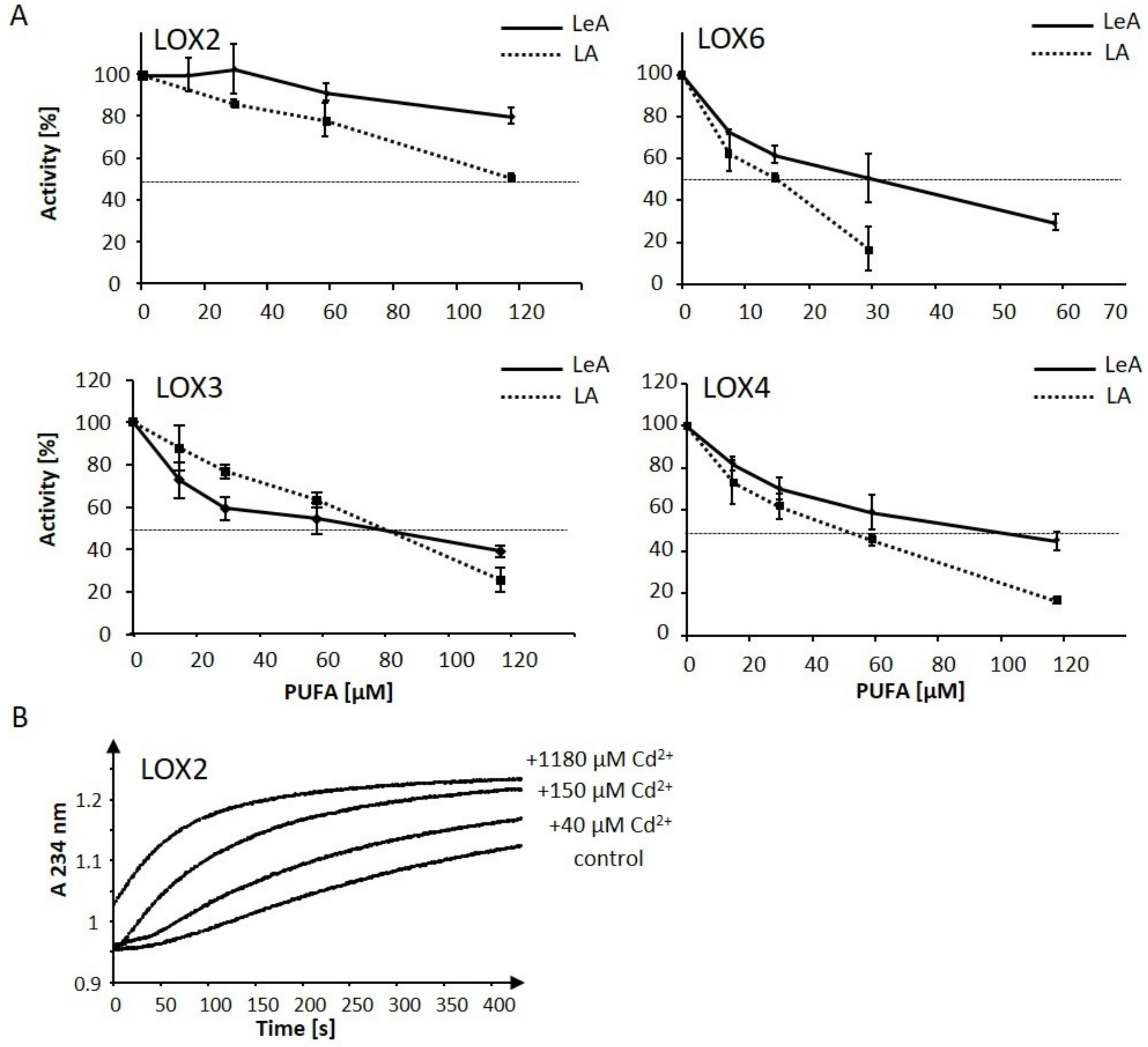

Figure S6. Residual activities of 13-LOXs pre-incubated with LA and LeA and LOX2 activation by Cd^{2+}. (**A**) LOXs (0.22 µg/µL) were incubated with PUFAs at indicated concentrations for 10 min at 30°C and residual LOX activity was measured spectrophotometrically by injection of 3 µL incubation mixture into 123 µL LeA (4.8 µM in 50 mM Tris-HCl, pH 7.2). Values (nmol sec^{-1} mg^{-1}, means ± SD (n≥2)) are given relative to LOX activity obtained with solvent (control, set at 100%). Control values, LOX2: 1.6±0.4, LOX3: 30±2, LOX4: 40±4, LOX6: 2.4±0.4. (**B**) LOX2 was incubated with Cd^{2+} or its solvent dH_2O (control) at indicated concentrations and residual LOX activity (1.6 µg) was measured spectrophotometrically with 20 mM HEPES, pH 8.0, and 9 µM LeA. Activities (nmol sec^{-1} mg^{-1}), mean±SD (n≥3): control (0 µM Cd^{2+}): 0.8±0.2, 40 µM Cd^{2+}: 2.3±0.3, 150 µM Cd^{2+}: 5.2±0.9, 1180 µM Cd^{2+}: 5.7±0.7. No change of A234 nm occurred when LOX2 was omitted from the cuvette filled with indicated LeA and final Cd^{2+} concentration.

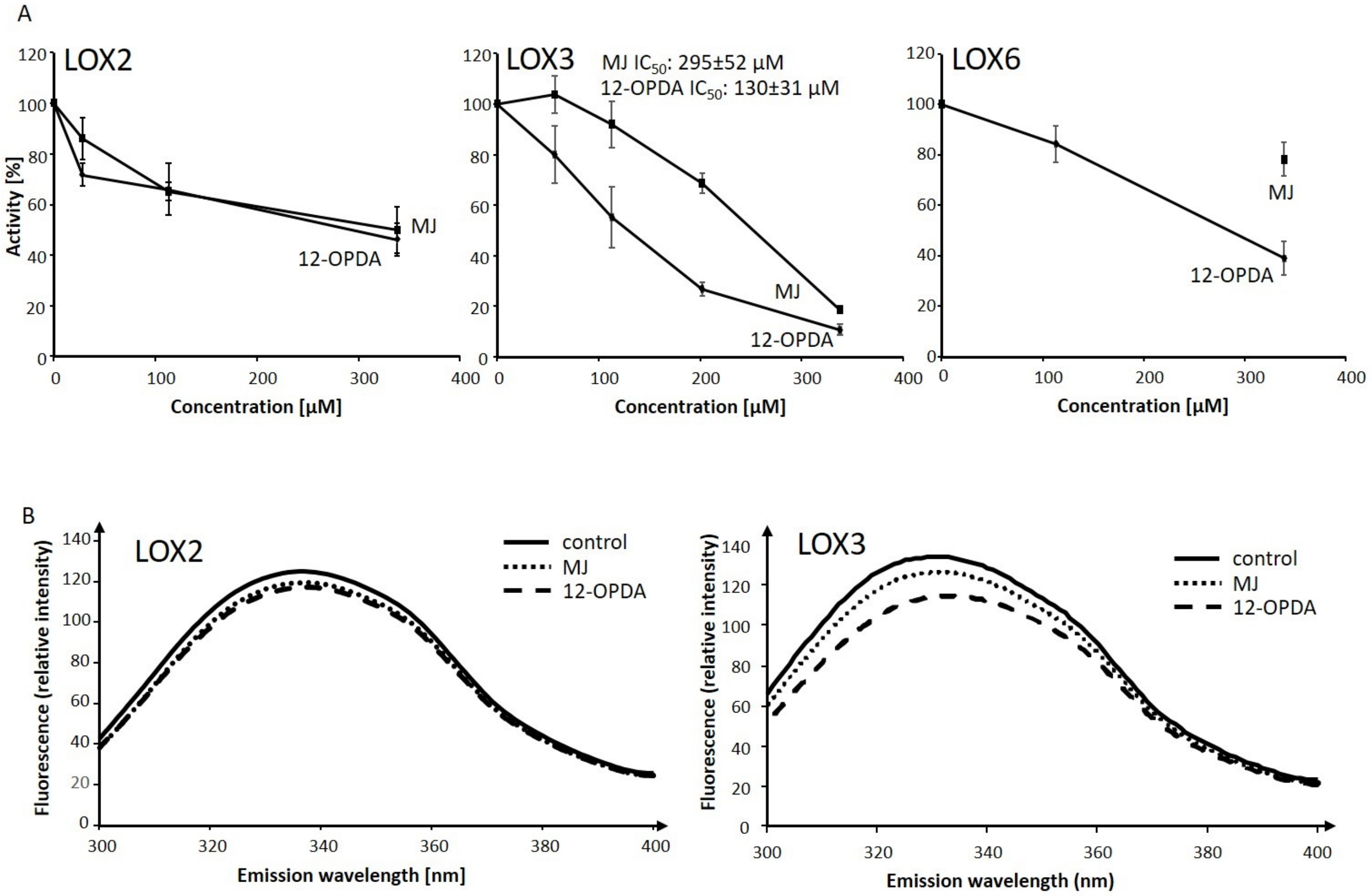

Figure S7. Interaction and activity studies of LOX2, LOX3 and LOX6. (**A**) LOX2, LOX3 and LOX6 activities in presence of increasing amounts of MJ and 12-OPDA. Analysis was performed as described for IC_{50} determination of LOX4 (see Figure 5A). 100% activity (0 µM, control) in $nmol{\cdot}s^{-1}{\cdot}mg^{-1}$ corresponded to 2.0±0.2 (LOX2), 37±3 (LOX3) and 2.0±0.1 (LOX6). (**B**) Intrinsic fluorescence of LOX2 and LOX3 incubated with 12-OPDA, MJ and solvent control. Analysis was performed as in Figure 5B. LOX2 values (n≥3±SD) of relative fluorescence intensities at respective emission maxima (control: 336.5±0.6, 12-OPDA, 337.2±0.3, MJ 337.3±0.6) were as follows. 125.5±2.0 (control), 117.5±2.0 (12-OPDA), 119.7±2.7 (MJ). LOX3 values (n≥3±SD) of relative fluorescence intensities at respective emission maxima (control: 330.8±1.6, 12-OPDA, 333.2±0.3, MJ 331.7±0.8) were as follows. 134.5±3.9 (control), 115.2±1.3 (12-OPDA), 126.4±3.0 (MJ).

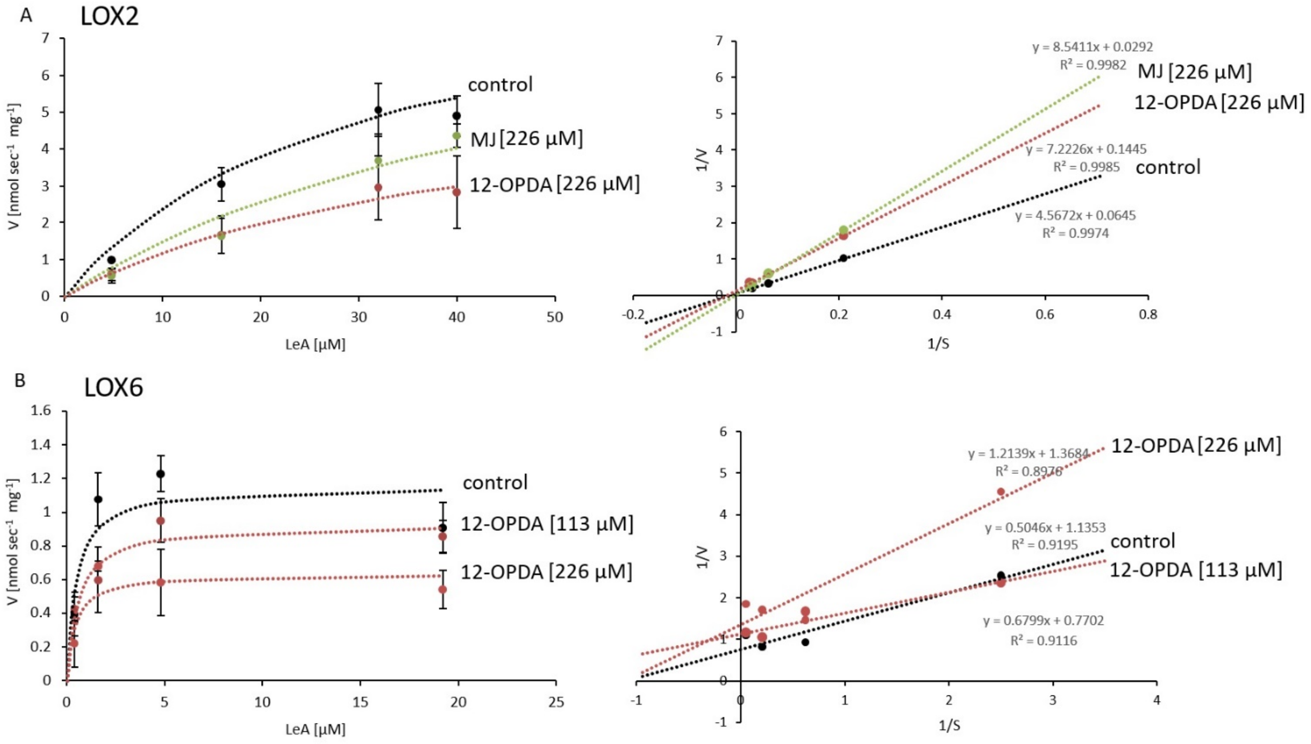

Figure S8. Michaelis Menten and Lineweaver-Burk plots depicting the MJ- and 12-OPDA-effect on LOX2 activity and the 12-OPDA effect on LOX6. Black symbols and fitted curves or straight lines represent enzyme activity in the absence of inhibitor, while colored lines represent different concentrations of inhibitor. Analysis of pre-incubated proteins (0.22 mg/ml) at 30°C (control, solvent of 12-OPDA or MJ) was performed as decribed in Material and Methods. Values represent means±SD of at least three measurements.

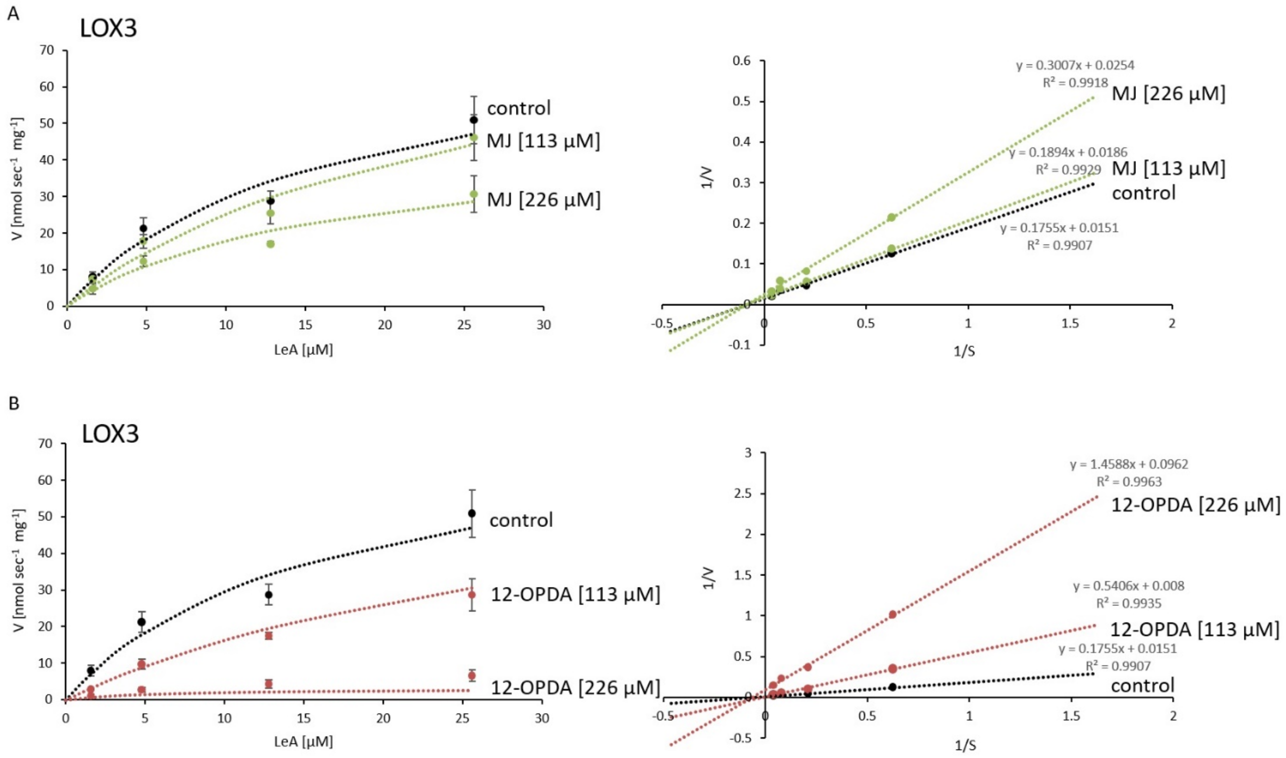

Figure S9. **Michaelis Menten and Lineweaver-Burk plots depicting the effect of MJ and 12-OPDA on LOX3.** Black symbols and fitted curves or straight lines represent enzyme activity in the absence of inhibitor, while colored lines represent different concentrations of inhibitor. Analysis of pre-incubated protein (0.22 mg/ml) at 30°C (control, solvent of 12-OPDA or MJ) was performed as decribed in Material and Methods. Values represent means±SD of at least three measurements.

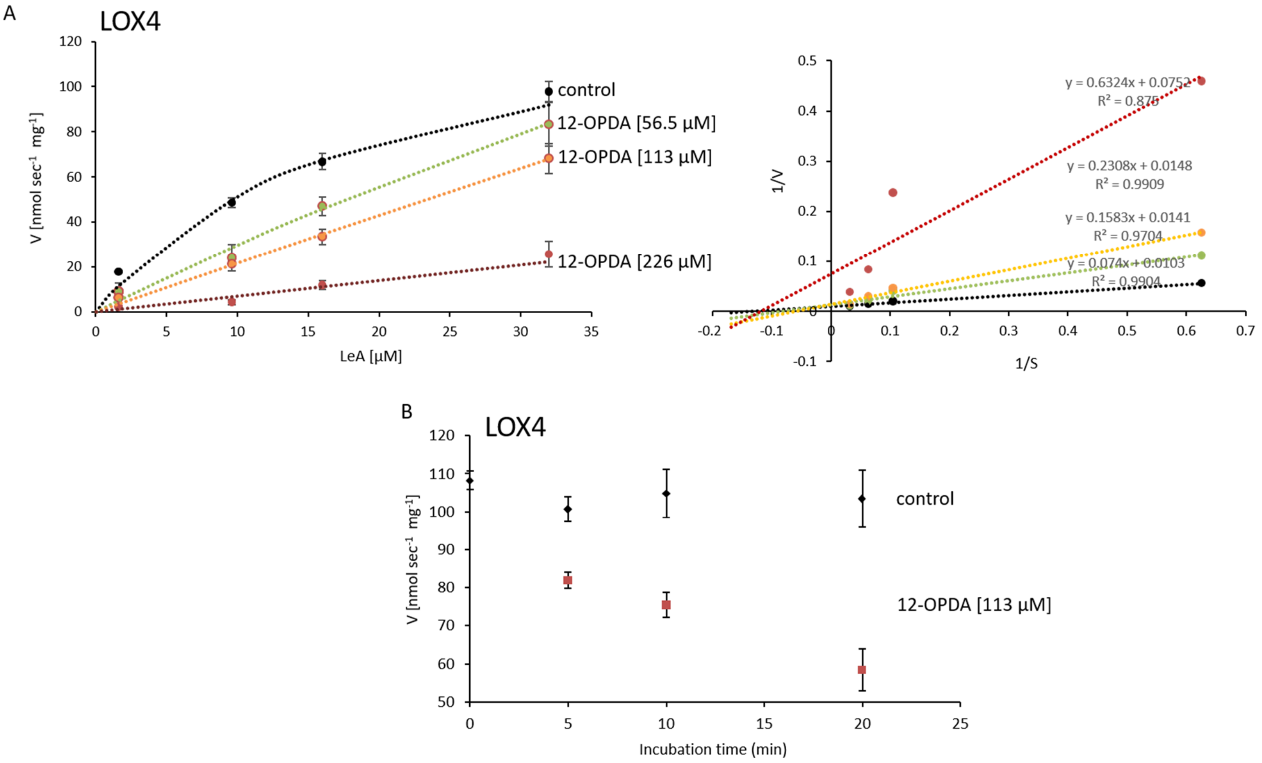

Figure S10. Michaelis Menten, Lineweaver-Burk and time dependent enzyme inhibition study characterizing the effect of 12-OPDA on LOX4 activity at high substrate concentration. A) Black symbols and fitted curves or straight lines represent enzyme activity in the absence of inhibitor, while colored lines represent different concentrations of inhibitor. Values represent means±SD of at least three measurements. Analysis of pre-incubated protein (0.22 mg/ml), at 30°C (control, solvent of 12-OPDA), was performed as decribed in Material and Methods. **B)** LOX4 (0.22 mg/ml) was preincubated with 12-OPDA or solvent (control) at 30°C for indicated time points and analysed for remaining activity with 32 µM LeA (means ±SD, n=3).

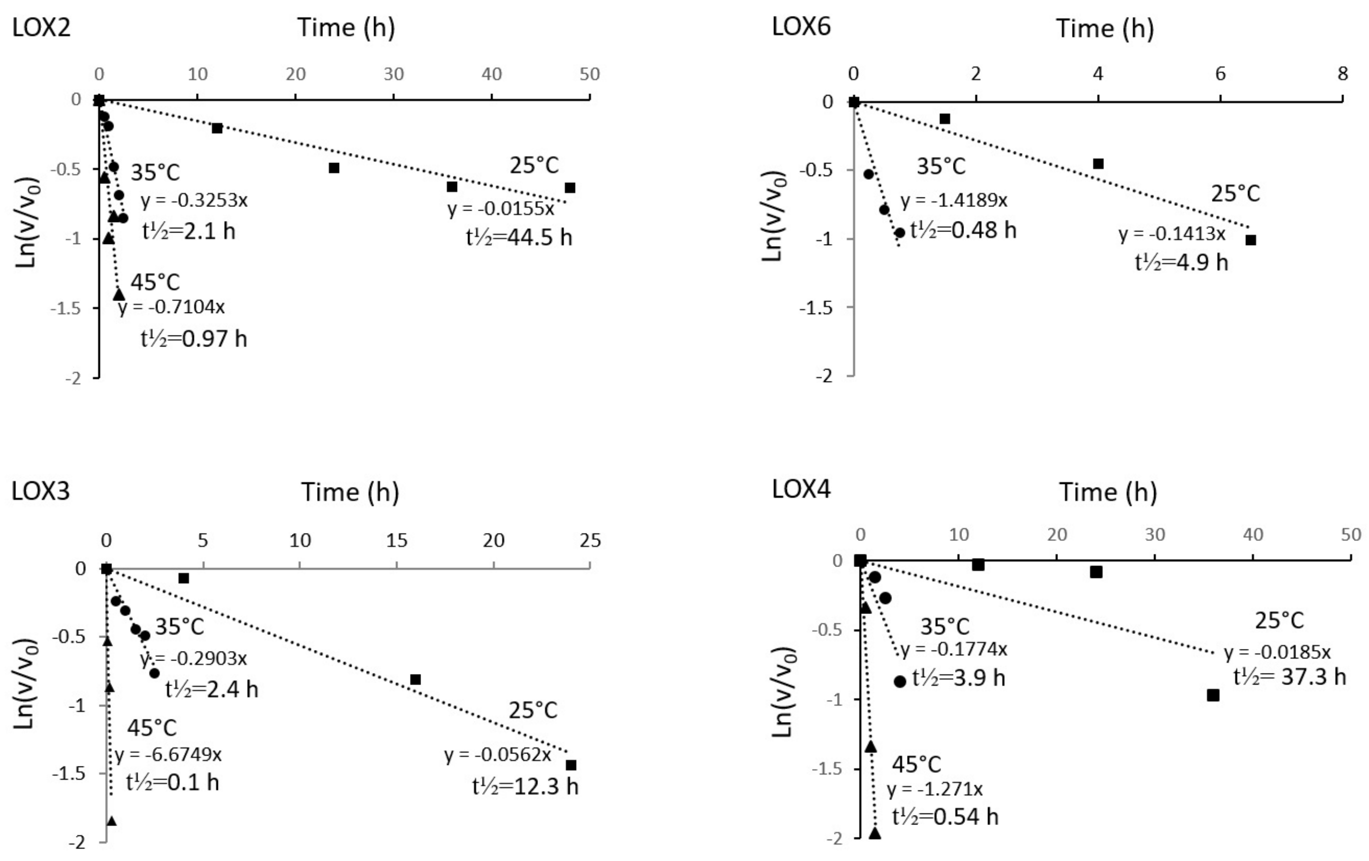

Figure S11. 13-LOX inactivation curves. Half-lives are derived from first order kinetic equation obtained by semi-log plot of residual activity (1 mg/ml proteins) vs. incubation time at indicated temperature. The regression coefficients for linear fits were >0.9 except for LOX4 at 25°C (0.62) and 35°C (0.8). At 45°C, LOX6 was inactivated within 15 min of incubation time.

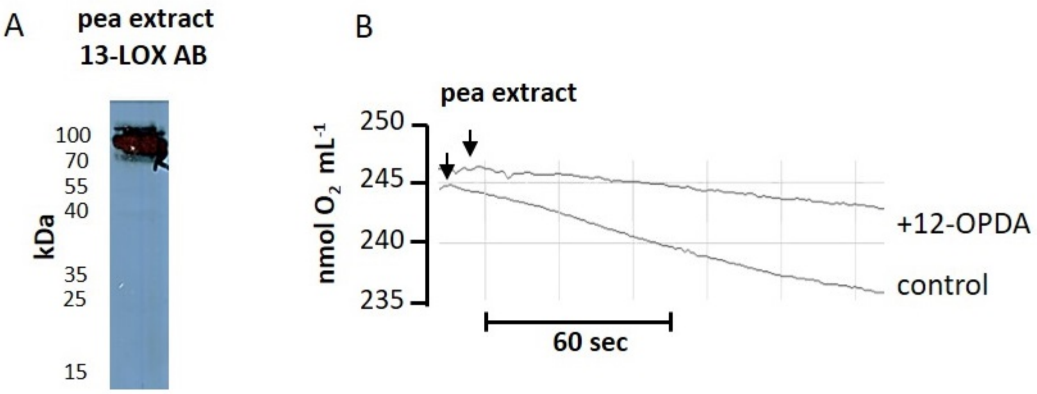

Figure S12. ***Ex vivo*** **studies on the inhibitory effects of 12-OPDA on LOX in pea protein extract.** (**A**) Immunological identification of LOX in pea root extract utilized for LOX activity detection in presence of 12-OPDA. (**B**) Polarographic analysis reveal LOX inhibition by 12-OPDA under *ex vivo* conditions.

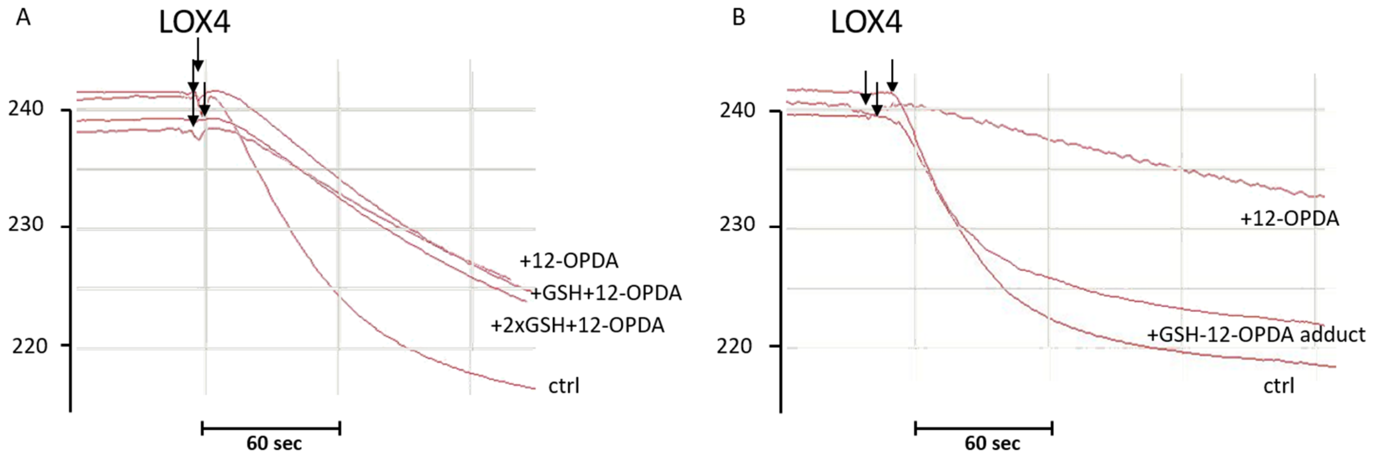

Figure S13. LOX4 activities in presence of various 12-OPDA and GSH concentrations. In (**A**) the assay consisted of 24 µg LOX4 (control), 24 µg LOX4+140 µM 12-OPDA (+12-OPDA), 24 µg LOX4+140 µM 12-OPDA+1.3 mM GSH (+GSH-12-OPDA), 24 µg LOX4+140 µM 12-OPDA+2.6 mM GSH (2xGSH+12-OPDA). In (**B**) 24 µg LOX4 was incubated with 140 µM 12-OPDA (+12-OPDA) and 140 µM 12-OPDA that was reacted with GSH for 6 h to allow formation of 12-OPDA-GSH (+GSH-12-OPDA adduct). Arrows denote addition of incubates into 1.8 mL 20 µM LeA. Shown are recordings representative of n≥2.
