# Supplementary material for: Biochemical Characterization of 13-Lipoxygenases of Arabidopsis thaliana
Source: Int J Mol Sci. 2021 Sep 23;22(19):10237. doi: 10.3390/ijms221910237 (PMC8508710; doi:10.3390/ijms221910237)
Supplement: Supplementary file 1 [file ijms-22-10237-s001.zip › ijms-1305611-supplementary-File S1.pdf]

```
|A0A4D6T4S4|AaeLOX|      ------------------------------------------------------------    0
|A0A3G6V563|LiLOX|       --------------------------MELGLGA----RQAVLQPAA--------------    16
|P93184|HvLOX2.1|        ------------------MLT----------------ATKPLV---------GGACAAPS    17
|Q8GSM3|HvLOX2.2|        ------------------MQT----------------ATKPLV---------GARAVP--    15
|P38419|OsLOX7|          ------------------MLRP-------QLNPSSHTTTTS--SSSSTQLFASSSCIA--    31
|P38418|LOX2|            ------------------MYC----------RESLSSLQTLNVAKSLSSLFPK----Q--    26
|Q8GV01|BnLOX2|          ------------------MFC----------KEASSSLQTLNIAKSLSSEFTK----P--    26
|Q8GSM2|HvLOX2.3|        --------------------MI-------HLKQP-----LVL-SAQSSN-V-ASPLFV--    23
|A1XCI5|ZmLOX10|         ------------------MMNL-------NLKQP-----LVL-PAHHSN-V-VGSRLS--    25
|A1XCI6|ZmLOX11|         ------------------MIQM-------NMKQP-----LVL-PAHRGN-V-IGSRLA--    25
|E3NYV3|OeLOX2|          ------------------MLNL-------SIS-KSQTHQILL-PNCNPFF--FGRRNA--    29
|Q6X5R6|NaLOX2|          --------------------KP-------QLHQTSESKKTLI-PWSATKSFTSGEILA--    30
|Q96573|SlLOXc|          ------------------MLKP-------QFQQ---STKTLI-PSWNT----NTLFLA--    25
|O24370|StLOX2|          ------------------MLKP-------QLQQSSQSTKALI-PSWNT----NPLFLA--    28
|P08170|SLOX|            ------------------------------------------------------------    0
|P14856|PsLOX2|          ------------------------------------------------------------    0
|Q9CAG3|LOX6|            -------------------------------MF---------VASPVKTNFNGVS-----    15
|A0A176VER0|MpLOX|       ------------------MVSLRGPAVDLSMAHISRAPASAISPNSTSILF--PR-----    35
|A1XCI4|ZmLOX9|          ------------------MAAPCRDLTGLRRGL---AAA----------RL-APG-----    23
|Q9LNR3|LOX3|            -------------------MALAKELMGYPLIT---ERSSLVSS-----ASHFKK-----    28
|Q9FNX8|LOX4|            -------------------MALANEIMGSRLIF---ERSSSLA-SPFHSRFSIKK-----    32
|A0A078ILA7|BnLOX|       -------------------MALAKEIMGSRLIF---ERSSSLASSPFQSRLSIKK-----    33
|E3NYV2|OeLOX1|          -------------------MALTKEIMGFSLMQ---KSSFLGS-SNF------LV-----    26
|Q6X5R5|NaLOX3|          -------------------MALAKEIMGISLVE---KSSVIS---SSKVFLNPNF-----    30
|Q96574|SlLOXd|          -------------------MALAKEIMGISLLE---KS-------SSMALLNPNN-----    26
|O24371|StLOX3|          -------------------MALAKEIMGISLLE---KSSSFMN-SSSMALFNPNN-----    32
|A0A1Q4S425|CyLOX|       ------------------------------------------------------------    0
|O16025|PhAOSLOX|        FSRHENEKRPDDYLRKEYVERLQKGPVNYRLQIQIHE----ASPDDTATIFHAGILWD--    294
|P18054|hLOX12|          ------------------------------------------------------------    0
|P16050|hLOX15|          ------------------------------------------------------------    0
|P09917|hLOX5|           ------------------------------------------------------------    0
|O15296|hLOX15B|         ------------------------------------------------------------    0


|A0A4D6T4S4|AaeLOX|      ------------------------------------------------------------    0
|A0A3G6V563|LiLOX|       -ASRA---PLGLLKAG---HRAQ----------QRRLPSVPSVSASASVTQGI--PQTSF    57
|P93184|HvLOX2.1|        SSARRRTFVVP----E--ARRKP--------GNGRRTSVS---KVGSTSTSTTTTTTTTL    60
|Q8GSM3|HvLOX2.2|        -LSRRASFLVA----E--ARRKP--------STNARRT-----RVGSTST---TTTTTTI    52
|P38419|OsLOX7|          -SLRRPSSSSSSVVAA--ARRTR----------GQGSSRV---VVVCASSSAT-------    68
|P38418|LOX2|            -S-----ALINPISAG---RRN----NLPRPNLRRRCKV----TASRANIEQE-------    62
|Q8GV01|BnLOX2|          -S-----ALVNPLSAG---HRYK---LFPRPNLRGRCTV----TTSKSDIDWI-------    63
|Q8GSM2|HvLOX2.3|        -A-----G--------GQQRRAS---GAGRTCSGRRLSAR---RISCASTEEA-------    56
|A1XCI5|ZmLOX10|         -S-----SSPS----AAAASRRTGGGVSSRSGSRRHVRLP---RISCSATEEV-------    65
|A1XCI6|ZmLOX11|         -S-----SPSPVAAAAAAASRRRGVVSSPRSRLQRHMRLP---RISCSATEEA-------    69
|E3NYV3|OeLOX2|          -S-----FAGNPKFKS--VRKHENVRV------GRGSSTI---KAVQTSAEKS-------    65
|Q6X5R6|NaLOX2|          -S-----FPINILSKNIRQNPKKNFRVH-----YYAANST---KAVLSSTEKS-------    69
|Q96573|SlLOXc|          -S-----FPINILNKNFILKKKNNFRVHH-N--YNGANTI---KAVLNSTQKS-------    66
|O24370|StLOX2|          -S-----FPINILNKNFRLKKKNNFRVHH-N--YNGASTT---KAVLSSTEKA-------    69
|P08170|SLOX|            ------------------------------------------------------------    0
|P14856|PsLOX2|          ------------------------------------------------------------    0
|Q9CAG3|LOX6|            -LVKSPAFSALSCRKQ-------------HR--VPISRQV---RAVISREEKA-VDQED-    54
|A0A176VER0|MpLOX|       -AGA-----LKSLGKG---SPAPAAAVSR------KFGSV---RATLSNVRPT-NQTSNF    76
|A1XCI4|ZmLOX9|          -RRGANKLCLAPFHHR---APPPQQQQGRRR--QRQGLKV---VAAIS--EDL-PRLAA-    70
|Q9LNR3|LOX3|            -RTQSTQFSINP-FDR-----------RPRK--T--KSGV---VAAIS--EDL-VKTLRF    65
|Q9FNX8|LOX4|            -KTQRTQFSINP-FDP-----------RPMR--AVNSSGV---VAAIS--EDL-VKTLRI    71
|A0A078ILA7|BnLOX|       -KTQRTQVSINP-FDL-----------SPMK--AAISGGV---VAAIS--EDL-VKTLRF    72
|E3NYV2|OeLOX1|          -YRKHNQFCFNTVLVP---AKRK--RFQEKR--ASKVPTL---VAAISDKLDL-V-----    69
|Q6X5R5|NaLOX3|          -YQKENQLCVNRQFQG---RRN----LRTRR--VLR-QSP---MAAI--SENL-I-----    68
|Q96574|SlLOXd|          -YHKENHLWFNQQFQG---RRN----LSRRK--AYR-QST---MAAI--SENL-V-----    64
|O24371|StLOX3|          -YHKENHLWFNQQFQG---RRN----LSRRK--AFR-QST---MAAI--SENL-I-----    70
|A0A1Q4S425|CyLOX|       ------------------------------------------------------------    0
|O16025|PhAOSLOX|        -KETHPWFDLAK--VS------------------IKTPLSPD----------V-LEKTAF    322
|P18054|hLOX12|          ------------------------------------------------------------    0
|P16050|hLOX15|          ------------------------------------------------------------    0
|P09917|hLOX5|           ------------------------------------------------------------    0
|O15296|hLOX15B|         ------------------------------------------------------------    0


|A0A4D6T4S4|AaeLOX|      ------------------------------------------------------------    0
|A0A3G6V563|LiLOX|       LQKARD---------SVLPHGAP--FTPTSKDSVQWTTTIYTT-QL--------------    91
|P93184|HvLOX2.1|        SADSNGAAVGTV---------TR--PDVHV-QDRTHATEMKATVTVHMS-----------    97
|Q8GSM3|HvLOX2.2|        LTDVNGPALTTV---------AK--PGHQ--YDLKQTVEMKATVSVHMK-----------    88
|P38419|OsLOX7|          --ASRGDSSSDM-------------AAAAA-VRVKAVATIKVTVGELI------------    100
|P38418|LOX2|            ---GNTVK-----------------EPIQN-IKVKGYIT---AQEEFLE-----------    87
|Q8GV01|BnLOX2|          ---A---K-----------------DNAKK-IKVKGIIT---AKQGLLP-----------    85
|Q8GSM2|HvLOX2.3|        ---V-GVS-----------------TSVTT-KERALTVTAI-VTAQVPT-----------    82
|A1XCI5|ZmLOX10|         ---S-GAV-----------------SSVTV-E-RMLTVTASVEASPAIG-----------    91
|A1XCI6|ZmLOX11|         ---S-GAV-----------------SAVTV-E-KMLTVTASVQAAPAIG-----------    95
|E3NYV3|OeLOX2|          ---T-TTT-----------------TS------ATVVITVQQTVGGALT-----------    87
|Q6X5R6|NaLOX2|          -------------------------TS------VKAVVTVQKTVGGT-------------    85
|Q96573|SlLOXc|          -------------------------IG------VKAVVTVQKQV----------------    79
|O24370|StLOX2|          -------------------------TG------VKAVVTVQKQV----------------    82
|P08170|SLOX|            -------------------------MFSAG-HKIKGTVVLMPKNELEVN-----------    23
|P14856|PsLOX2|          ----------------MFPNVTG--LLNKG-HKIRGTVVLMRKNVLDFNTIVSIGGGNVH    41
|Q9CAG3|LOX6|            ---GKKSTNKPLINSSQFPWQRS--KYTGS-KTVTAVVKIRKKIKEKLT-----------    97
|A0A176VER0|MpLOX|       ESGPNGSSGRPSSR----VXSNF--DGSTM-VSLQAWLTIRKKRRPDRD-----------    118
|A1XCI4|ZmLOX9|          ---PGTGKGAPE----------G--RRPEK-VLVRAALTVRRKHKEDLK-----------    103
|Q9LNR3|LOX3|            STTTGDRKSEEE----------E--KAAVK-FKVRAVVTVRNKNKEDLK-----------    101
|Q9FNX8|LOX4|            STVGRKQEKEEE----------E--EKSVK-FKVRAVATVRNKNKEDFK-----------    107
|A0A078ILA7|BnLOX|       KTLGRNQENE-E----------V--EKAVQ-FKVRAVVTVRNKNKEDFK-----------    107
|E3NYV2|OeLOX1|          ---------KVV----------P--DKAVK-FKVRSVVTVKNKHKEDFK-----------    96
|Q6X5R5|NaLOX3|          ---------KVV----------P--EKAVK-FKVRAVVTVRNKNKEDLK-----------    95
|Q96574|SlLOXd|          ---------KVV----------P--EKAVK-FKVRAVVTVRNKNKEDLK-----------    91
|O24371|StLOX3|          ---------KVV----------P--EKAVR-FKVRAVVTVRNKNKEDLK-----------    97
|A0A1Q4S425|CyLOX|       ------------------------------------------------------------    0
|O16025|PhAOSLOX|        NIANQPASLGLL------EAKSPEDYNSIGELRVAVYTWVQHLRKLKIGSLVPAGQNAIY    376
|P18054|hLOX12|          --------------------------------------------------------MGRY    4
|P16050|hLOX15|          --------------------------------------------------------MGLY    4
|P09917|hLOX5|           --------------------------------------------------------MPSY    4
|O15296|hLOX15B|         --------------------------------------------------------MAEF    4
```

```
|A0A4D6T4S4|AaeLOX|     -----------------------------------------------------------   0
|A0A3G6V563|LiLOX|      -------LRPEIHDLGF---------VEVLLASEDKEAVSE----RMTLKGFSRKPWTNL  131
|P93184|HvLOX2.1|       -------KAAGVRDFLYDLILKTWLHVDLVSSELDPQTGQEREPI----SGAVKHSGR--  144
|Q8GSM3|HvLOX2.2|       SFWWSDEKKERARDWAYDLILGSWLTLELVSSELDPKTGQEHDVI----SGKLKHSRE--  142
|P38419|OsLOX7|         -----NRS-----ID-IRDLIGRSLSLELVSSELDAKTGKEKATV----RSYAHNVDD--  143
|P38418|LOX2|           GITWSRGL-----DD-IADIRGRSLLVELISAKTDQRITVED---------YAQRV-W--  129
|Q8GV01|BnLOX2|         SV-----------G-VTDLLGVSLLVELISAETDPRTLMEKDPV----KDNARRVLL--  126
|Q8GSM2|HvLOX2.3|       SVYVARGL-----DD-IQDLFGKTLLLELVSSELDPKTGRERERV----KGFAHMT-L--  129
|A1XCI5|ZmLOX10|        QMYFQRAV-----DD-IGDLLGKTLLLELVSSELDAKSGVEKTRV----TAYAHKT-L--  138
|A1XCI6|ZmLOX11|        QMYFQRAL-----DD-AGDFFGKTLLMELVSSEVDAKTGLEKPRV----TAFAHKT-L--  142
|E3NYV3|OeLOX2|         HLGLSRGL-----DD-IGDVLGRTLLVELVAAELDPHTGSEKPKI----KAYAHKK-D--  134
|Q6X5R6|NaLOX2|         NLAWTRGL-----DD-IGDLLGKTLLLWIVAAEFDPKTGIKKPSI----KTFAHRG-R--  132
|Q96573|SlLOXc|         NLNLLRGL-----DG-IGDLLGKSLILWIVAAELDHKTGLEKPSI----RSYAHRG-L--  126
|O24370|StLOX2|         NLNLSRGL-----DD-IGDLLGKSLLLWIVAAELDHKTGIEKPGI----RAYAHRG-R--  129
|P08170|SLOX|           --------PDGSAVDNLNAFLGRSVSLQLISATKADAHGKGKVGKDTFLEGINTSLPT--  73
|P14856|PsLOX2|         GVIDSGINIIGSTLDGLTAFLGRSVSLQLISATKSDANGKGKVGKDTFLEGVLASLPT--  99
|Q9CAG3|LOX6|           -------ERFEHQLELFMKAIGQGMLIQLVSEEIDPETGKGRKSLESPVMGLPK--AV--  146
|A0A176VER0|MpLOX|      -------EQMVDRMDVIGDLLGQNISLQLVSMDVDPATKVGKRSRISGIKDWAE-KAA--  168
|A1XCI4|ZmLOX9|         -------EALAGHLDALWDMVGRSVALELISTKIHPRTKKPLHSGQASIKDWCQ-KRG--  153
|Q9LNR3|LOX3|           -------ETLVKHLDAFADKIGRNIVLELISTQLDPKTKLPKKSNAAVLKDWSK-KSK--  151
|Q9FNX8|LOX4|           -------ETLVKHLDAFTDKIGRNVVLELMSTQVDPKTNEPKKSKAAVLKDWSK-KSN--  157
|A0A078ILA7|BnLOX|      -------ETLVKHLDAFTDKIGRNVVLELISIQVDPKTNEPKKSKPAVLKDWSK-KSN--  157
|E3NYV2|OeLOX1|         -------ETIAKRWDAFTDKIGRNVVLELISADIDPKTKGPKKSNQAVLKDWSK-KSN--  146
|Q6X5R5|NaLOX3|         -------ETIVKHLDAFTDKFGRNVSLELISTDIDPNTKGPKKSNQAVLKDWSK-KSN--  145
|Q96574|SlLOXd|         -------ETIVKHLDAFTDKIGRNVALELISTDIDPDTKGPKKSNQAVLKDWSK-KSN--  141
|O24371|StLOX3|         -------ETIVKHLDAFTDKIGRNVTLELISTDMDPNTKGPKKSNQAVLKDWSK-KSN--  147
|A0A1Q4S425|CyLOX|      ------------------------------------MPTSP---------------HFNL  9
|O16025|PhAOSLOX|       NVEVETGDREHAGT-------DATITIRITGAKGRTDYLKL---------------DKWF  414
|P18054|hLOX12|         RIRVATGAWLFSGS-------YNRVQLWLVGTRGEAELELQ---------------L---  39
|P16050|hLOX15|         RIRVSTGASLYAGS-------NNQVQLWLVGQHGEAALGKR---------------L---  39
|P09917|hLOX5|          TVTVATGSQWFAGT-------DDYIYLSLVGSAGCSEKHLL---------------DKPF  42
|O15296|hLOX15B|        RVRVSTGEAFGAGT-------WDKVSVSIVGTRGESPPLPL---------------D-NL  41

|A0A4D6T4S4|AaeLOX|     ---------MVMPFGLPRPLDAIGTVAAIGTGIV----STTVHDAVDAVETVAAIG----  43
|A0A3G6V563|LiLOX|      DGVPKWGAIFTGTLMLPAAMAKPAVMFININHPP-------EGVQYFFANKIALD--G--  180
|P93184|HvLOX2.1|       --VDDEWDMYEATFKVPASFGPIGAVQVTNYHH-----------SEMLLGDIEVFPTGQE  191
|Q8GSM3|HvLOX2.2|       --TEKDYDLYEAIFTCRHRLAPSGAVRLVNYHH-----------TEMLLGEVKIFPAGED  189
|P38419|OsLOX7|         --DDHSVVTYEADFDVPSGFGPIGAIIVTNELR-----------QEMFLEDINLTASDGA  190
|P38418|LOX2|           --AEAPDEKYECEFEMPEDFGPVGAIKIQNQYH-----------RQLFLKGVEL---K--  171
|Q8GV01|BnLOX2|         --DAHGEDQYECVFDMPEDFGPVGAIRVLNQDL-----------KEIFLKEMKL---E--  168
|Q8GSM2|HvLOX2.3|       --K---EGTYEAKMSVPASFGPVGAVLVENEHH-----------REMFIKDIKLITGG--  171
|A1XCI5|ZmLOX10|        --R---EGHYEAEFKVPASFGPVGAVLVENEHH-----------KEVFIKEIKLVTGG--  180
|A1XCI6|ZmLOX11|        --R---EGHYEAEFKVPASFGPVGAVLLENEHH-----------KEIFIREIKLVTGS--  184
|E3NYV3|OeLOX2|         --KDGDETHYESNFNVPEDFGEVGAITIENEHH-----------KEMFVESVVI--DG--  177
|Q6X5R6|NaLOX2|         --DVDDDTHYEADFVIPEDFGEVGAVLVENEHH-----------KEMYVKNIVI--DG--  175
|Q96573|SlLOXc|         --DVDGDTYYEADFEIPEDFGEVGAILVENEHH-----------KEMYVKNIVI--DG--  169
|O24370|StLOX2|         --DVDGDTHYEADFVIPQDFGEVGAILIENEHH-----------KEMYVKNIVI--DG--  172
|P08170|SLOX|           --LGAGESAFNIHFEWDGSMGIPGAFYIKNYMQ-----------VEFFLKSLTLE-AI--  117
|P14856|PsLOX2|         --LGAGESAFNIHFEWDHEMGIPGAFYIKNYMQ-----------VEFFLKSLTLE-DV--  143
|Q9CAG3|LOX6|           --KDPRYLVFTADFTVPINFGKPGAILVTNLLS-----------TEICLSEIIIE-D---  189
|A0A176VER0|MpLOX|      --VVADKVQYTADFVVDKTFGVPGAVVIINRHQ-----------NEFFLETICLQ--G--  211
|A1XCI4|ZmLOX9|         --VKGEHVVYTAEFMVDSDFGEPGAITVANRHH-----------REFFLESIVVE-GG--  197
|Q9LNR3|LOX3|           --TKAERVHYTAEFTVDAAFGSPGAITVMNKHQ-----------KEFFLESITIE--G--  194
|Q9FNX8|LOX4|           --SKAERVHYTAEFTVDSAFGSPGAITVTNKHQ-----------KEFFLESITIE--G--  200
|A0A078ILA7|BnLOX|      --SKAEKVHYAAEFTVDSAFGLPGAITVTNKHQ-----------KEFFLENITIE--G--  200
|E3NYV2|OeLOX1|         --LKTERVNYIAEFLVDSNFGIPGAITVINKHQ-----------QEFFLESITIE--G--  189
|Q6X5R5|NaLOX3|         --LKTERVNYTAEFVVDSNFGTPGAITVTNKHQ-----------QEFFLESITIE--G--  188
|Q96574|SlLOXd|         --LKTERVNYTAEFIVDSNFGNPGAITVTNKHQ-----------QEFFLESITIE--G--  184
|O24371|StLOX3|         --LKTERVNYTAEFIVDSNFGNPGAITVTNKHQ-----------QEFFLESITIE--G--  190
|A0A1Q4S425|CyLOX|      HNPPATNNVDYTKHEYQYNYTRIPPL---------AMVDTLPA-----------------  43
|O16025|PhAOSLOX|       HNDFEAGSKEQYTVQ-GFDVGDIQLIELHSDGG-----GYWSGDPDWFVNRVIIS----  464
|P18054|hLOX12|         --RPARGEEEEFDHDVAEDLGLLQFVRLRKHHW--------LVDDAWFCDRITVQG----  85
|P16050|hLOX15|         --WPARGKETELKVEVPEYLGPLLFVKLRKRHL--------LKDDAWFCNWISVQG----  85
|P09917|hLOX5|          YNDFERGAVDSYDVTVDEELGEIQLVRIEKRKY--------WLNDDWYLKYITLKT----  90
|O15296|hLOX15B|        GKEFTAGAEEDFQVTLPEDVGRVLLLRVHKAPPVLPLLGP-LAPDAWFCRWFQLTP----  96

|A0A4D6T4S4|AaeLOX|     ----TGIVATT----VHDAVD-----LGARLAT------------LPYMGRRNLPTTYHD  78
|A0A3G6V563|LiLOX|      PPGKTAHVDFVINSHVDSGPD------APRPFF---------TA-QAYLPHAPMPDYLAE  224
|P93184|HvLOX2.1|       ES----AVTFHCKSWIDPSHC----TPDKRVFF---------PA-HSYLPSQ-TPKGVEG  232
|Q8GSM3|HvLOX2.2|       PTKSSAVTLFHCQSWIDPSHC----SPDKRTFF---------PVEKSYIPSQ-TPKGVEK  235
|P38419|OsLOX7|         --GNSTVLPIRCNSWVQPKSVGDEGTPSKRIFF---------AN-KTYLPGQ-TPAGLRS  237
|P38418|LOX2|           --LPGGSITFTCESWVAPKSV----DPTKRIFF---------SD-KSYLPSQ-TPEPLKK  214
|Q8GV01|BnLOX2|         --LPDGSVTFTFNSWVAPKSE----DPTKRTFF---------ST-KSYLPLK-TPEPLKQ  211
|Q8GSM2|HvLOX2.3|       --DESTAITFDVASWVHSKFD----DPEPRAFF---------TV-KSYLPSQ-TPPGIEA  214
|A1XCI5|ZmLOX10|        --DSSTAVTFDCNSWVHSKFD----NPEKRIFF---------TL-KSYLPSD-TPKGLED  223
|A1XCI6|ZmLOX11|        --DSSTAVTFGCNSWVHSKFD----NPEKRIFF---------TL-KSYLPSD-TPKGLED  227
|E3NYV3|OeLOX2|         --LYGGPINVTCNSWIHSKFD----NKEPRVFF---------VD-KSYLPSN-TPSGLKI  220
|Q6X5R6|NaLOX2|         --FPHGKVHITCNSWVHSKFD----NPEKRIFF---------TN-KSYLPSQ-TPSAIKR  218
|Q96573|SlLOXc|         --FVHAKVEITCNSWVHSKFA----NPDKRIFF---------TN-KSYLPSQ-TPSGVIR  212
|O24370|StLOX2|         --FVHGKVEITCNSWVHSKFD----NPDKRIFF---------TN-KSYLPSQ-TPSGVSR  215
|P08170|SLOX|           --SNQGTIRFVCNSWVYNTKL----YKSVRIFF---------AN-HTYVPSE-TPAPLVS  160
|P14856|PsLOX2|         --PNHGTIRFVCNSWVYNSKL----YKSPRIFF---------AN-KSYLPSE-TPSPLVK  186
|Q9CAG3|LOX6|           ---STDTILFPANTWIHSKND----NPQARIIF---------RS-QPCLPSE-TPDGIKE  231
|A0A176VER0|MpLOX|      --HESGPIHFPCNSWMHSFKD----NPAQRVFF---------SN-KTYLPST-TPAGLKD  254
|A1XCI4|ZmLOX9|         --LPCGPVHFACNSWVQSTRE----LPGKRVFF---------SN-KPYLPSE-TPPGLRE  240
|Q9LNR3|LOX3|           --FALGPVHFPCNSWVQSQKD----HPDKRIFF---------TN-QPYLPNE-TPSGLRV  237
|Q9FNX8|LOX4|           --FACGPVHFPCNSWVQSQKD----HPSKRILF---------TN-QPYLPSE-TPSGLRT  243
|A0A078ILA7|BnLOX|      --FACGPVHFPCNSWVQSQND----HPSKRIFF---------TN-QPYLPSE-TPSGLRT  243
|E3NYV2|OeLOX1|         --FACGPVHFSCNSWVQSRKD----HPGKRIFF---------SN-QPYLPNE-TPAGLKA  232
|Q6X5R5|NaLOX3|         --FACGPVHFPCNSWVQSKKD----HPGKRIFF---------SN-QPYLPNE-TPAGLKS  231
|Q96574|SlLOXd|         --FACGPVHFPCNSWVQPKKD----HPGKRIFF---------SN-QPYLPDE-TPAGLKS  227
|O24371|StLOX3|         --FACGPVHFPCNSWVQPKKD----HPGKRIFF---------SN-QPYLPDE-TPAGLKS  233
|A0A1Q4S425|CyLOX|      ----KEDFSIAWYRLLVRQLK--------IIFVNTLITNRGNRG-SKSLRDDIRNFIFES  90
|O16025|PhAOSLOX|       -STQDRVYSFPCFRWVIKDMV----------LF---------PG-EATLPFNEVPAIVSE  503
|P18054|hLOX12|         -PGACAEVAFPCYRWVQGEDI--------LSLP---------EG-TARLPGDNALDMFQK  126
|P16050|hLOX15|         -PGAGDEVRFPCYRWVEGNGV--------LSLP---------EG-TGRTVGEDPQGLFQK  126
```

```
|P09917|hLOX5|          -PH-GDYIEFPCYRWITGDVE--------VVLR---------DG-RAKLARDDQIHILKQ    130
|O15296|hLOX15B|        -PR-GGHLLFPCYQWLEGAGT--------LVLQ---------EG-TAKVSWADHHPVLQQ    136
                                           :


|A0A4D6T4S4|AaeLOX|     ALEYSVEG-----------YKQMRYLLDWFFDIAEFQPSSGPVNAVGDLVTTAVNEA-NL    126
|A0A3G6V563|LiLOX|      LREHELAILRGTAAAAKHERKGSERIYDY----DVYNDLGTPTRS-----RPALGG-DTL    274
|P93184|HvLOX2.1|       LRKRELEILRGTG---CGERKEHDRIYDY----DVYNDLGNPDDDNNPTTRPVLGG-KEH    284
|Q8GSM3|HvLOX2.2|       LRKSELEALRGNG---CGERKKHDRIYDY----DVYNDLGKPES-----KRPVLGG-KEH    282
|P38419|OsLOX7|         YRKNDLQQKRGDG---TGEREADDRVYDY----DVYNDLGNPDSN-GDLARPVLGGNKQF    289
|P38418|LOX2|           YRKEELETLQGKNREEVGEFTKFERIYDY----DVYNDVGDPDND-PELARPVIGG-LTH    268
|Q8GV01|BnLOX2|         LRKQELETLQGKNRERAGEFEKFERVYDY----DVYNDLGSPDKD-PELARPILGG-LSH    265
|Q8GSM2|HvLOX2.3|       LRKKELETLRGDG---HSERKFHERVYDY----DTYNDLGDPDKN-IDHKRPVLGT-KEH    265
|A1XCI5|ZmLOX10|        LRKKDLQALRGDG---HGERKVFERVYDY----DVYNDLGDPDKN-PAHQRPVLGGNKQY    275
|A1XCI6|ZmLOX11|        LRKKDLQALRGDG---HGERKASERVYDY----DVYNDLGDPDKN-PAHQRPVLGGSKRY    279
|E3NYV3|OeLOX2|         YREKELQILRGDG---TGERKTFERIYDY----DVYNDLGDPDSS-EDLARPVLGG-QEH    271
|Q6X5R6|NaLOX2|         LRERELVIMRGDG---YGERKQFERIYDY----DVYNDIGDPDA-NDDAKRPVLGG-QEF    269
|Q96573|SlLOXc|         LREGRTRTLRGDG---VGERKVFERIYDY----DVYNDLGEVVSNNDDAKRPILGG-KKL    264
|O24370|StLOX2|         LREEELVTLRGDG---IGERKVFERIYDY----DVYNDLGEADSNNDDAKRPVLGG-KEL    267
|P08170|SLOX|           YREEELKSLRGNG---TGERKEYDRIYDY----DVYNDLGNPDKS-EKLARPVLGGSSTF    212
|P14856|PsLOX2|         YREEELQTLRGDG---TGERKLHERIYDY----DVYNDLGNPDHG-EHLARPILGGSSTH    238
|Q9CAG3|LOX6|           LREKDLVSVRGDG---KGERKPHERIYDY----DVYNDLGDPRKT--ERVRPVLGVP-ET    281
|A0A176VER0|MpLOX|      LREADLKSLRSDG---KGRRKSWENVYDY----AVYNDLGDGDED-AKLIRPVLGGSKAH    306
|A1XCI4|ZmLOX9|         LRDKELKDLRGDG---TGVRKLSDRIYDY----ATYNDLGNPDRG-KEFIRPILGGD-NI    291
|Q9LNR3|LOX3|           LREKELKNLRGDG---SGVRKLSDRIYDF----DVYNDLGNPDKS-SELSRPKLGGK-EV    288
|Q9FNX8|LOX4|           LREKELENLRGNG---KGERKLSDRIYDY----DVYNDIGNPDIS-RELARPTLGGR-EF    294
|A0A078ILA7|BnLOX|      LREQELENLRGNG---KGERKLSDRIYDF----DVYNDIGNPDIS-TELARPVFGGR-EF    294
|E3NYV2|OeLOX1|         LRERELRDLRGDG---QGERKLSDRIYDF----DIYNDLGNPDKG-IDFVRPTLGGE-NI    283
|Q6X5R5|NaLOX3|         LRERELRDLRGDG---TGVRKLSDRVYDY----DIYNDLGNPDKG-IDFARPKLGGSNNV    283
|Q96574|SlLOXd|         LRERELRELRGDG---KGVRKLSDRIYDY----DIYNDLGNPDRG-IDFARPKLGGEGNV    279
|O24371|StLOX3|         LRERELRDLRGDG---KGVRKLSDRIYDY----DIYNDLGNPDKG-IDFARPKLGGDDNV    285
|A0A1Q4S425|CyLOX|      VREEALPTQL--------------NILGRV---LQIAP-QL---L---------------    114
|O16025|PhAOSLOX|       QRQKELEQRK--------------LTYQW----DYVSD-DMPGNI---------------    529
|P18054|hLOX12|         HREKELKDRQ--------------QIYCW----ATWKE-GLPLTI---------------    152
|P16050|hLOX15|         HREEELEERR--------------KLYRW----GNWKD-GLILNM---------------    152
|P09917|hLOX5|          HRRKELETRQ--------------KQYRW----MEWNP-GFPLSI---------------    156
|O15296|hLOX15B|        QRQEELQARQ--------------EMYQW----KAYNP-GWPHCL---------------    162


|A0A4D6T4S4|AaeLOX|     -AIKRVRYNWAH---------NGDFPPHLLIVPVGQFAHDSIHLFKFTRLFDTIKALFPF    176
|A0A3G6V563|LiLOX|      KYPRRLRTGRRVVNGTEV-----AAKGKDWLPPDERFDDRKQENFDSRTLLATLPALSAN    329
|P93184|HvLOX2.1|       PYPRRCRTGRPRSKKDPFSEERSHK-EHIYVPRDEAFTERKMGAFDTKKFMSQLHALTTG    343
|Q8GSM3|HvLOX2.2|       PYPRRCRTGRPRSKTDPSSEEESHKKGEMYVPRDETFTERKEQAFLTKQLLSQLHGLCTG    342
|P38419|OsLOX7|         PYPRRCRTGRPPSKKDPKSETRK--G-NVYVPRDEEFSPEKEDYFLRKTVGSVLQAAVPA    346
|P38418|LOX2|           PYPRRCKTGRKPCETDPSSEQRY--GGEFYVPRDEEFSTAKGTSFTGKAVLAALPSIFPQ    326
|Q8GV01|BnLOX2|         PYPRRCKTGRKPCDKDPSAETRK--ALEFYVPRDEEFTTVKGAQFTGTAVLAALPAVFPQ    323
|Q8GSM2|HvLOX2.3|       PYPRRCRTGRPKTLYDPETETRS--S-PVYVPRDEQFSDVKGRTFSATTLRSGLHAILPA    322
|A1XCI5|ZmLOX10|        PYPRRCRTGRPRTKKDPETEMRE--G-HNYVPRDEQFSEVKQLTFGATTLRSGLHALLPA    332
|A1XCI6|ZmLOX11|        PYPRRCRTGRPRTRKDPEAEVRH--G-HNYVPRDEQFSEVKQLTFGATTLRSGLHALLPA    336
|E3NYV3|OeLOX2|         PYPRRCRTGRARTKTDPLSESRN--G-NVYVPRDEAFSEVKQMQFSAKTIYSVLHSLVPS    328
|Q6X5R6|NaLOX2|         PYPRRCRTGRPRSKTDPLSESRS--N-FVYVPRDEAFSEVKSLTFSGNTLYSVLHAVVPA    326
|Q96573|SlLOXc|         PYPRRCRTGRQRSKKDPLYETRS--T-FVYVPRDEAFSAVKSLTFSGNTVYSALHAVVPA    321
|O24370|StLOX2|         PYPRRCKTGRPRSKKDPLSETRS--T-FVYVPRDEAFSEVKSVAFSGNTVYSVLHAVVPA    324
|P08170|SLOX|           PYPRRGRTGRGPTVTDPNTEKQG---EVFYVPRDENLGHLKSKDALEIGTKSLSQIVQPA    269
|P14856|PsLOX2|         PYPRRGRTGRYPTRKDPNSEKPA---TETYVPRDENFGHLKSSDFLAYGIKSVSQCVVPA    295
|Q9CAG3|LOX6|           PYPRRCRTGRPLVSKDPPCESRGKEKEEFYVPRDEVFEEIKRDTFRAGRFKALFHNLVPS    341
|A0A176VER0|MpLOX|      PYPRRCRTGRPPARHDRSKESRLEGKVVPYVPRDEAFEAVKQESYFSTAIRGLVHQSMPK    366
|A1XCI4|ZmLOX9|         PYPRRCRTGRPPTDTNMLAESRVEKPHRIYVPRDEAFEELKQGAFSSGRLRAVLHTLIPS    351
|Q9LNR3|LOX3|           PYPRRCRTGRQSTVSDKDAESRVEKPLPMYVPRDEQFEESKQDTFAAGRLKAVLHHLIPS    348
|Q9FNX8|LOX4|           PYPRRCRTGRSSTDTDMMSERRVEKPLPMYVPRDEQFEESKQNTFAACRLKAVLHNLIPS    354
|A0A078ILA7|BnLOX|      PYPRRCRTGRRSTDTDLMSERRVEKPLPMYVPRDEQFEESKKNTFAACRLKAVLHNLVPS    354
|E3NYV2|OeLOX1|         PYPRRCRTGRPPTDTDFNAESRVEKPLPMYVPRDEQFEESKMNAFSTGRLKAVLHNLIPS    343
|Q6X5R5|NaLOX3|         PYPRRCRTGRAPTDTDMSAESRVEKPKPLYVPRDEQFEESKMNAFRTGRLKAVLHNLIPS    343
|Q96574|SlLOXd|         AYPRRCRSGRVPTDTDISAESRVEKPNPTYVPRDEQFEESKMNTFSTSRLKATLHNLIPS    339
|O24371|StLOX3|         PYPRRCRSGRVPTDTDISAESRVEKPNPTYVPRDEQFEESKMNTFSTSRLKAVLHNLIPS    345
|A0A1Q4S425|CyLOX|      -----------------------LARMSK----DYREVDELLLSVIKDCGLSIFRDLLGR    147
|O16025|PhAOSLOX|       -----------------------KAKTHDDLPRDVQFTDEKSRSYQESRKAALVNLGIGS    566
|P18054|hLOX12|         -----------------------AADRKDDLPPNMRFHEEKRLDFEWTLKAGALEMALKR    189
|P16050|hLOX15|         -----------------------AGAKLYDLPVDERFLEDKRVDFEVSLAKGLADLAIKD    189
|P09917|hLOX5|          -----------------------DAKCHKDLPRDIQFDSEKGVDFVLNYSKAMENLFINR    193
|O15296|hLOX15B|        -----------------------DEKTVEDLELNIKYSTAKNANFYLQAGSAFAEMKIKG    199


|A0A4D6T4S4|AaeLOX|     IPDFDF---------FKSMAPPSFASVQYLFT----------------------------    199
|A0A3G6V563|LiLOX|      LLAALVPAGLQKLLRTPGSEFMSISDIEQLYSGNAAEDNEDNVLTNLVAPAAAPLAAILG    389
|P93184|HvLOX2.1|       LKTAKH----------KSQSFPSLSAIDQLYDDNFRNQPVQ--------PEG--------    377
|Q8GSM3|HvLOX2.2|       LKVNKD----------ILPSFPTLASIDALYDDDFRNQPVQ--------PEG--------    376
|P38419|OsLOX7|         AQSLLLDK------LKWNLPFPSFFVIDKLFEDGVELPGVD-------------------    381
|P38418|LOX2|           IESVLL-S--------PQEPFPHFKAIQNLFEEGIQLPKDA-------------------    358
|Q8GV01|BnLOX2|         IEAALV-D--------PNMPFPHFKSIEDLFEEGIELPKDA-------------------    355
|Q8GSM2|HvLOX2.3|       VAPLLNNS----------HGFSHFPAIDALYSDGIPLPVDG--------HGG--------    356
|A1XCI5|ZmLOX10|        LRPLLINK--------KDLRFPHFPAIDDLFSDGIPLPAQT-------------------    365
|A1XCI6|ZmLOX11|        IRPMLIK---------RELRFPHFPAIDDLYSDGIPLPAQT-------------------    368
|E3NYV3|OeLOX2|         IETS-IID--------SDLGFPHFTAIETLFNEGVELPKQK--------S-T--------    362
|Q6X5R6|NaLOX2|         LESVAVDD--------PNAGFPHFPAIDSLFNVGVRLPPLN--------DK---------    361
|Q96573|SlLOXc|         LESV-VSD--------PDLGFPHFPAIDSLFNVGVDLSGLS--------DKK--------    356
|O24370|StLOX2|         LESV-VTD--------PNLGFPHFPAIDSLFNVGVDLPGLG--------DKK--------    359
|P08170|SLOX|           FESAFDLK-------STPIEFHSFQDVHDLYEGGIKLPRDV--------IST--------    306
|P14856|PsLOX2|         FESAFDLN-------FTPNEFDSFQDVRNLFEGGIKLPLDV--------IST--------    332
|Q9CAG3|LOX6|           IAAALS---------NLDIPFTCFSDIDNLYKSNIVLGHTE--------PKD--------    376
|A0A176VER0|MpLOX|      LRDHFF---------GSSDEFESFEEIDRLYCEGVELKKVD--------ANE--------    401
|A1XCI4|ZmLOX9|         MIATIS---------AETHSFQGFHHVDNLYKEGLRLKLGL--------QEH--------    386
|Q9LNR3|LOX3|           LKASIV---------A--EDFADFGEIDRLYKEGLLLKLGF--------QDD--------    381
|Q9FNX8|LOX4|           LKASIL---------A--EDFANFGEIDSLYKEGLLLKLGF--------QDD--------    387
|A0A078ILA7|BnLOX|      LKASIL---------A--EDFANFGEIDSLYKEGLLLKLGI--------QDD--------    387
|E3NYV2|OeLOX1|         LMASIS---------ASNHDFKGFSDIDSLYSEGLLLKLGL--------QDE--------    378
|Q6X5R5|NaLOX3|         LMASIS---------TNNHDFKGFSDIDSLYSKGLLLKLGL--------QDE--------    378
|Q96574|SlLOXd|         LMASIS---------SNNHDFKGFSDIDSLYSKGLLVKLGL--------QDE--------    374
|O24371|StLOX3|         LMASIS---------SNNHDFKGFSDIDNLYSKGLLLKLGL--------QDE--------    380
|A0A1Q4S425|CyLOX|      INTRLERG-------HPTGHVSSLNDYS--------------------------------    168
```

```
|O16025|PhAOSLOX|        LFT-------------MFENWDSYDDYH--------------------------------    581
|P18054|hLOX12|          VYT-------------LLSSWNCLEDFD--------------------------------    204
|P16050|hLOX15|          SLN-------------VLTCWKDLDDFN--------------------------------    204
|P09917|hLOX5|           FMH------------MFQSSWNDFADFE--------------------------------    209
|O15296|hLOX15B|         LLD-------------RKGLWRSLNEMK--------------------------------    214


|A0A4D6T4S4|AaeLOX|      ------------------------------------------------------------    199
|A0A3G6V563|LiLOX|       LLTQNSAQGNTPQKVFEQLSDFNKEVVNGALDALVVSLDDLGQSQRAAILHALSLTEKDF    449
|P93184|HvLOX2.1|        ---------------------GKLRFVIDLLETELL------------------------    392
|Q8GSM3|HvLOX2.2|        ---------------------GKVRLILDLLAKELV------------------------    391
|P38419|OsLOX7|          ----------------------KLNFLESVVPRLLE------------------------    395
|P38418|LOX2|            -------------------------GLLPLLPRIIK------------------------    369
|Q8GV01|BnLOX2|          -------------------------GLFPVIPRLAK------------------------    366
|Q8GSM2|HvLOX2.3|        ---------------------NSFNVINDVIPRVVQ------------------------    371
|A1XCI5|ZmLOX10|         ----------------------GFDAFRTVVPRMVK------------------------    379
|A1XCI6|ZmLOX11|         ----------------------GLGAIRSIVPRVVK------------------------    382
|E3NYV3|OeLOX2|          ------------------------GFLANIIPRLVK------------------------    374
|Q6X5R6|NaLOX2|          ------------------------SSLLNIIPRLIK------------------------    373
|Q96573|SlLOXc|          ------------------------SSLFNIVPRLIK------------------------    368
|O24370|StLOX2|          ------------------------SGLFNVVPRLIK------------------------    371
|P08170|SLOX|            ---------------------I------IPLP----------------------------    311
|P14856|PsLOX2|          ---------------------L------SPLP----------------------------    337
|Q9CAG3|LOX6|            ----------------------------TGLGGFIG------------------------    384
|A0A176VER0|MpLOX|       ---------------------FLRMLEDTPLNGMLP------------------------    416
|A1XCI4|ZmLOX9|          ---------------------LFQK---IPLVQ---------------------------    395
|Q9LNR3|LOX3|            ---------------------IFKK---FPLPK---------------------------    390
|Q9FNX8|LOX4|            ---------------------MFKK---FPLPK---------------------------    396
|A0A078ILA7|BnLOX|       ---------------------MFKK---FPLPK---------------------------    396
|E3NYV2|OeLOX1|          ---------------------LSKK---IQLPK---------------------------    387
|Q6X5R5|NaLOX3|          ---------------------MLKK---LPLPK---------------------------    387
|Q96574|SlLOXd|          ---------------------VLKK---LPLPK---------------------------    383
|O24371|StLOX3|          ---------------------VLKK---LPLPK---------------------------    389
|A0A1Q4S425|CyLOX|       ------------------------------------------------------------    168
|O16025|PhAOSLOX|        ------------------------------------------------------------    581
|P18054|hLOX12|          ------------------------------------------------------------    204
|P16050|hLOX15|          ------------------------------------------------------------    204
|P09917|hLOX5|           ------------------------------------------------------------    209
|O15296|hLOX15B|         ------------------------------------------------------------    214

|A0A4D6T4S4|AaeLOX|      ---------------------------RNKLLHYSPSKPLGGNMYTAENVGLREDWFTDA    232
|A0A3G6V563|LiLOX|       QEYAQAGTGRHEVHPVHANFVNLLQEVLHRLLHFSTPA--------VIREGREGAWTTDE    501
|P93184|HvLOX2.1|        --------------HLFKLEGAAFLEGIRRVFKFETPE--------IHDRD-KFAWFRDE    429
|Q8GSM3|HvLOX2.2|        --------------HLVKLEGAEFVEGIRRVFKFETPE--------IHDMD-KLAWFRDE    428
|P38419|OsLOX7|          --------------HLRD-------TPAEKILRFETPA--------NIQKD-KFAWLRDE    425
|P38418|LOX2|            --------------ALGE-------A-QDDILQFDAPV--------LINRD-RFSWLRDD    398
|Q8GV01|BnLOX2|          --------------AAAE---------ADDILQFESPS--------LLDKD-RFSWIRDD    394
|Q8GSM2|HvLOX2.3|        --------------MIED-------T-TEHVLRFEVPE--------MLERD-RFSWFRDE    400
|A1XCI5|ZmLOX10|         --------------LVED-------T-TDHVLRFEVPE--------MIERD-RFSWFKDE    408
|A1XCI6|ZmLOX11|         --------------LVED-------T-TEHVLRFELPD--------MIERD-RFSWFKDE    411
|E3NYV3|OeLOX2|          --------------AITD-------V-EKNVLLFETPQ--------LYERD-RFSWFRDE    403
|Q6X5R6|NaLOX2|          --------------AISD-------T-QKDVLLFETPE--------LLQRD-KFSWFKDV    402
|Q96573|SlLOXc|          --------------SISE-------T-GKDVLLFESPQ--------LVQRD-KFSWFRDV    397
|O24370|StLOX2|          --------------AISD-------T-RKDVLLFESPQ--------LVQRD-KFSWFRDV    400
|P08170|SLOX|            ------------------VIKELYRTDGQHILKFPQPH--------VVQVS-QSAWMTDE    344
|P14856|PsLOX2|          ------------------VVKEIFRTDGEQVLKFTPPH--------VIRVS-KSAWMTDE    370
|Q9CAG3|LOX6|            ------------------GFMNGILNVTETLLKYDTPA--------VIKWD-RFAWLRDN    417
|A0A176VER0|MpLOX|       --------------DIVRAVTSAAGAEQPSVLRYPRPQ--------LLSKD-RFAWLRDD    453
|A1XCI4|ZmLOX9|          ----------------------KIQESSEGMLRYDTPR--------ILSKD-KFAWLRDD    424
|Q9LNR3|LOX3|            ------------------VVVDTLQESTKGLLKYDTPK--------ILSKD-KNAWLRDD    423
|Q9FNX8|LOX4|            ------------------IV-TTLQKSSEGLLRYDTPK--------IVSKD-KYAWLRDD    428
|A0A078ILA7|BnLOX|       ------------------IV-TTLQKSSEGLLRYDTPK--------IVSKD-KYAWLRDD    428
|E3NYV2|OeLOX1|          ------------------AVSKI---QEGGLLKYDIPK--------IISKD-KFAWLRDD    417
|Q6X5R5|NaLOX3|          ------------------VVSSI---QEGDLLKYDTPK--------ILSKD-RFAWLRDD    417
|Q96574|SlLOXd|          ------------------VVSTI---KEGDLLKYDTPK--------ILSKD-KFAWLRDD    413
|O24371|StLOX3|          ------------------VVSSI---KEGDLLKYDTPK--------ILSKD-KFAWLRDD    419
|A0A1Q4S425|CyLOX|       -----------------------------KLLPVI-----------DIPQF-ANSFLEDE    187
|O16025|PhAOSLOX|        -----------------------------ILYRNWILG--------GTPNM-ADRWHEDR    603
|P18054|hLOX12|          -----------------------------QIFWGQKSA--------LAEKV-RQCWQDDE    226
|P16050|hLOX15|          -----------------------------RIFWCGQSK--------LAERV-RDSWKEDA    226
|P09917|hLOX5|           -----------------------------KIFVKISNT--------ISERV-MNHWQEDL    231
|O15296|hLOX15B|         -----------------------------RIFNFRRTP--------AAEHA-FEHWQEDA    236
                                                       :                        :  *

|A0A4D6T4S4|AaeLOX|      VFAQQQFTGVNPCTITAASQSWIQAF--------------TQAAR-D--QGNRSAAKLFT    275
|A0A3G6V563|LiLOX|       EWGREQLAGQNACMIHAIWNDAPIKD------LPA-DSAITEAVLQGHLEGH-SISQLLS    553
|P93184|HvLOX2.1|        EFARQTIAGMNPMSIQLVTEFPIKSNLDEATYGPA-DSLITKEVVEEQIRRV--MTADEA    486
|Q8GSM3|HvLOX2.2|        EFARQTLAGMNPLSIQLVTELPIVSKLDELKYGPA-DSLITKELIEKQINRI--MTAEEA    485
|P38419|OsLOX7|          EFARETLAGINPYAIELVREFPLKSKLDPAVYGPA-ESAITADLLEEQMRRV--MTVEEA    482
|P38418|LOX2|            EFARQTLAGLNPYSIQLVEEWPLISKLDPAVYGDP-TSLITWEIVEREVKGN--MTVDEA    455
|Q8GV01|BnLOX2|          EFVRQTLAGLNPYCIQLVQEWPLKSKLDPAVYGDP-NSLITSEIVEREIKGV--MSFDEA    451
|Q8GSM2|HvLOX2.3|        EFARQTLAGLNPICIRRLTEFPIVSKLDPAVYGPA-ESALSKEILEKMMNGR--MTVEEA    457
|A1XCI5|ZmLOX10|         EFARQTIAGLNPLCIQLLTEFPIKSKLDPEVYGPA-ESAITKEILEKQMNGA--LTVEQA    465
|A1XCI6|ZmLOX11|         EFARQTIAGLNPLCIQLLTEFPIRSKLDREVYGPA-ESAITKEILEKHMSGGAAMTVEQA    470
|E3NYV3|OeLOX2|          EFARQTLAGINPCRIELVTEWPLKSKLDPAVYGPA-ESAITTELVEKEIGGF--TTVKKA    460
|Q6X5R6|NaLOX2|          EFARQTLAGLNPYSIRLVTEWPLKSKLDPEIYGPP-ESAITKELIELEIAGF--MTVEEA    459
|Q96573|SlLOXc|          EFARQTLAGLNPYSIRLVTEWPLRSNLDPKVSGPP-ESEITKELIENEIGNN--MTVEQA    454
|O24370|StLOX2|          EFARQTLAGLNPYSIRLVTEWPLRSKLDPKVYGPP-ESEITKELIEKEIGNY--MTVEQA    457
|P08170|SLOX|            EFAREMIAGVNPCVIRGLEEFPPKSNLDPAIYGDQ-SSKIT----ADSLDLD-GYTMDEA    398
|P14856|PsLOX2|          EFAREMLAGVNPCMIRGLQEFPPKSNLDPAEYGDH-TSKIS----VDVLNLD-GCTIDEA    424
|Q9CAG3|LOX6|            EFGRQALAGVNPVNIELLKELPIRSNLDPALYGPQ-ESVLTEEIIAREVEHY-GTTIEKA    475
|A0A176VER0|MpLOX|       EFGRQTLAGLNPCAIQRLKVFPPMSELDPKVYGSP-ESAIKEEHIADRLE---GLSVRQA    509
|A1XCI4|ZmLOX9|          EFARQTVAGINPVSITRLTVFPPVSKMDPAIYGPP-ESSITEAHITGQLN---GLTVQQA    480
|Q9LNR3|LOX3|            EFARQAIAGINPVNIERVKTFPPVSNLDPKIYGPQ-HSALTDDHIIGHLD---GFSVQQA    479
|Q9FNX8|LOX4|            EFARQAIAGINPVNIERVTSYPPVSNLDPEIYGPGLHSALTEDHIIGQLD---GLTVQQA    485
|A0A078ILA7|BnLOX|       EFARQAIAGINPVNIERVTTYPPVSNLDREIYGSHLDSALTEDHIIGHLD---GLSVQQA    485
|E3NYV2|OeLOX1|          EFGRQAIAGVNPVNIERLQSFPPVCKLDPEIYGPQ-ESALKEEHIVGHLN---GMTVQEA    473
|Q6X5R5|NaLOX3|          EFARQAIAGVNPVNIERLQVFPPVSKLDPEIYGTQ-ESALKEEHILGHLN---GMTVQEA    473
```

```
|Q96574|SlLOXd|          EFARQAIAGVNPVSIEKLQVFPPVSKLDPEIYGPQ-ESALKEEHILGHLN---GMTVQEA    469
|O24371|StLOX3|          EFARQAIAGVNPVSIEKLQFFPPVSKLDPEIYGPQ-ESALKEEHILGHLN---GMTVQEA    475
|A0A1Q4S425|CyLOX|       SFAYMQVAGFNPLMIERVSSFGANFP-------------IEDSHYQAVMGSD--DSLELA    232
|O16025|PhAOSLOX|        WFGYQFLNGANPVILTRCDALPSNFP-------------VTNEHVNASLDRG--KNLDEE    648
|P18054|hLOX12|          LFSYQFLNGANPMLLRRSTSLPSRLV-------------LPSGMEE----LQ--AQLEKE    267
|P16050|hLOX15|          LFGYQFLNGANPVVLRRSAHLPARLV-------------FPPGMEE----LQ--AQLEKE    267
|P09917|hLOX5|           MFGYQFLNGCNPVLIRRCTELPEKLP-------------VTTEMVECSLERQ--LSLEQE    276
|O15296|hLOX15B|         FFASQFLNGLNPVLIRRCHYLPKNFP-------------VTDAMVASVLGPG--TSLQAE    281
                          :   . * *  :

|A0A4D6T4S4|AaeLOX|      SSTNKFYVQDCSY-FREAA---------GMT-PTEAIYSNDKSSHRYGCASVSLFHLLP-    323
|A0A3G6V563|LiLOX|       GDKPRLFLIDYMKGFKDYAE----------------KIAAA-HPGNVMYAGRAVLYLRT-    595
|P93184|HvLOX2.1|        VQNKKLFMLDYHDLLLPYVH----------------KVRKL--DGTTLYGSRALFFLTA-    527
|Q8GSM3|HvLOX2.2|        VAQKKLFMLDYHDLLLPYVH----------------RVRKL--DNKTMYGSRTLFFLAD-    526
|P38419|OsLOX7|          ISQKRLFMLDFHDLFLPYVH----------------KIRSL--DHTTMYGSRTVFFLTD-    523
|P38418|LOX2|            LKNKRLFVLDYHDLLLPYVN----------------KVREL--NNTTLYASRTLFFLSD-    496
|Q8GV01|BnLOX2|          LENKRLFMLDYHDLLLPYVN----------------KVREL--DDSTLYASRALFFLSD-    492
|Q8GSM2|HvLOX2.3|        MEKKRLFLLDYHDVFLPYVH----------------RVREL--PDTTLYGSRTVFFLSD-    498
|A1XCI5|ZmLOX10|         LAAKRLFILDYHDVFLPYVH----------------KVREL--QDATLYASRTIFFLTD-    506
|A1XCI6|ZmLOX11|         LAAKRLFILDYHDVFLPYVH----------------RVREL--PDTTLYGSRTVFFLTD-    511
|E3NYV3|OeLOX2|          LEEKKLFVLDYHDLFIPYVK----------------KVREI--KGTTLYGSRTLFFLMP-    501
|Q6X5R6|NaLOX2|          VKQKKLFILDYHDLLLPYVN----------------KVNEL--KGRVLYGSRTLFFLTP-    500
|Q96573|SlLOXc|          VQQKKLFILDYHDLLLPYVN----------------KVNEL--KGSVLYGSRTIFFLTP-    495
|O24370|StLOX2|          VQQKKLFILDYHDLLLPYVN----------------KVNEL--KGSMLYGSRTIFFLTP-    498
|P08170|SLOX|            LGSRRLFMLDYHDIFMPYVR----------------QINQL--NSAKTYATRTILFLRE-    439
|P14856|PsLOX2|          LASGRLFILDYHDTFIPFLR----------------RINET---SAKAYATRTILFLKE-    464
|Q9CAG3|LOX6|            LEEKRLFLVDYHDILLPFVE----------------KINSIKEDPRKTYASRTIFFYSK-    518
|A0A176VER0|MpLOX|       LKEARLFILDYHDVFMPYVN----------------GINSL--EKRAGYASRTIYFLTS-    550
|A1XCI4|ZmLOX9|          VDEAKLFILDYHDVYMPFLD----------------RINAI--EGRKAYATRTILFLTK-    521
|Q9LNR3|LOX3|            LEENRLYMLDYHDIFLPFLD----------------RINAL--DGRKAYATRTIFFLTR-    520
|Q9FNX8|LOX4|            LETNRLFMVDYHDIYLPFLD----------------RINAL--DGRKAYATRTILFLTR-    526
|A0A078ILA7|BnLOX|       LETNRLFMVDYHDTYLPFLD----------------RINAL--DGRKAYATRTILFLTR-    526
|E3NYV2|OeLOX1|          LEANKLFIIDYHDIYLPFLD----------------GINAL--DGRKEYATRTIFFLTD-    514
|Q6X5R5|NaLOX3|          LDANRLYIVDYHDVYLPFLD----------------RINAL--DGRKAYATRTIFFLSD-    514
|Q96574|SlLOXd|          LDANKLFILDHHDVYLPFLD----------------RINAL--DGRKAYATRTIYFLSD-    510
|O24371|StLOX3|          LDANKLFIVDHHDVYLPFLD----------------RINAL--DGRKAYATRTIFFLSD-    516
|A0A1Q4S425|CyLOX|       LKEGRLYLADYKILDNAING----------------T-YSR--YQKYLYAPIALFAVPKA    273
|O16025|PhAOSLOX|        IKDGHIYIVDFKVLVGAKSYGGPVLEDIGYKVPDHLKHDEA--DIRYCAAPLALFYVNK-    705
|P18054|hLOX12|          LQNGSLFEADFILLDGIPAN----------------VIRG---EKQYLAAPLVMLKMEP-    307
|P16050|hLOX15|          LEGGTLFEADFSLLDGIKAN----------------VILC---SQQHLAAPLVMLKLQP-    307
|P09917|hLOX5|           VQQGNIFIVDFELLDGIDAN----------------KTDPC--TLQFLAAPICLLYKNL-    317
|O15296|hLOX15B|         LEKGSLFLVDHGILSGIQTN----------------VING---KPQFSAAPMTLLYQSPG    322
                             ::  *                                .   :

|A0A4D6T4S4|AaeLOX|      T---GKLHPIAIILDWKGSIDPNVSVTIFNRRLSPSLIPAV-SLLEEASDWPWRYAKMCA    379
|A0A3G6V563|LiLOX|       D---GELVPVAIELQAPRRKLE-------------AF-------TSADSPTIWLLAKCIF    632
|P93184|HvLOX2.1|        D---GTLRPIAIELTRPKSKKK--P------QWRQVFTPGC--DGSVTGSWLWQLAKAHI    574
|Q8GSM3|HvLOX2.2|        D---GTLRPIAIELTRPKSPHK--Q------QWRKVFTPGSGYSGSVTGSWEWQLAKIHV    575
|P38419|OsLOX7|          D---GTLQLLAIELTRPASPSQ--P------QWRQVFTP-S---TDATMSWLWRMAKAHV    568
|P38418|LOX2|            D---STLRPVAIELTCPPNINK--P------QWKQVFTP-G---YDATSCWLWNLAKTHA    541
|Q8GV01|BnLOX2|          D---STLRPVAIELTRPQDVNR--P------QWRQVFTP-G---YDATSCWLWILAKTHV    537
|Q8GSM2|HvLOX2.3|        E---GTLMPLAIELTRPQSPTK--P------QWKRAFTH-G---SDATESWLWKLAKAHV    543
|A1XCI5|ZmLOX10|         L---GTLMPLAIELTRPKSPTR--P------QWKRAFTH-G---PDATDAWLWKLAKAHV    551
|A1XCI6|ZmLOX11|         L---GTLMPLAIELVRPKSPTQ--P------QWKRVFTH-G---PDATDAWLWKLAKAHV    556
|E3NYV3|OeLOX2|          S---GTLRPLAIELTRPQIDGK--P------QWKEVFQP-C---WDATGVWLWRLAKAHV    546
|Q6X5R6|NaLOX2|          D---GTLRPLAIELTRPPVHDK--P------QWKEVYCP-T---WHATGSWLWKLAKAHV    545
|Q96573|SlLOXc|          H---GTLKPLAIELTRPPIDDK--P------QWKEVYSPNN---WNATGAWLWKLAKAHV    541
|O24370|StLOX2|          Q---GTLKPLAIELTRPPVDDK--P------QWKEVYSPND---WNATGAWLWKLAKAHV    544
|P08170|SLOX|            D---GTLKPVAIELSLPHSAGDLSA------AVSQVVLP-A---KEGVESTIWLLAKAYV    486
|P14856|PsLOX2|          N---GTLKPVAIELSLPHPDGDKSG------FVSKVILP-A---DEGVESTIWLLAKAYV    511
|Q9CAG3|LOX6|            N---GALRPLAIELSLPPTAES---------ENKFVYTH-G---HDATTHWIWKLAKAHV    562
|A0A176VER0|MpLOX|       E---GTLKPLVIELCLPPP-----H------RSQRVFVP-G---HSATEHWLWQLAKAHV    592
|A1XCI4|ZmLOX9|          A---GTLKPIAIELSLPPSKAGE-P------RPSKVLTP-P---ADATSNWLWMLAKAHV    567
|Q9LNR3|LOX3|            L---GTLKPVAIELSLPPHGPK--H------RSKRVLTP-P---VDATSNWMWQLAKAHV    565
|Q9FNX8|LOX4|            L---GTLKPIAIELSLPSQSSSN-Q------KSKRVVTP-P---VDATSNWMWQLAKAHV    572
|A0A078ILA7|BnLOX|       L---GTLKPVAIELSLP--------------KSKRVVTP-P---IDATSHWTWQLAKAHV    565
|E3NYV2|OeLOX1|          L---GTLKPIAIELSLPPTAPS--S------RSKQVVTP-P---VDATTDWMWKLAKAHV    559
|Q6X5R5|NaLOX3|          L---GTLKPIAIELSLPQTGPS--S------RSKRVVTP-P---VDATGNWMWQLAKAHV    559
|Q96574|SlLOXd|          V---GTLKPIAIELSLPQTGPS--S------RSKRVVTP-P---VCATGNWMWQIAKAHV    555
|O24371|StLOX3|          V---GTLKPIAIELSLPQTGPS--S------RSKRVVTP-P---VCATGNWTWQIAKAHV    561
|A0A1Q4S425|CyLOX|       ANSNRSLQAVGIQC--GQNPGA-HP----------IITP------KS-NRYAWLFARTIV    313
|O16025|PhAOSLOX|        L---GHLMPIAIQI--NQEPGPENP----------IWTP------HEENEHDWMMAKFWL    744
|P18054|hLOX12|          N---GKLQPMVIQIQPPNPSSPTPT----------LFLP------SD-PPLAWLLAKSWV    347
|P16050|hLOX15|          D---GKLLPMVIQLQLPRTGSPPPP----------LFLP------TD-PPMAWLLAKCWV    347
|P09917|hLOX5|           A---NKIVPIAIQL--NQIPGDENP----------IFLP------SD-AKYDWLLAKIWV    355
|O15296|hLOX15B|         C---GPLLPLAIQL--SQTPGPNSP----------IFLP------TD-DKWDWLLAKTWV    360
                              : : *                                  * *:

|A0A4D6T4S4|AaeLOX|      QASDWVRHEVGVHLTLTHLVEEAIIVAAQRSLPDSHIVHTLLSPHWLRT-LSLNSMARMI    438
|A0A3G6V563|LiLOX|       SSIDAGYHQLISHFVRAHACTEPYIIATRRQLSVMHPVFKLLITHCRFT-LNVNSNARQQ    691
|P93184|HvLOX2.1|        LAHDAGVHQLVSHWLRTHACTEPYIIAANRQLSQMHPVYRLLHPHFRFT-MEINAQARAM    633
|Q8GSM3|HvLOX2.2|        LSHDTGYHQLVSHWLRTHCCVEPYVIAANRQLSQMHPIYRLLHPHFRFT-MEINAQARGM    634
|P38419|OsLOX7|          RAHDAGHHELITHWLRTHCAVEPYIIAANRQLSEMHPIYQLLRPHFRYT-MRINARARSA    627
|P38418|LOX2|            ISHDAGYHQLISHWLRTHACTEPYIIAANRQLSAMHPIYRLLHPHFRYT-MEINARARQS    600
|Q8GV01|BnLOX2|          ISHDAGYHQLISHWLRTHCCIEPYIIAANRQLSAMHPIYRLLHPHFRYT-MEINARARQV    596
|Q8GSM2|HvLOX2.3|        LTHDTGYHQLVSHWLRTHACVEPYIIATNRQLSRMHPVYRLLHPHFRYT-MEINALAREA    602
|A1XCI5|ZmLOX10|         LTHDTGYHQLVSHWLRTHCCVEPYIIAANRQLSRLHPVYRLLHPHFRYT-MEINALAREA    610
|A1XCI6|ZmLOX11|         VTHDTGYHQLVSHWLRTHCCVEPYIIAANRQLSRLHPVYRLLHPHFRYT-MEINALAREA    615
|E3NYV3|OeLOX2|          LAHDSGFHQLVSHWLRTHCCTEPYVIATNRQLSAMHPVYKLLHPHLRYT-MEINALAREA    605
|Q6X5R6|NaLOX2|          LAHDSGYHQLVSHWLRTHCATEPYIIATNRQLSAIHPIYRLLHPHFRYT-MEINALAREA    604
|Q96573|SlLOXc|          LSHDSGYHQLVSHWLRTHCCTEPYIIATNRQLSAMHPIYRLLHPHFRYT-MEINALAREA    600
|O24370|StLOX2|          LSHDSGYHQLVSHWLRTHCCTEPYIIASNRQLSAMHPIYRLLHPHFRYT-MEINALAREA    603
|P08170|SLOX|            IVNDSCYHQLMSHWLNTHAAMEPFVIATHRHLSVLHPIYKLLTPHYRNN-MNINALARQS    545
|P14856|PsLOX2|          VVNDSCYHQLMSHWLNTHAVIEPFVIATNRQLSVVHPINKLLAPHYRDTMMNINALARDS    571
|Q9CAG3|LOX6|            CSNDAGVHQLVNHWLRTHASMEPYIIATNRQLSTMHPVYKLLHPHMRYT-LEINARARKS    621
|A0A176VER0|MpLOX|       SCNDAGFHQLVSHWLRTHAATEPYVIATNRQLSIMHPVYRLLHPHFRYT-MEINAAARQS    651
|A1XCI4|ZmLOX9|          SSNDAGVHQLVNHWLRTHAVMEPFILAAHRRMSAMHPVFKLLHPHMRYT-LEINALARQS    626
|Q9LNR3|LOX3|            SSNDAGVHQLVNHWLRTHACLEPFILAAHRQLSAMHPIFKLLDPHMRYT-LEINALARQS    624
|Q9FNX8|LOX4|            GSNDAGVHQLVNHWLRTHACLEPFILAAHRQLSAMHPIFKLLDPHMRYT-LEINAVARQT    631
```

```
|A0A078ILA7|BnLOX|       GSNDAGVHQLVNHWLRTHACLEPFILAAHRQLSALHPIYKLLDPHMRYT-LEINAVARQT    624
|E3NYV2|OeLOX1|          CANDAGVHQLVNHWLRTHATIEPFILAAHRQLSAMHPIFKLLDPHMRYT-LEINALARQS    618
|Q6X5R5|NaLOX3|          CSNDAGVHQLVNHWLRTHACLEPFILAAHRQLSAMHPIYKLLDPHMRYT-LEINALARQS    618
|Q96574|SlLOXd|          CANDAGVHQLVNHWLRTHASLEPFILAAHRQLSAMHPIYKLLDPHMRYT-LEINGLARQS    614
|O24371|StLOX3|          CANDAGVHQLVNHWLRTHASLEPFILAAHRQLSAMHPIYKLLDPHMRYT-LEINGLARQS    620
|A0A1Q4S425|CyLOX|       TIADTNYHEAVSHLGRTHLFVGAFVLATRRQLPDNHPLSILLRPHFEGT-LAINDAAQRL    372
|O16025|PhAOSLOX|        GVAESNFHQLNTHLLRTHLTTESFALSTWRNLASAHPVFKLLQPHIYGV-LAIDTIGRKE    803
|P18054|hLOX12|          RNSDFQLHEIQYHLLNTHLVAEVIAVATMRCLPGLHPIFKFLIPHIRYT-MEINTRARTQ    406
|P16050|hLOX15|          RSSDFQLHELQSHLLRGHLMAEVIVVATMRCLPSIHPIFKLIIPHLRYT-LEINVRARTG    406
|P09917|hLOX5|           RSSDFHVHQTITHLLRTHLVSEVFGIAMYRQLPAVHPIFKLLVAHVRFT-IAINTKAREQ    414
|O15296|hLOX15B|         RNAEFSFHEALTHLLHSHLLPEVFTLATLRQLPHCHPLFKLLIPHTRYT-LHINTLAREL    419
                            :   *:   *    *       ::  * :   * :  ::  *     : ::  .:

|A0A4D6T4S4|AaeLOX|      LVPDMIAALAPFKLT--EIKALCNYTYHSFDFAGHYIPEDLKNRGFDVAHFDNPFG---Q    493
|A0A3G6V563|LiLOX|       LINAGGIIEGNFTPGRYAMELSSVVYGLTWTFDSQALPHDLVNRGVARREKD--GTLKLL    749
|P93184|HvLOX2.1|        LINAGGIIEGSFVPGEYSLELSSVAYDQQWRFDMEALPEDLIRRGMAVRNPN--GELELA    691
|Q8GSM3|HvLOX2.2|        LICADGIIEKTFSPGEFSMEISSAAYDKQWRFDMEALPEDLIRRGMAVRGED--GKLELA    692
|P38419|OsLOX7|          LISAGGIIERSFSPQKYSMELSSVAYDKLWRFDTEALPADLVRRGMAEEDPTAEHGLKLA    687
|P38418|LOX2|            LVNGGGIIETCFWPGKYALELSSAVYGKLWRFDQEGLPADLIKRGLAEEDKTAEHGVRLT    660
|Q8GV01|BnLOX2|          LINEGGIIESRFWPGKYSLELSSDVYDKLWRFDREGLPADLIGRGLAVEDETAEHGVRLT    656
|Q8GSM2|HvLOX2.3|        LINADGIIEEAFLAGKYSIELSSVAYGAAWQFNTEALPEDLINRGLAVRRDD--GELELA    660
|A1XCI5|ZmLOX10|         LINADGIIEESFWPGKYAVELSSVAYGATWQFDTEALPNDLIKRGLAVRGED--GELELT    668
|A1XCI6|ZmLOX11|         LINADGIIEESFWPGRYAVELSSAAYGATWQFDTEALPNDLVKRGLAVRRDD--GELELT    673
|E3NYV3|OeLOX2|          LINANGVIEKGFTPRKYSLEVSSAAYDQLWQFDLQALPADLISRGMAVEDPAAPHGLKLA    665
|Q6X5R6|NaLOX2|          LINANGIIESSFFPGKYAMELSSVAYDLEWRFDREALPEDLISRGMAVKDPNAPYGLKLT    664
|Q96573|SlLOXc|          LINANGIIESSFFPGKYSVELSSIAYGAEWRFDQEALPQNLISRGLAEEDPNEPHGLKLA    660
|O24370|StLOX2|          LINANGVIESSFFPGKYAIELSSIAYGAEWRFDQEALPQNLISRGLAVEDPNEPHGLKLA    663
|P08170|SLOX|            LINANGIIETTFLPSKYSVEMSSAVYKN-WVFTDQALPADLIKRGVAIKDPSTPHGVRLL    604
|P14856|PsLOX2|          LINANGLIERSFLPSKYAVEMSSAVYKY-WVFTDQALPNDLIKRNMAVKDSSSPYGLRLL    630
|Q9CAG3|LOX6|            LINGGGIIESCFTPGKYAMELSSAAYKSMWRFDMEGLPADLVRRGMAEEDSSAECGVRLV    681
|A0A176VER0|MpLOX|       LICADGVIEKSFQPGPAAMEISSAYYAASWRFDRQGLPEDLIDRGMAVEDPSATHGLRLA    711
|A1XCI4|ZmLOX9|          LISADGVIESCFTPGPVSFEISAAYYRDHWRFDLEGLPSDLVRRRVAVEDASQPHGIRLL    686
|Q9LNR3|LOX3|            LISADGVIEGGFTAGAYGMEMSAAAYKSSWRFDMEGLPADLIRRGMAIPDATQPHGLKLL    684
|Q9FNX8|LOX4|            LISADGVIESCFTAGQYGLEISSAAYKNKWRFDMEGLPADLIRRGMAVPDPTQPHGLKLL    691
|A0A078ILA7|BnLOX|       LVSADGVIESCFTAGQYGLEISAAAYKNQWRFDMEGLPADLIRRGMAVPDPTQPHGLKLL    684
|E3NYV2|OeLOX1|          LISADGVIESCFTPGRYCMEISAAAYRNFWRFDLEGLPADLIRRGMAVPDPTQPHGLKLL    678
|Q6X5R5|NaLOX3|          LISADGVIEACFTPGRYCMEMSAAAYKNLWRFDLEGLPADLIRRGMAVPDPTQPHGLKLL    678
|Q96574|SlLOXd|          LINADGVIEACFTPGRYCMEISAAAYKN-WRFDLEGLPADLIRRGMAVPDATQPYGLKLL    673
|O24371|StLOX3|          LINADGVIEACFTPGRYCMEISAAAYKN-WRFDLEGLPADLIRRGMAVPDSTQPHGLKLL    679
|A0A1Q4S425|CyLOX|       LIAPVGAVDQLLGSTIDNSRVLVALGLRSYGFNGAMLPKQLKQRGVDDP---------NL    423
|O16025|PhAOSLOX|        LIGSGGIVDQSLSLGGGGHVTFMEKCFKEVNLQDYHLPNALKKRGVDDP---------SK    854
|P18054|hLOX12|          LISDGGIFDKAVSTGGGGHVQLLRRAAAQLTYCSLCPPDDLADRGL------------LG    454
|P16050|hLOX15|          LVSDMGIFDQIMSTGGGGHVQLLKQAGAFLTYSSFCPPDDLADRGL------------LG    454
|P09917|hLOX5|           LICECGLFDKANATGGGGHVQMVQRAMKDLTYASLCFPEAIKARGMESK---------ED    465
|O15296|hLOX15B|         LIVPGQVVDRSTGIGIEGFSELIQRNMKQLNYSLLCLPEDIRTRGV------------ED    467
                         *:                                   *  :  * .

|A0A4D6T4S4|AaeLOX|      FHNYAYARDMAKMWDVMHKFVGSALSAHYKGS--DAEVRADKYVADFCAEMQSSVGG---    548
|A0A3G6V563|LiLOX|       MADYPYAADGLLVWDAFVEWFDSYLRLYYDDEVDGKRVTDDPEITAWWTEIQEKGHP---    806
|P93184|HvLOX2.1|        IEDYPYANDGLLVWDAIKQWALTYVQHYYPCA---ADIVDDEELQAWWTEVRTKGHA---    745
|Q8GSM3|HvLOX2.2|        IEDYPYANDGLLVWDAIKQWASDYVAHYYPCA---VDIVDDEELQDWWTEVRTKGHP---    746
|P38419|OsLOX7|          IEDYPFANDGLLIWDAIKTWVQAYVARFYPDA---DSVAGDEELQAFWTEVRTKGHG---    741
|P38418|LOX2|            IPDYPFANDGLILWDAIKEWVTDYVKHYYPDE---ELITSDEELQGWWSEVRNIGHG---    714
|Q8GV01|BnLOX2|          IPDFPFANDGLMLWDALKEWVTDYVNHYYPDS---EQVTLDEELQGWWSEVRNIGHG---    710
|Q8GSM2|HvLOX2.3|        IKDYPYADDGLLIWGSIKQWASDYVDFYYKSD---GDVAGDEELRAWWEEVRTKGHA---    714
|A1XCI5|ZmLOX10|         IKDYPYAHDGLLVWDSIRQWASEYVNVYYKSD---EAVAADPELRAFWDEVRNVGHG---    722
|A1XCI6|ZmLOX11|         IKDYPYAHDGLMVWNTIRQWAADYVNVYYKSD---EAVAADPELKAFWDEVRNVGHG---    727
|E3NYV3|OeLOX2|          IEDYPYANDGLLIWDAIKKWVTDYVTYYYPEA---NLVQSDVELQEWWTEIRTKGHA---    719
|Q6X5R6|NaLOX2|          IEDYPFANDGLVLWDILIQWVTDYVNHYYTET---KLIESDTELQAWWTEIKNVGHG---    718
|Q96573|SlLOXc|          IEDYPFANDGLVLWDILKQWVTNYVNHYYPQT---NLIESDKELQAWWSEIKNVGHG---    714
|O24370|StLOX2|          IEDYPFANDGLVLWDILKQWVTNYVNHYYPQT---NLIESDKELQAWWSEIKNVGHG---    717
|P08170|SLOX|            IEDYPYAADGLEIWAAIKTWVQEYVPLYYARD---DDVKNDSELQHWWKEAVEKGHG---    658
|P14856|PsLOX2|          IEDYPYAVDGLEIWTAIKTWVQDYVSLYYATD---NDIKNDSELQHWWKEVVEKGHG---    684
|Q9CAG3|LOX6|            IDDYPYAADGLLIWKAIKDLVESYVKHFYSDS---KSITSDLELQAWWDEIKNKGHY---    735
|A0A176VER0|MpLOX|       IEDYPFAADGLLMWTALETWVREYVAIYYKDP---ESVRSDHELLSWWAEIRYSGHA---    765
|A1XCI4|ZmLOX9|          IEDYPYANDGLLLWSAIRSWVESYVQLYYPDA---GTVQSDDELQGWYHETVHVGHA---    740
|Q9LNR3|LOX3|            IEDYPYANDGLLLWSAIQTWVRTYVERYYPNP---NLIKTDSELQSWYSESINVGHA---    738
|Q9FNX8|LOX4|            VEDYPYANDGLLLWSAIQTWVRTYVERYYANS---NLIQTDTELQAWYSESINVGHA---    745
|A0A078ILA7|BnLOX|       VEDYPYANDGLLLWSAIQTWVRTYVERYYANP---NLVKTDKELQAWYSESINVGHA---    738
|E3NYV2|OeLOX1|          IEDYPYATDGLMIWTAIENWVRSYVNHYYLDS---SLVCNDKELQAWYAESINVGHA---    732
|Q6X5R5|NaLOX3|          IEDYPYAADGLMIWAAIEGWVRSYVNHYYPDS---AQVCNDRELQAWYAESINVGHA---    732
|Q96574|SlLOXd|          IEDYPYAADGLMIWGAIEGWVRDYVDHYYPSS---AQVCSDRELQAWYTETINVGHV---    727
|O24371|StLOX3|          IEDYPYAADGLMIWGAIESWVRDYVNHYYPSS---AQVCSDRELQAWYAETINVGHV---    733
|A0A1Q4S425|CyLOX|       LPVYPYRDDALLIWNAIHQWVSDYLSIYYSTN---QDVQNDTALQAWAAEVQAFDGG---    477
|O16025|PhAOSLOX|        LPGFYYRDDGLALWEAIETFIGEIIAIFYKND---DDVKRDNEIQSWIYDVHKNGWRVNP    911
|P18054|hLOX12|          LPGALYAHDALRLWEIIARYVEGIVHLFYQRD---DIVKGDPELQAWCREITEVGLC---    508
|P16050|hLOX15|          VKSSFYAQDALRLWEIIYRYVEGIVSLHYKTD---VAVKDDPELQTWCREITEIGLQ---    508
|P09917|hLOX5|           IPYYFYRDDGLLVWEAIRTFTAEVVDIYYEGD---QVVEEDPELQDFVNDVYVYGMR---    519
|O15296|hLOX15B|         IPGYYYRDDGMQIWGAVERFVSEIIGIYYPSD---ESVQDDRELQAWVREIFSKGFL---    521
                         .    :  *   :*  .       :  .*        :  *  :  :  :

|A0A4D6T4S4|AaeLOX|      ---QMPQFPTVKTLADLVDVVTMCIHIAAPQHTSINYLQQYYLTYVPSRPSALFAPPPTS    605
|A0A3G6V563|LiLOX|       DIKE--GWPQLQTIADLTQILTTIAWIASAHHAAINYGQYDYSGFMPNRSPMIRKAMPPK    864
|P93184|HvLOX2.1|        DKQDEPWWPELDSHENLAQTLATIMWVTSGHHAAVNFGQYPMAGYIPNRPTMARRNMPTE    805
|Q8GSM3|HvLOX2.2|        DKQDEPWWPELDCHESLVQVLATIMWVTSAHHAAVNFGQYPMAGYVPNHPSIARRNMPCE    806
|P38419|OsLOX7|          DKKDAPWWPKLDSPESLAHTLTTIVWVAAAHHAAVNFGQYDFGGYFPNRPSIARTVMPVE    801
|P38418|LOX2|            DKKDEPWWPVLKTQDDLIGVVTTIAWVTSGHHAAVNFGQYGYGGYFPNRPTTTRIRMPTE    774
|Q8GV01|BnLOX2|          DKKNEPWWPVLKTQDDLIEVVTTIAWVASGHHAAVNFGQYGYGGYFPNRPTTSRIKMPVE    770
|Q8GSM2|HvLOX2.3|        DKKDEPWWPVCDTKENLVQILTIIMWVTSGHHAAVNFGQYHYAGYFPNRPTVVRRNIPVE    774
|A1XCI5|ZmLOX10|         DKKDEPWWPVLDTRDSLVETLTTIMWVTSGHHSAVNFGQYHFAGYFPNRPTTIRKNMPVE    782
|A1XCI6|ZmLOX11|         DKKDEPWWPVLDTRDSLAETLTTIMWVTSGHHSAVNFGQYHFGGYFPNRPTTIRKSMPVE    787
|E3NYV3|OeLOX2|          DKKDESWWPVLETPGDLIGILTTIIWVASGHHAAVNFGQYDFAGYFPNRPTITRTNMPTE    779
|Q6X5R6|NaLOX2|          DKKDEPWWPELKTPDDLIGIITTIVWVTSGHHAAVNFGQYSYAGYFPNRPTTARAKMPTE    778
|Q96573|SlLOXc|          DKKDEPWWPELKTPNDLIGIITTIVWVTSGHHAAVNFGQYSYGGYFPNRPTTARSKMPTE    774
|O24370|StLOX2|          DKRDEPWWPELKTPNDLIGIITTIVWVTSGHHAAVNFGQYSYAGYFPNRPTVARSKMPTE    777
|P08170|SLOX|            DLKDKPWWPKLQTLEDLVEVCLIIIWIASALHAAVNFGQYPYGGLIMNRPTASRRLLPEK    718
|P14856|PsLOX2|          DLKDKPWWPKLQTFDELVEVCTIIIWTASALHAAVNFGQYPYGGLILNRPTLSRRLLPEE    744
|Q9CAG3|LOX6|            DKKDEPWWPKLNTTQDLSQILTNMIWIASGQHAAINFGQYPFGGYVPNRPTLLRKLIPQE    795
|A0A176VER0|MpLOX|       DKKDESWWPALESEADLTRILTIMMWIASGFHAAVNFGQFAYAGYVPNKPTHVRRLVPEP    825
```

```
|A1XCI4|ZmLOX9|             DIRHAPWWPSLSTPGDLASILTTLVWLASAQHAALNFGQYPLGGYVPNRPPLMRRLLPDP    800
|Q9LNR3|LOX3|               DLRDADWWPELSTVDDLVSILTTLIWLASAQHAALNFGQYPYGGYVPNRPPLMRRLIPDE    798
|Q9FNX8|LOX4|               DHRDAEWWPKLSTVEDLVSVITTIIWLASAQHAALNFGQYPYGGYVPNRPPLMRRLIPDE    805
|A0A078ILA7|BnLOX|          DHRDADWWPELSTVDDLVSIITTIVWLASAQHAALNFGQYPYGGYVPNRPPLMRQLIPDE    798
|E3NYV2|OeLOX1|             DLRHADWWPTLATPEDLTSILTTIIWLASAQHAALNFGQYPYGGYVPNRPPLMRRLLPDE    792
|Q6X5R5|NaLOX3|             DLRNEEWWPTLATPEDLISILTTLIWLASAQHASLNFGQYPYGGYVPNRPPLMRRLIPDE    792
|Q96574|SlLOXd|             DLRNEDWWPTLATPEDLISILTTLIWLASAQHAALNFGQYPYSGYVPNRPPLMRRLIPDE    787
|O24371|StLOX3|             DLRNEEWWPTLATPEDLISILTTLIWLASAQHAALNFGQYPYGGYVPNRPPLMRRLIPDE    793
|A0A1Q4S425|CyLOX|          RVPDFGENGSIQTLNYLIDAATLIIFTATAQHGAVNYPQKDLMSYAAAAPLAGYMPASTL    537
|O16025|PhAOSLOX|           GHQDHGVPASFESREQLKEVLTSLVFTFSCQHAAVNFSQKDHYGFTPNAPAVLRHPPPKK    971
|P18054|hLOX12|             QAQDRGFPVSFQSQSQLCHFLTMCVFTCTAQHAAINQGQLDWYAWVPNAPCTMRMPPPTT    568
|P16050|hLOX15|             GAQDRGFPVSLQARDQVCHFVTMCIFTCTGQHASVHLGQLDWYSWVPNAPCTMRLPPPTT    568
|P09917|hLOX5|              GRKSSGFPKSVKSREQLSEYLTVVIFTASAQHAAVNFGQYDWCSWIPNAPPTMRAPPPTA    579
|O15296|hLOX15B|            NQESSGIPSSLETREALVQYVTMVIFTCSAKHAAVSAGQFDSCAWMPNLPPSMQLPPPTS    581
                                            :           :  * ::   *


|A0A4D6T4S4|AaeLOX|         LR--------ELTAYKEADVLKALPLQSRNTWLLMAQVPYLLSAEVEPENNIVSYAKNAS    657
|A0A3G6V563|LiLOX|          E---SDDFKTLAAQDAETAILPFLASPLQATQVMA--TLGLLSTHSENEEYINDLE---H    916
|P93184|HvLOX2.1|           I---GGDDMRDFVEAPEKVLLDTFPSQYQSAIVLA--ILDLLSTHSSDEEYMGTHE---E    857
|Q8GSM3|HvLOX2.2|           M---GPEEMLAFKAAPEKVWLDTLPSQLQTVMVMA--TLDLLSSHASDEEYMGTHQ---E    858
|P38419|OsLOX7|             E-PVDGAAMERFLDNPDQALRECFPSQVQATVVMA--VLDVLSSHSTDEEYLGGEQ---T    855
|P38418|LOX2|               D--PTDEALKEFYESPEKVLLKTYPSQKQATLVMV--TLDLLSTHSPDEEYIGEQQ---E    827
|Q8GV01|BnLOX2|             E--PTDEELKEFYEDPEKTMLKTFPSKKQATIVMV--TLDLLSAHSPDEEYLGENP---E    823
|Q8GSM2|HvLOX2.3|           E--NRDDEMKKFMARPEEVLLQSLPSQMQAIKVMA--TLDILSSHSPDEEYMGEYA---E    827
|A1XCI5|ZmLOX10|            E-GGPGEEMEKFLKQPETTLLDMLPTQMQAIKVMT--TLDILSSHSPDEEYMGEFA---E    836
|A1XCI6|ZmLOX11|            EEAGRDEEMKKFLKQPETTLLDMLPTQMQAIRVMT--TLDILSSHSPDEEYMGELA---E    842
|E3NYV3|OeLOX2|             D--PNDTEKEEFLKKPEEFILKCFPSQVQATIIMA--ILDVLSSHSPDEEYLGENI---Q    832
|Q6X5R6|NaLOX2|             D--PTDEEWENFLKRPEDALLKCFPSQMQATKVMA--ILDVLSNHSPDEEYIGEKI---E    831
|Q96573|SlLOXc|             D--PTAEEWEWFLNKPEEALLRCFPSQIQATKVMT--ILDVLSNHSPDEEYIGEKI---E    827
|O24370|StLOX2|             D--PTAEEWEWFMNKPEEALLRCFPSQIQATKVMA--ILDVLSNHSPDEEYIGEKI---E    830
|P08170|SLOX|               ----GTPEYEEMINNHEKAYLRTITSKLPTLISLS--VIEILSTHASDEVYLGQRD---N    769
|P14856|PsLOX2|             ----GTAEYDEMVKSSQKAYLRTITPKFQTLIDLS--VIEILSRHASDEVYLGQRE---N    795
|Q9CAG3|LOX6|               ----TDPDYEMFMRNPQYSFLGSLPTQLQATKVMA--VQETLSTHSPDEEYLIELRE-VQ    848
|A0A176VER0|MpLOX|          ----GTAEWREFQSNPQQFLLSMLPGQLQATVLMA--VIESLSTHSPDEEYLGQIS--HP    877
|A1XCI4|ZmLOX9|             E--RDAAEYATFMADPHRFFLNAMPGVLEATKFMA--VVDTLSTHSPDEEYLGEER---D    853
|Q9LNR3|LOX3|               ----SDPEYASFISHPEKYYFSSMPSLAQTSKFMA--VVDTLSTHSPDEEYIGERQ--QP    850
|Q9FNX8|LOX4|               ----SDPEFTSFIEDPQKYFFSSMPSLLQTTKFMA--VVDTLSTHSPDEEYIGERQ--QP    857
|A0A078ILA7|BnLOX|          ----SEPEFASFVEDPQKYFFSSLPSLLQTTKFMA--VVDTLSTHSPDEEYIGERQ--QP    850
|E3NYV2|OeLOX1|             ----NDPEYAIFHADPQKYYFSALPSLLQATKFMA--VVDTLSTHSPDEEYLGERH--HQ    844
|Q6X5R5|NaLOX3|             ----NDPEYAVFHDDPQKYFFSALPSLLQATKFMA--VVDTLSTHSPDEEYIGDRH--QP    844
|Q96574|SlLOXd|             ----NDPEYAVFLADPQKYFFSALPSLLQATKFMA--VVDTLSTHSPDEEYIGERQ--QP    839
|O24371|StLOX3|             ----NDPEYAVFLADPQKYFFSALPSLLQATKFMA--VVDTLSTHSPDEEYLGERH--QP    845
|A0A1Q4S425|CyLOX|          K---GE--------VTEQDYLNLLPPLEQAQRQLN--VLTLLGSIY--YKNLGNYS---P    579
|O16025|PhAOSLOX|           K---GE--------ATLQSILSTLPSKSQAAKAIA--TVYILTKFSEDERYLGNYS---A    1015
|P18054|hLOX12|             K---ED--------VTMATVMGSLPDVRQACLQMA--ISWHLSRRQPDMVPLGHHK---E    612
|P16050|hLOX15|             K----D--------ATLETVMATLPNFHQASLQMS--ITWQLGRRQPVMVAVGQHE---E    611
|P09917|hLOX5|              K---GV--------VTIEQIVDTLPDRGRSCWHLG--AVWALSQFQENELFLGMYP---E    623
|O15296|hLOX15B|            K---GL--------ATCEGFIATLPPVNATCDVIL--ALWLLSKEPGDQRPLGTYP---D    625
                                                             :       *         :

|A0A4D6T4S4|AaeLOX|         KSW--NPAISQAAKVLQEDVKALEESFKAISAQMDDQ-------GTKYAVLDPNVM----    704
|A0A3G6V563|LiLOX|          PYLMVGTEAYNKYKEFLSRLKQAEDTIKKRNADSQGHVIRAGPDAIHYRLMNPSSI--TE    974
|P93184|HvLOX2.1|           PAWTKDGVINQAFEEFKESTRKIVEQVDEWNNDPDRK-NRHGAGMVPYVLLRPSDGDPTD    916
|Q8GSM3|HvLOX2.2|           PAWQRDGEVDKAFQVFQKKMRDIAEQVEEWNKDDSRR-NRHGAGVVPYVLLRPLNGNP--    915
|P38419|OsLOX7|             RPWNSDAAVQAAYDGFAARLKEIEGVIDGRNKDRKLK-NRCGAGILPYQLMKPFSD----    910
|P38418|LOX2|               ASWANEPVINAAFERFKGKLQYLEGVIDERNVNITLK-NRAGAGVVKYELLKPTSE----    882
|Q8GV01|BnLOX2|             ASWAHEPVIYAAYERFKGKLQYLEGVIDERNVNVSLK-NRTGAGVVKYELLKPISE----    878
|Q8GSM2|HvLOX2.3|           PAWLAEPMVKAAFEKFSGRLKEAEGTIDMRNNNPENK-NRCGAGIVPYELLKPFSE----    882
|A1XCI5|ZmLOX10|            PSWLAEPMVKAAFEKFGGRMKEIEGFIDECNNNLDLK-NRCGAGIVPYELLKPFSK----    891
|A1XCI6|ZmLOX11|            PSWLEEPMVKAAFEKFGGRMKEIEGFIDECNNNPELR-NRCGAGIVPYELLKPFSK----    897
|E3NYV3|OeLOX2|             PYWKDDKYINAIFEQFAGKVKEIEGIIDARNTNCDLM-NRSGAGVVPYQLLKPFSE----    887
|Q6X5R6|NaLOX2|             PYWAEDPVINAAFEKFSGRLKELEGIIDGRNADSNLM-NRNGAGVVPYELLKPFSG----    886
|Q96573|SlLOXc|             PYWAEDPVINAAFEVFSGKLKELEGIIDARNNDSKLN-NRNGAGVMPYELLKPYSE----    882
|O24370|StLOX2|             PYWAEDPVINAAFEVFSGKLKELEGIIDARNNDSKLS-NRNGAGVMPYELLKPYSE----    885
|P08170|SLOX|               PHWTSDSKALQAFQKFGNKLKEIEEKLVRRNNDPSLQGNRLGPVQLPYTLLYPSSE----    825
|P14856|PsLOX2|             PHWTSDSKALQAFQKFGNKLAEIEAKLTNKNNDPSLY-HRVGPVQLPYTLLHPSSK----    850
|Q9CAG3|LOX6|               RHWFQDEQVVKYFNKFSEELVKIEKTINERNKDKKLK-NRTGAGMPPYELLLPTSP----    903
|A0A176VER0|MpLOX|          -KWLGCPDGLQAFRRFQQSVSAIDREITARNNNAHLR-NRNGAGILPYELLLPTSG----    931
|A1XCI4|ZmLOX9|             EPWTGDAAAVAAHDMFTADVRRAEEAIDSRNADQRRK-NRCGAGVLPYELLAPSSP----    908
|Q9LNR3|LOX3|               SIWTGDAEIVEAFYGFAAEIGRIEKEIEKRNADPDRR-NRCGAGVLPYELLVPSSE----    905
|Q9FNX8|LOX4|               SIWTGDAEIVDAFYGFSAEIGRIEKEIDKRNRDPSRR-NRCGAGVLPYELMAPSSE----    912
|A0A078ILA7|BnLOX|          SIWTGDAEIVDAFYGFSAEIGRIEQEIEKRNKDPSRR-NRCGAGVLPYELMAPSSE----    905
|E3NYV2|OeLOX1|             SIWSRDAEVIESFYEFSAEIRRIEKEIEKRNVDSTLR-NRSGAGVLPYELLAPSSG----    899
|Q6X5R5|NaLOX3|             STWTGDAEIVEAFYDFSSEIRRIEKEIDDRNADTRLR-NRCGAGVLPYELLAPSSG----    899
|Q96574|SlLOXd|             STWTGDAEIVEAFYKFSAEIGRIEKEIDERNADTNLK-NRCGAGVLPYELLAPSSG----    894
|O24371|StLOX3|             STWTGDAEIVEAFYKFSAEIGRIEKEIDERNANTKLK-NRCGAGVLPYELLAPSSG----    900
|A0A1Q4S425|CyLOX|          GHF-PAQ-VTPLLDKFHKNLIQIEEIINDRNLH-R----------PTYEYLLPSRI----    622
|O16025|PhAOSLOX|           TAW-EDKDALDAINRFQDKLEDISKKIKQRNENLE----------VPYIYLLPERI----    1060
|P18054|hLOX12|             KYF-SGPKPKAVLNQFRTDLEKLEKEITARNEQLD----------WPYEYLKPSCI----    657
|P16050|hLOX15|             EYF-SGPEPKAVLKKFREELAALDKEIEIRNAKLD----------MPYEYLRPSVV----    656
|P09917|hLOX5|              EHF-IEKPVKEAMARFRKNLEAIVSVIAERNKKKQ----------LPYYYLSPDRI----    668
|O15296|hLOX15B|            EHF-TEEAPRRSIATFQSRLAQISRGIQERNQGLV----------LPYTYLDPPLI----    670
                                           :          .   .                *  : *

|A0A4D6T4S4|AaeLOX|         --------------ATSILI      710
|A0A3G6V563|LiLOX|          NGDVQRQGVTMSGIPTSVSM      994
|P93184|HvLOX2.1|           GDPTDEKMVMEMGIPNSISI      936
|Q8GSM3|HvLOX2.2|           ---MDAKTVMEMGIPNSISI      932
|P38419|OsLOX7|             ------SGVTGMGIPNSTSI      924
|P38418|LOX2|               ------HGVTGMGVPYSISI      896
|Q8GV01|BnLOX2|             ------PGVTGMGVPYSVSI      892
|Q8GSM2|HvLOX2.3|           ------PGVTGRGIPNSISI      896
|A1XCI5|ZmLOX10|            ------PGVTGRGIPSSISI      905
|A1XCI6|ZmLOX11|            ------PGVTGRGIPNSISI      911
|E3NYV3|OeLOX2|             ------AGVTGKGVPNSVSI      901
|Q6X5R6|NaLOX2|             ------PGVTGKGVPYSISI      900
|Q96573|SlLOXc|             ------PGVTGKGVPYSISI      896
|O24370|StLOX2|             ------PGVTGKGVPYSISI      899
|P08170|SLOX|               ------EGLTFRGIPNSISI      839
```

```
|P14856|PsLOX2|          ------EGLTFRGIPNSISI      864
|Q9CAG3|LOX6|            ------HGVTGRGIPNSISI      917
|A0A176VER0|MpLOX|       ------PGITGRGVPNSISI      945
|A1XCI4|ZmLOX9|          ------PGVTCRGVPNSISI      922
|Q9LNR3|LOX3|            ------PGVTCRGVPNSVSI      919
|Q9FNX8|LOX4|            ------PGVTCRGVPNSVSI      926
|A0A078ILA7|BnLOX|       ------PGVTCRGVPNSVSI      919
|E3NYV2|OeLOX1|          ------PGVTCRGVPNSVSI      913
|Q6X5R5|NaLOX3|          ------PGVTCRGVPNSVSI      913
|Q96574|SlLOXd|          ------PGVTCRGVPNSVSI      908
|O24371|StLOX3|          ------PGVTCRGVPNSVSI      914
|A0A1Q4S425|CyLOX|       --------------PQSINI      628
|O16025|PhAOSLOX|        --------------PNGTAI      1066
|P18054|hLOX12|          --------------ENSVTI      663
|P16050|hLOX15|          --------------ENSVAI      662
|P09917|hLOX5|           --------------PNSVAI      674
|O15296|hLOX15B|         --------------ENSVSI      676
                                        .  :
```

1: |A0A4D6T494|AaeLOX|	100.00	21.64	20.15	19.57	19.66	22.36	21.59	20.92	22.05	21.19	22.73	22.70	21.13	21.44	20.09	19.66	20.76	22.51	22.49	21.68	21.34	21.42	22.68	23.44	22.87	22.87	20.91	18.51	20.07	21.08	19.23	18.96
2: |A0A3G6V563|LiLOX|	21.64	100.00	35.63	33.57	34.45	35.37	36.39	38.36	37.41	37.83	36.71	36.63	34.60	34.84	35.49	34.72	36.97	33.72	35.24	36.65	36.21	36.27	36.15	36.48	36.46	35.60	22.59	21.82	24.73	23.98	23.54	24.03
3: |P93184|HvLOX2.1|	20.15	35.63	100.00	72.08	52.55	49.59	48.83	54.24	53.76	52.37	53.02	54.27	52.22	53.39	40.19	39.98	43.07	41.19	42.05	42.50	42.45	41.76	41.97	42.41	42.89	42.58	21.72	23.68	24.34	23.75	26.85	23.79
4: |Q8GSM3|HvLOX2.2|	19.57	33.57	72.08	100.00	50.00	47.78	47.42	52.84	51.67	50.63	49.13	51.17	49.30	50.58	39.51	39.93	41.47	40.86	40.57	42.63	42.81	42.58	42.45	42.31	42.91	42.59	22.65	22.90	23.77	24.12	27.20	24.20
5: |P38419|OsLOX7|	19.66	34.45	52.55	50.00	100.00	51.54	52.74	53.88	55.30	54.22	55.41	54.86	54.68	55.40	44.33	43.24	46.14	44.71	44.19	45.29	46.46	45.98	45.46	45.82	45.57	46.05	23.79	23.65	26.45	23.67	26.32	24.22
6: |P38418|LOX2|	22.36	35.37	49.59	47.78	51.54	100.00	76.61	52.08	52.58	51.94	56.34	57.98	57.34	58.06	43.93	43.67	46.82	44.86	43.90	45.04	45.19	45.10	43.58	45.48	45.51	45.77	24.20	23.16	25.55	26.99	26.39	25.19
7: |Q8GV01|BnLOX2|	21.59	36.39	48.83	47.42	52.74	76.61	100.00	52.32	53.97	52.98	54.88	57.19	56.66	56.81	42.71	42.79	44.93	44.46	43.49	44.92	44.60	44.51	45.20	46.78	45.76	46.38	22.48	23.46	26.10	25.67	27.88	26.06
8: |Q8GSM2|HvLOX2.3|	20.92	38.36	54.24	52.84	53.88	52.08	52.32	100.00	73.29	71.91	56.86	58.08	55.29	57.41	41.86	42.49	47.69	46.98	43.78	45.04	44.13	43.68	44.82	46.12	46.10	46.24	24.92	24.03	27.12	26.22	27.33	25.67
9: |A1XCI5|ZmLOX10|	22.05	37.41	53.76	51.67	55.30	52.58	53.97	73.29	100.00	88.61	57.13	59.36	57.57	58.64	42.16	42.91	46.99	48.10	43.02	45.40	44.61	44.64	44.91	45.32	45.06	45.43	23.82	22.77	26.41	25.82	26.93	23.87
10: |A1XCI6|ZmLOX11|	21.19	37.83	52.37	50.63	54.22	51.94	52.98	71.91	88.61	100.00	55.69	57.58	56.01	56.74	41.29	42.91	46.83	46.68	42.89	45.07	44.17	44.55	44.65	45.52	44.80	45.29	24.20	22.14	26.56	25.82	26.16	23.40
11: |E3NYV3|OeLOX2|	22.73	36.71	53.02	49.13	55.41	56.34	54.88	56.86	57.13	55.69	100.00	67.45	64.94	66.97	43.78	45.25	48.23	48.68	46.86	49.35	48.30	47.29	48.19	48.36	48.06	48.36	24.41	23.87	26.37	25.94	27.36	26.95
12: |Q6X5R6|NaLOX2|	22.70	36.63	54.27	51.17	54.86	57.98	57.19	58.08	59.36	57.58	67.45	100.00	82.11	83.97	42.27	43.39	48.76	48.73	45.73	48.46	48.47	48.40	49.06	48.42	49.88	49.36	22.56	23.22	26.56	26.29	25.70	23.71
13: |Q96573|SlLOXc|	21.13	34.60	52.22	49.30	54.68	57.34	56.66	55.29	57.57	56.01	64.94	82.11	100.00	92.41	42.38	43.12	47.50	46.81	44.44	47.51	46.64	46.67	46.43	45.54	45.81	46.01	22.22	22.01	26.52	25.78	25.50	22.90
14: |O24370|StLOX2|	21.44	34.84	53.39	50.58	55.40	58.06	56.81	57.41	58.64	56.74	66.97	83.97	92.41	100.00	42.50	43.87	48.10	48.15	45.96	48.69	48.29	48.34	48.07	47.18	47.11	47.30	22.05	22.49	25.90	25.94	25.35	23.36
15: |P08170|SLOX|	20.09	35.49	40.19	39.51	44.33	43.93	42.71	41.86	42.16	41.29	43.78	42.27	42.38	42.50	100.00	78.61	42.74	44.43	42.93	45.95	45.83	45.37	45.52	45.95	44.74	45.22	24.16	24.55	23.74	24.72	25.19	24.80
16: |P14856|PsLOX2|	19.66	34.72	39.98	39.93	43.24	43.67	42.79	42.49	42.91	42.91	45.25	43.39	43.12	43.87	78.61	100.00	42.50	43.68	43.96	45.77	45.77	45.80	46.19	46.14	45.65	46.14	25.55	23.58	24.42	25.39	26.30	25.15
17: |Q9CAG3|LOX6|	20.76	36.97	43.07	41.47	46.14	46.82	44.93	47.69	46.99	46.83	48.23	48.76	47.50	48.10	42.74	42.50	100.00	47.09	47.85	50.68	51.07	50.11	52.39	52.56	51.71	52.61	23.83	24.07	26.83	25.94	26.08	25.08
18: |A0A176VER0|MpLOX|	22.51	33.72	41.19	40.86	44.71	44.86	44.46	46.98	48.10	46.68	48.68	48.73	46.81	48.15	44.43	43.68	47.09	100.00	49.89	48.62	46.75	47.13	49.00	50.22	49.27	49.61	22.84	24.01	26.37	25.47	26.55	25.55
19: |A1XCI4|ZmLOX9|	22.49	35.24	42.05	40.57	44.19	43.90	43.49	43.78	43.02	42.89	46.86	45.73	44.44	45.96	42.93	43.96	47.85	49.89	100.00	63.83	63.07	61.85	64.52	65.03	64.51	64.55	22.82	23.25	26.91	24.45	26.46	25.47
20: |Q9LNR3|LOX3|	21.68	36.65	42.50	42.63	45.29	45.04	44.92	45.04	45.40	45.07	49.35	48.46	47.51	48.69	45.95	45.77	50.68	48.62	63.83	100.00	84.08	83.10	73.91	75.61	74.36	73.94	24.11	24.73	26.76	26.80	26.63	25.00
21: |Q9FNX8|LOX4|	21.34	36.21	42.45	42.81	46.46	45.19	44.60	44.13	44.61	44.17	48.30	48.47	46.64	48.29	45.83	45.77	51.07	46.75	63.07	84.08	100.00	92.16	74.14	74.86	73.72	73.56	22.77	24.49	27.34	26.45	26.43	25.59
22: |A0A078ILA7|BnLOX|	21.42	36.27	41.76	42.58	45.98	45.10	44.51	43.68	44.64	44.55	47.29	48.40	46.67	48.34	45.37	45.80	50.11	47.13	61.85	83.10	92.16	100.00	73.27	73.66	73.18	72.91	23.17	24.04	27.17	26.58	26.09	25.08
23: |E3NYV2|OeLOX1|	22.68	36.15	41.97	42.45	45.46	43.58	45.20	44.82	44.91	44.65	48.19	49.06	46.43	48.07	45.52	46.19	52.39	49.00	64.52	73.91	74.14	73.27	100.00	83.89	82.13	82.25	23.70	25.14	26.37	26.09	26.08	25.08
24: |Q6X5R5|NaLOX3|	23.44	36.48	42.41	42.31	45.82	45.48	46.78	46.12	45.32	45.52	48.36	48.42	45.54	47.18	45.95	46.14	52.56	50.22	65.03	75.61	74.86	73.66	83.89	100.00	90.64	91.12	23.87	24.06	25.90	26.25	25.93	25.86
25: |Q96574|SlLOXd|	22.87	36.46	42.89	42.91	45.57	45.51	45.76	46.10	45.06	44.80	48.06	49.88	45.81	47.11	44.74	45.65	51.71	49.27	64.51	74.36	73.72	73.18	82.13	90.64	100.00	96.59	23.23	23.85	25.62	25.82	25.97	26.05
26: |O24371|StLOX3|	22.87	35.60	42.58	42.59	46.05	45.77	46.38	46.24	45.43	45.29	48.36	49.36	46.01	47.30	45.22	46.14	52.61	49.61	64.55	73.94	73.56	72.91	82.25	91.12	96.59	100.00	23.74	24.44	25.94	25.67	26.12	26.37
27: |A0A1Q4S425|CyLOX|	20.91	22.59	21.72	22.65	23.79	24.20	22.48	24.92	23.82	24.20	24.41	22.56	22.22	22.05	24.16	25.55	23.83	22.84	22.82	24.11	22.77	23.17	23.70	23.87	23.23	23.74	100.00	28.74	25.81	24.66	27.20	28.03
28: |O16025|PhAOSLOX|	18.51	21.82	23.68	22.90	23.65	23.16	23.46	24.03	22.77	22.14	23.87	23.22	22.01	22.49	24.55	23.58	24.07	24.01	23.25	24.73	24.49	24.04	25.14	24.06	23.85	24.44	28.74	100.00	32.22	30.59	39.55	34.63
29: |P18054|hLOX12|	20.07	24.73	24.34	23.77	26.45	25.55	26.10	27.12	26.41	26.56	26.37	26.56	26.52	25.90	23.74	24.42	26.83	26.37	26.91	26.76	27.34	27.17	26.37	25.90	25.62	25.94	25.81	32.22	100.00	65.56	42.12	37.88
30: |P16050|hLOX15|	21.08	23.98	23.75	24.12	23.67	26.99	25.67	26.22	25.82	25.82	25.94	26.29	25.78	25.94	24.72	25.39	25.94	25.47	24.45	26.80	26.45	26.58	26.09	26.25	25.82	25.67	24.66	30.59	65.56	100.00	40.06	38.24
31: |P09917|hLOX5|	19.23	23.54	26.85	27.20	26.32	26.39	27.88	27.33	26.93	26.16	27.36	25.70	25.50	25.35	25.19	26.30	26.08	26.55	26.46	26.63	26.43	26.09	26.08	25.93	25.97	26.12	27.20	39.55	42.12	40.06	100.00	42.96
32: |O15296|hLOX15B|	18.96	24.03	23.79	24.20	24.22	25.19	26.06	25.67	23.87	23.40	26.95	23.71	22.90	23.36	24.80	25.15	25.08	25.55	25.47	25.00	25.59	25.08	25.08	25.86	26.05	26.37	28.03	34.63	37.88	38.24	42.96	100.00

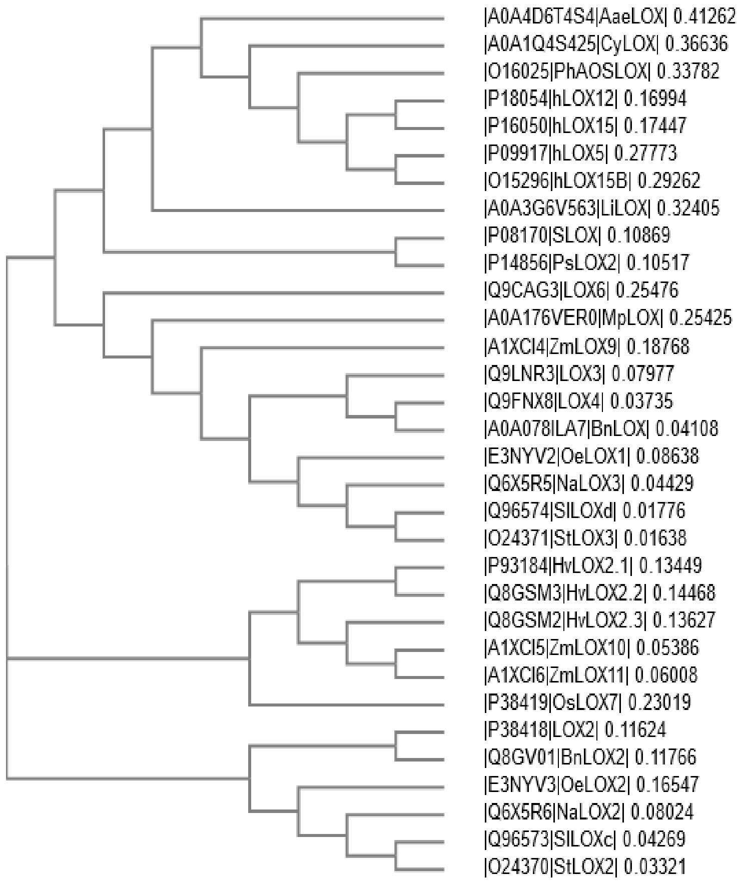

Word file S1: Clustal omega sequence analysis of selected lipoxygenases. Multiple sequence alignments (complete) of 13-LOX with various 13-LOXs and LOXs belonging to fungi (Agrocybe aegerita, Aae), algae (Lobosphaera incisa, Li), cyanobacteria (Calothrix sp. HK-06, Cy), moss (Marchantia polymorpha, Mp), anthozoa (Plexaura homomalla, Ph), monocot (Oryza sativa Os, zea mays Zm, Hordeum vulgare, Hv) and dicot (soybean S, Brassica napus Bn, Olea europaea Oe, Pisum sativum Ps, Nicotiana attenuata, Na, Solanum lycopersicum Sl, Solanum tuberosum St) plants and humans (h). Codes represent Uniprot/SwissProtIDs. (*), conserved amino acids; (:), conservative replacement; (.), half conservative replacement. Orange boxes suggest LOX3/4 differentiation amino acids from LOX2/6 type. Green, amino acids involved in At-13-LOX inter-domain stabilization, red, putative Ca^{2+} ligands in human and coral LOXs. Black, regio- and stereo determining and iron cofactor ligating amino acids only shown for At-13-LOXs. Per default, Clustal omega online tool (www.ebi.ac.uk/Tools/msa/clustalo/) also provides identity matrix of LOXs and unrooted phylogenetic tree of analysed LOXs. These are shown below alignment, further details see text.
